# Model-Based Analysis of HER Activation in Cells Co-Expressing EGFR, HER2 and HER3

**DOI:** 10.1371/journal.pcbi.1003201

**Published:** 2013-08-22

**Authors:** Harish Shankaran, Yi Zhang, Yunbing Tan, Haluk Resat

**Affiliations:** 1 Computational Biology and Bioinformatics Group, Pacific Northwest National Laboratory, Richland, Washington, United States of America; 2 School of Electrical Engineering and Computer Science, Washington State University, Pullman, Washington, United States of America; Johns Hopkins University, United States of America

## Abstract

The HER/ErbB family of receptor tyrosine kinases drives critical responses in normal physiology and cancer, and the expression levels of the various HER receptors are critical determinants of clinical outcomes. HER activation is driven by the formation of various dimer complexes between members of this receptor family. The HER dimer types can have differential effects on downstream signaling and phenotypic outcomes. We constructed an integrated mathematical model of HER activation, and trafficking to quantitatively link receptor expression levels to dimerization and activation. We parameterized the model with a comprehensive set of HER phosphorylation and abundance data collected in a panel of human mammary epithelial cells expressing varying levels of EGFR/HER1, HER2 and HER3. Although parameter estimation yielded multiple solutions, predictions for dimer phosphorylation were in agreement with each other. We validated the model using experiments where *pertuzumab* was used to block HER2 dimerization. We used the model to predict HER dimerization and activation patterns in a panel of human mammary epithelial cells lines with known HER expression levels in response to stimulations with ligands EGF and HRG. Simulations over the range of expression levels seen in various cell lines indicate that: i) EGFR phosphorylation is driven by HER1-HER1 and HER1-HER2 dimers, and not HER1-HER3 dimers, ii) HER1-HER2 and HER2-HER3 dimers both contribute significantly to HER2 activation with the EGFR expression level determining the relative importance of these species, and iii) the HER2-HER3 dimer is largely responsible for HER3 activation. The model can be used to predict phosphorylated dimer levels for any given HER expression profile. This information in turn can be used to quantify the potencies of the various HER dimers, and can potentially inform personalized therapeutic approaches.

## Author Summary

A family of cell surface molecules called the HER receptor family plays important roles in normal physiology and cancer. This family has four members, HER1-4. These receptors convert signals received from the extracellular environment into cell decisions such as growth and survival – a process termed signal transduction. In particular, HER2 and HER3 are over-expressed in a number of tumors, and their expression levels are associated with abnormal growth and poor clinical prognosis. A key step in HER-mediated signal transduction is the formation of dimer complexes between members of this family. Different dimer types have different potencies for activating normal and aberrant responses. Prediction of the dimerization pattern for a given HER expression level may pave the way for personalized therapeutic approaches targeting specific dimers. Towards this end, we constructed a mathematical model for HER dimerization and activation. We determined unknown model parameters by analyzing HER activation data collected in a panel of human mammary epithelial cells that express different levels of the HER molecules. The model enables us to quantitatively link HER expression levels to receptor dimerization and activation. Further, the model can be used to support additional quantitative investigations into the basic biology of HER-mediated signal transduction.

## Introduction

The HER family (Human Epidermal growth factor Receptor, also known as the ErbB family) of cell surface receptors plays critical roles in normal cell physiology, development, and cancer pathophysiology [1], [2], [3], [4]. The family consists of the four closely related transmembrane receptor tyrosine kinases HER1 (EGFR), HER2 (NEU), HER3 and HER4, which when activated initiate downstream signaling, and affect a range of cellular decisions including proliferation, survival and motility [4], [5].

The HER receptor expression profile is a critical determinant of cell behavior [6], [7], and outcomes in cancer pathology. Overexpression of EGFR, HER2 and HER3 is associated with decreased survival in cancer, while HER4 overexpression is correlated with increased survival [8], [9]. HER2 is overexpressed in 25–30% of all breast cancers, as well as in other solid tumors [10], [11] and is associated with poor prognosis [8], [12], [13], [14]. While this has led to the development of a range of therapeutics targeting the HER2 receptor [15], the use of these drugs can often lead to resistance through a diverse set of mechanisms [16]. The overexpression of HER family members and their ligands are key compensatory mechanisms responsible for the development of resistance to HER-targeted therapies [17], [18], [19], [20]. In particular, the importance of HER3 expression in driving tumorigenesis [21], [22], [23], [24], and in the development of drug resistance [17], [25] is being increasingly recognized leading to an increased focus on HER3-targeted therapies [3], [15], [26], [27], [28]. While the importance of HER expression levels has been established for clinical prognosis and drug resistance, the mechanistic link between receptor expression, HER activation and downstream consequences is not as clear yet.

HER activation is a complex process involving multiple sequential steps, which in general are as follows: the specific binding of ligands (growth factors) to HER receptors leads to conformational changes promoting dimerization between members of the family [29], [30], [31]; dimerization leads to the trans-phosphorlyation of receptor cytoplasmic tails via the kinase activities of the partners in the dimer leading to downstream signaling [31]. Although the HER receptors are homologous, there are key differences in their behavior. EGFR [32], HER3 [33], and HER4 [34] undergo ligand-induced conformational changes promoting dimerization. In contrast, HER2, which has no known ligand, has a structure that enables constitutive dimerization [35], [36]. HER3, on the other hand has impaired kinase activity, but can allosterically facilitate a partner's kinase activity following dimerization [37]. Further, HER receptors have different trafficking properties with EGFR showing increased ligand-induced internalization and degradation compared to the other members of the family [38]. All of these aspects have a bearing on the number and types of dimers that are formed between the HER receptors following ligand addition. Since the HER dimerization pattern is an important determinant of the consequences of HER activation [39], [40] it is important to quantitatively predict this as a function of the receptor expression profile.

Mathematical models have been extensively applied to understand HER activation dynamics [1], [41], [42]. Recent efforts have focused on a quantitative understanding of the interactions between multiple members of the HER family [43], [44], [45], [46]. Birtwistle et al. constructed a mathematical model for the early events (0 to 30 min) in HER activation and downstream signaling in cells coexpressing all four HER receptors [45]. They parameterized their model using HER1, HER2, Erk and Akt activation data in response to EGF and HRG stimulation in MCF-7 cells. Chen et al. constructed a more expanded model for receptor activation and signaling over longer time frames (0 to 120 min) and parameterized it using HER1, Erk and Akt activation data in response to EGF and HRG stimulation in three different cell lines – A431, H1666 and H3255 cells [43]. In each of these two manuscripts, the authors note problems with regards to parameter identifiability given the size of the models [43], [45]. Hendriks et al. focused on HER activation alone in cells expressing HER1, HER2 and HER3 [44]. They assumed parameter values based on the literature and compared simulations with receptor activation data collected in the H292 lung carcinoma cell line [44].

We have recently developed a panel of Human Mammary Epithelial (HME) cells that co-express EGFR with varying levels of HER2 and HER3 [47]. HME cells, like many epithelium-derived cell types require EGFR activation for proliferation and migration [48], and are an excellent system for developing physiologically relevant models of HER signaling. Importantly, our cell line library enables us to study the effects of varying HER expression levels in a common cellular background. We have published data on HER1-3, Erk and Akt activation in these cell lines for single doses of EGF and HRG [47]. Here, we focus further on the quantitative aspects of HER activation. We collect an expanded dataset for receptor activation that includes measurements of total and internal HER phosphorylation and HER receptor mass in four distinct HME cell lines in response to a range of EGF and HRG doses. We have identified the appropriate modeling approach (choices for model scope, granularity, etc.) for analyzing such datasets through a comprehensive model-based analysis of EGFR activation in cells that predominantly express this receptor alone [49]. Here, we expand this model by considering the co-expression of EGFR, HER2 and HER3, and parameterize it using receptor activation data collected in our cell line library. We explicitly consider model identifiability and show that the model can predict the dimer phosphorylation levels given the receptor expression level of a cell line. We note that the fourth member of the HER family is also very important in cancer [50] and should be included for completeness. However, our gene expression and proteomics studies (unpublished data) indicated that used HME cells do not express HER4. Therefore, it was not considered in this study.

## Results

### Mathematical model for HER activation

**Figure 1:**
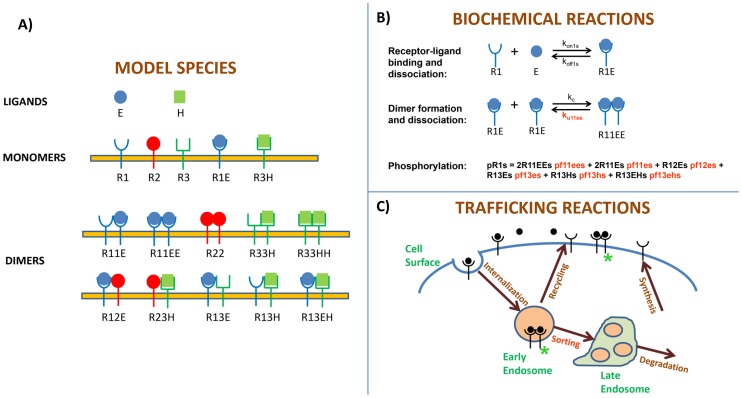
Schematic description of the mathematical model for HER activation. **A**) Species included in the mathematical model. The model consists of two distinct ligands, the EGFR ligand EGF (E) and the HER3 ligand HRG (H); five distinct receptor monomers including ligand-free and bound species; and 10 distinct dimer types that can form following addition of EGF and/or HRG. **B**) Illustrative examples of the biochemical reactions in the model. Receptor-ligand binding and dimerization are modeled explicitly using mass action kinetics (see cartoons). The level of phosphorylated HER1-3 is calculated as a linear combination of the contributions from various dimer species with dimer-specific phosphorylation factors (*pf*s) accounting for the relative contribution of each species. An illustrative expression is shown for calculating the level of phosphorylated EGFR at the cell surface. **C**) The receptor trafficking portion of the model. The model consists of three compartments: cell surface, early and late endosomes. Species are internalized from the cell surface into the early endosome from where they are either recycled back to the cell surface or sorted to late endosomes. Species within late endosomes are eventually degraded. Newly synthesized receptors are added to the cell surface. The unknown parameters in the model are the dimer dissociation rates, the *pf* values for the various dimer types, and a single parameter related to sorting (highlighted in red; see text for details).

Our objectives here are to quantitatively link HER (specifically HER1-3) expression levels to receptor activation, and to understand how differential interactions between the members of the HER family drive the process. Towards this end we constructed a parsimonious mathematical model for HER dimerization and receptor activation (Figure 1, see Methods for details), and parameterized it using the appropriate experimental datasets. The model includes the ligands EGF and HRG, ligand-bound and unbound receptor monomers, as well as the feasible combinations of receptor homo- and hetero-dimers (Figure 1A). The reversible biochemical reactions of receptor-ligand binding and dimer formation are represented explicitly via mass action kinetics (Figure 1B). As in our recent manuscript [49], we expressed the level of phosphorylated HER1-3 as a linear combination of the contributions from various dimer species with dimer-specific phosphorylation factors (*pf*s in Figure 1B) accounting for the relative contribution of each species. The *pf* can be thought of as a lumped phosphorylation efficiency factor that combines the characteristics of all possible tyrosine sites in a dimer [49]. It enables us to calculate the HER1-3 phosphorylation signals emanating from the various dimer types. The model includes 3 compartments: the cell surface, early endosomes and late endosomes. Biochemical reactions are allowed to occur in the first 2 compartments, while the late endosome is assumed to be a site for the accumulation of dephosphorylated receptors prior to degradation (Methods, and also [49]).

In all, the model consists of 51 species and 140 parameters. Given these parameter values, and a specified HER1-3 expression level, the model can be used to predict activated levels of HER1-3 at the cell surface and interior as a function of time in response to various concentrations of EGF and HRG. Values for several model parameters including receptor-ligand binding rates, receptor internalization, recycling and degradation rates are available in the literature (Methods; Tables S1, S2, S3 in Text S1). With these in place, there are 47 unknown model parameters (highlighted in red in Figure 1) that include: the compartment-specific dissociation rates for various receptor dimers, the compartment-specific phosphorylation factors that define the contribution of various dimers to the HER1-3 phosphorylation levels, and a parameter that defines how species are sorted (distributed) between the early and late endosomes (Methods).

### Data collection and model fitting

**Figure 2:**
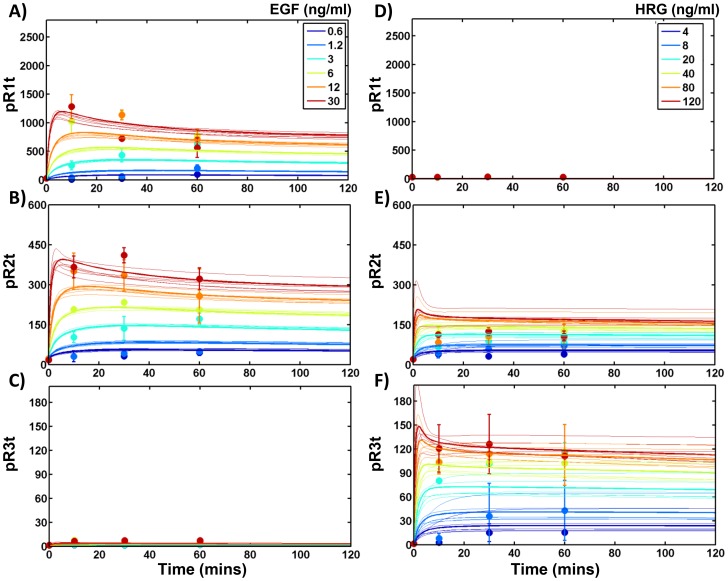
HER phosphorylation in response to various doses of EGF and HRG. **A–C**) Experimental data (markers) and model predictions (lines) for EGFR (panel A), HER2 (B) and HER3 (C) phosphorylation in the HER2+3+ cells are presented as a function of time in response to the indicated doses of EGF. For each ligand dose, model predictions using each of the 7 representative parameter sets (see text) are shown (multiple lines with the same color). Of these, predictions with the best fit parameter set are presented as dark lines. For the experimental data, mean and standard deviations (SD) calculated based on multiple replicates (N> = 2) are presented. **D–F**) EGFR (panel D), HER2 (E) and HER3 (F) phosphorylation levels in response to the indicated doses of HRG. Experimental data (markers) is presented along with model predictions using the 7 distinct parameter sets as in panels A–C. Receptor phosphorylation levels are reported in arbitrary units in all panels as normalized in the ELISA experiments.

**Figure 3:**
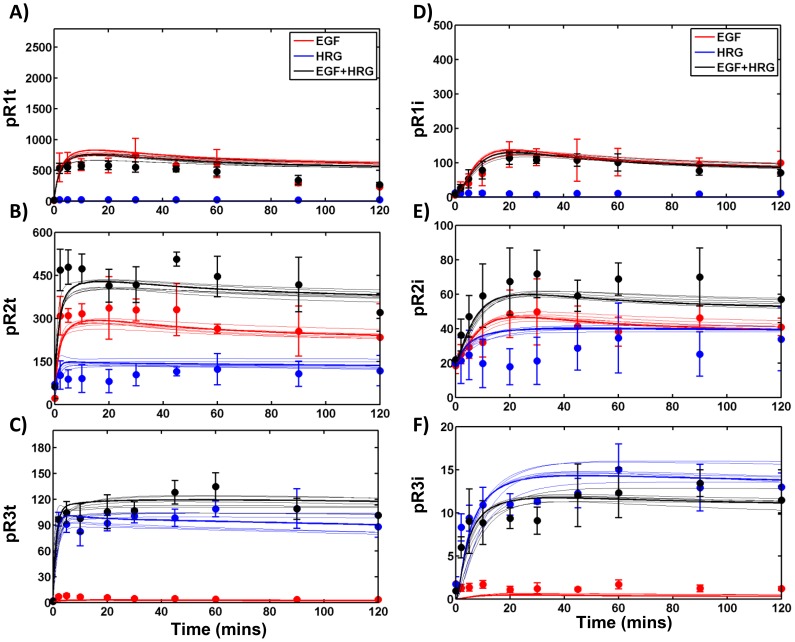
Total and internal HER phosphorylation in response to EGF and/or HRG. **A–C**) Experimental data (markers) and model predictions (lines) for total EGFR (A), HER2 (B) and HER3 (C) phosphorylation in the HER2+3+ cells in response to 12 ng/ml EGF and 40 ng/ml HRG either added separately (red, EGF alone; blue, HRG alone) or in combination (black). Mean and SD are presented for the experimental data based on multiple replicates; model predictions are shown for the 7 representative parameter sets with dark lines representing the best fit parameter set. **D–F**) Measurements and predictions are shown for the levels of EGFR (D), HER2 (E) and HER3 (F) phosphorylation in the cell interior. Note the change in the y-axes scales between panels A–C and D–F. Lines and markers are as in panels A–C. Receptor phosphorylation levels are reported in arbitrary units in all panels as normalized in the ELISA experiments.

**Figure 4:**
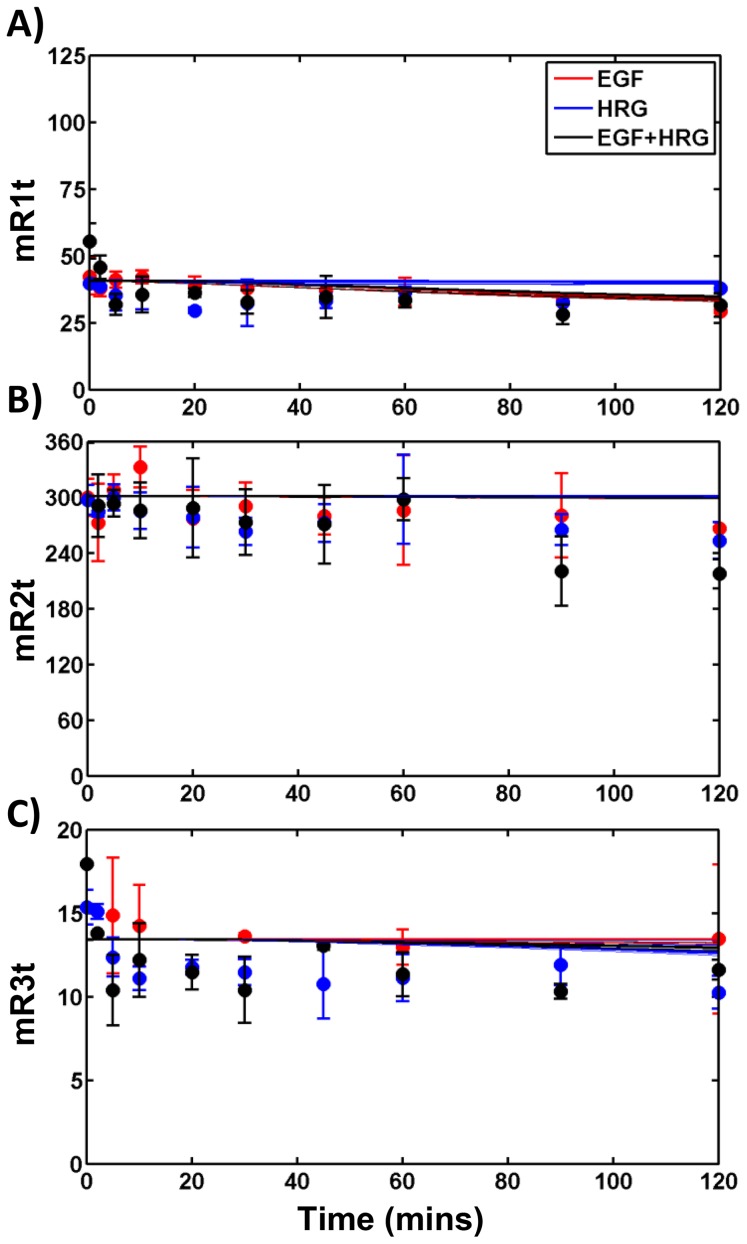
Total HER levels following ligand addition. Experimental data (markers) and model predictions (lines) for **A**) EGFR, **B**) HER2, and **C**) HER3 receptor mass as a function of time in the HER2+3+ cells following the addition of 12 ng/ml EGF (red), 40 ng/ml HRG (blue) or both (black). Line and marker descriptions are as in Figure 3. Receptor mass levels are reported in arbitrary units in all panels as normalized in the ELISA experiments.

In order to determine these unknown model parameters, we measured HER1-3 activation dynamics in a panel of HME cell lines with relatively constant levels of HER1, and different levels of HER2 and HER3 [47]. The complete set of data collected for one of the four cell lines used in our study that expresses all 3 HER receptors at significant levels (HER2+3+; designated with clone tag *D20*) is presented in Figures 2–4. The data includes measurements of the total levels of phosphorylated HER1-3 in response to various doses of EGF (Figure 2A–C, markers), and various doses of HRG (Figure 2D–F). We also obtained detailed time course measurements of total levels of phosphorylated HER1-3 (Figure 3A–C), levels of phosphorylated HER1-3 in the cell interior (Figure 3D–F), and HER1-3 total protein levels (Figure 4) in response to a single specific dose each of EGF and HRG, either added separately or in combination. Corresponding datasets for the parental (HER2−3−) cell line that expresses very low levels of HER2 and HER3; a cell line that expresses HER2 but not HER3 (HER2+3−, designated *24H*); and a cell line that expresses HER3 but not HER2 (HER2−3+, designated *B5*) are presented in Figures S3, S4, S5, S6, S7, S8 of Text S1. Note that consistent y-axis scales are used in Figures 2–4 and Figures S3, S4, S5, S6, S7, S8 of Text S1 for each measurement type to enable comparison of total receptor phosphorylation, internal phosphorylation levels and receptor mass across the four cell lines. In all, the experimental data consisted of 999 distinct measurements, with N> = 2 for each measurement.

We estimated the 47 unknown model parameters by simultaneously fitting the model to all of the data described above. Distinct measurement types (receptor phosphorylation, receptor mass) were scaled appropriately to ensure that they contributed comparable amounts to the residual vector (Methods). We found that ∼188 of the optimization runs converged with an RMSE relatively close to the best overall RMSE (Figure S1 in Text S1). In order to better assess the location of the solutions in the 47-dimensional parameter space, we used *k*-means clustering to identify the existence of distinct solution clusters (Figure S2 in Text S1). Our analysis indicated that the solutions can be split into 7 distinct clusters (Figure S2A in Text S1). In order to examine the similarities and differences in the model's behavior for these solutions, for each cluster we selected the parameter set that yielded the best fit (smallest RMSE), and used these 7 solutions (Figure S2B–C in Text S1) for additional analysis.

In Figures 2–4 and Figures S3, S4, S5, S6, S7, S8 of Text S1 we compare the experimental data (markers) to model predictions (lines) generated using each of the 7 representative parameter sets. Predictions using the parameter set with the best overall RMSE are depicted using darker lines in these plots. As seen, the model predictions are in good agreement with the experimental data (compare lines vs. markers of same color). Further, predictions using the 7 distinct parameter sets are in good agreement with each other (compare lines of the same color). This can also be seen in Figure S9 of Text S1 where the mean and standard deviations of the 7 distinct model predictions are plotted against the experimental data for the various types of measurements. There is a strong linear relationship between model predictions and the experimental data with a slope between 0.83 and 1.1 for most measurement types (Figure S9 in Text S1). The exceptions are for the levels of phosphorylated HER2 in the cell interior (slope = 0.64), and the EGFR receptor mass (slope = 0.77). Overall, model predictions are in good agreement with the experimental data with each of the 7 solutions yielding comparable results.

### Estimates for various model parameters

Parameter values for the 7 representative solutions are presented in Table S4 of Text S1. Although these solutions result in comparable fits to the various measurements (see Figures 2–4) and have similar overall RMSEs (Table S4 in Text S1), they involve substantial differences in the values of several parameters, with 20 parameters displaying a greater than 2-orders of magnitude variability. Reliability of the estimated parameters can depend on the information content of the training data, which in turn depends on the experimental design because the choice of which conditions are changed in the experiments can favor the identifiability of certain parameters. In other words, certain parameters would be more sensitive to the changed experimental conditions and this would allow for their better determination. For this reason, while some of the parameters can be extracted from the datasets reliably, we can only determine broad ranges for the rest of the parameters. The results indicate that both the dimerization affinities and the *pf* values are estimated with reasonable confidence for the R11 homodimer and the R12 heterodimer (in our notation Rij refers to the HERi-HERj dimer). However, there is more than two orders of magnitude variability in the dimerization and phosphorylation parameters related to the R13, R23, R22, R33 dimers (Table S4 in Text S1). Model predictions for the abundances of various receptor dimers generated using the 7 representative solutions reinforce these findings: predictions for R11 and R12 abundances fall within a narrow range, while there is considerable uncertainty in the estimates for the other dimers (Figure S10 in Text S1).

Visualization of the correlation between the parameters calculated based on the 188 solutions with good RMSE values (Figure S11 in Text S1) revealed that the dimer dissociation rates and phosphorylation efficiencies (*pf* values) were strongly correlated with each other for the various dimer types (Figure S11 in Text S1). This is because the extent of receptor phosphorylation for each of the three HER receptors is determined by both the absolute number of dimers of each type, as well as the *pf* values for the dimers (e.g., see equation in Figure 1B). Thus, estimating both the dimerization affinities and *pf* values by fitting the model to receptor phosphorylation data is expected to be challenging. The ability to overcome this limitation in the case of the R11 and R12 dimers is likely to be related to the availability of dose response datasets with strong HER1 and HER2 receptor phosphorylation in cell lines that express EGFR/HER1 alone, or HER1 and HER2, but not HER3.

### Model validation with receptor blocking experiments

**Figure 5:**
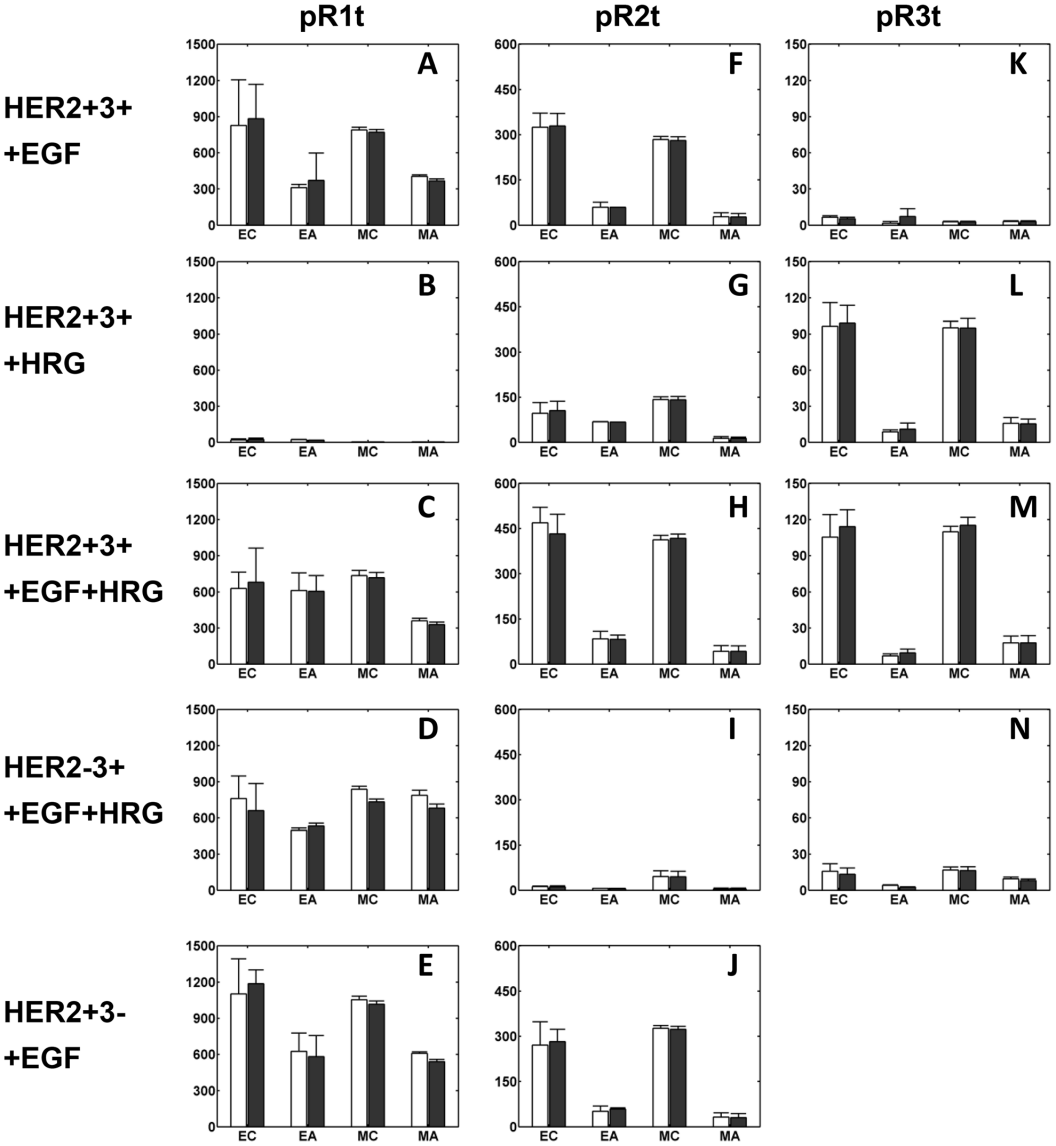
Inhibition of HER phosphorylation by 2C4. Experimental data and model predictions are shown for total **A–E**) HER1, **F–J**) HER2, and **K–N**) HER3 phosphorylation at 10 min (open bars) and 30 min (shaded bars) following ligand addition in the absence and presence of 2C4. Results are presented for various HME cells and ligand combinations as indicated to the left of each row in the figure. For example, panels A, F and K each involve the addition of EGF to the HER2+3+ cell line. EGF and HRG concentrations were 12 ng/ml and 40 ng/ml, respectively. The x-axis labels in panels are: “EC”, *experimental control* measurement (in the absence of 2C4); “EA”, *experimental antibody* measurement obtained with 2C4 addition; “MC”, *model control* prediction; and “MA”, *model antibody* prediction. The latter predictions were obtained by assuming that the antibody sequesters 95% of the cellular HER2 from the pool that is available for dimerization and receptor activation. Receptor phosphorylation levels are reported in arbitrary units in all panels as normalized in the ELISA experiments.

In order to independently validate the model, we collected additional data for receptor phosphorylation in various cell lines in the absence and presence of 2C4 (Pertuzumab) (Figure 5). This monoclonal antibody is considered to be a general inhibitor of HER2 dimerization due to its ability to bind the HER dimerization surface [51], [52], [53]. Model simulations for the antibody blocking experiments were performed by assuming that the addition of 2C4 renders 95% of the cellular HER2 unavailable for receptor dimerization. Note that the concentration of 10 µg/ml 2C4 used in our experiments is much higher than the K_d_ value for 2C4-HER2 binding [54]. Although none of the data in Figure 5 was used in “training” the mathematical model, the model does an excellent job of predicting these results. When model predictions for EGFR, HER2 and HER3 phosphorylation are plotted against experimental data from the validation experiments we obtain linear relationships with slopes of 0.92, 0.96 and 0.97, respectively (Figure S12 in Text S1). As before, model predictions of receptor phosphorylation based on the 7 distinct parameter sets are in excellent agreement with each other (see standard deviations of model predictions in Figure 5 and Figure S12 in Text S1).

### Dimer contributions to receptor phosphorylation in HME cells

HER receptors display unique patterns of site-specific phosphorylation, adaptor protein recruitment, and downstream signaling depending upon their dimerization partners [39], [40]. Therefore, it is of interest to quantify the relative contributions of various dimer types to HER phosphorylation. We calculated the phosphorylated levels of HER1-3 emanating from various dimers by multiplying the dimer abundances with appropriate phosphorylation efficiencies (see Methods). We found that although dimer abundances could be uniquely determined for only a subset of dimers (Figure S10 in Text S1), the contributions of various dimers to HER phosphorylation could be determined with much higher confidence (Figure S13 in Text S1). Predictions for the time-dependent phosphorylation signal from the various dimer types in the HER2+3+ cells using the 7 distinct parameter sets were in good agreement with each other (Figure S13 in Text S1). Since, the phosphorylation levels were found to be relatively stable beyond 1 hour of ligand addition (Figure S13 in Text S1), we chose the t = 60 min time point for all subsequent analysis. Model predictions for dimer contributions to HER1, HER2, and HER3 phosphorylation in the HER2+3+ cells at 60 min following the addition of saturating levels of EGF and HRG are presented in Figures S14, S15, and S16, respectively in Text S1. Predictions from the 7 different solutions, in general, were in good agreement with each other. The exception was for HER2 phosphorylation where the R12 dimer was found to contribute between 48–69% of the HER2 phosphorylation signal, with a 4–5% contribution from the R22 homodimer and the rest from the R23 dimer (Figure S15 in Text S1). Since, the predictions overall were in reasonable agreement we picked the best fit parameter set – the one with the lowest RMSE (Table S4 in Text S1) – and used it to generate predictions for the other cell lines in our panel.

**Figure 6:**
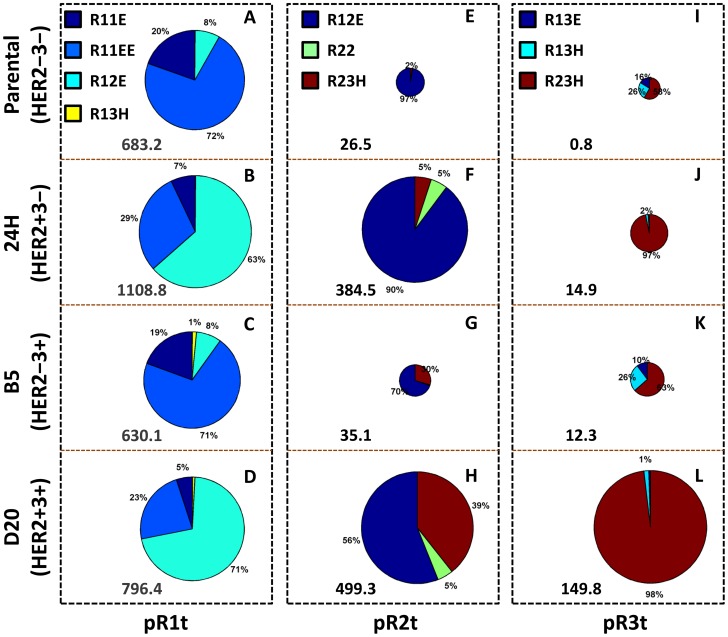
Model predictions for dimer contributions to HER phosphorylation in HME cells. The relative contributions of the various relevant dimers to **A–D**) HER1, **E–H**) HER2 and **I–L**) HER3 phosphorylation are presented as pie charts. The colors used for each dimer type are indicated in the top of each column. Each row corresponds to a different cell line, which is shown on the left margin. Circle sizes for any given receptor type (a given column) are scaled to indicate the relative levels of phosphorylation in the four cell lines. The total phosphorylation level is also indicated in numbers at the bottom of each pie chart. All predictions are for t = 60 min following the combined addition of 30 ng/ml EGF and 100 ng/ml HRG.

To understand the effect of HER expression levels on the phosphorylation pattern, we calculated the relative contributions of the dimers to HER activation in each of the 4 cell lines used in our study (Figure 6). In the figure, relative dimer contributions are shown as pie charts where the size of the circles is proportional to the total phosphorylation level (Figure 6). HER1 phosphorylation was found to be consistently high in all four cell lines with the highest level in the HER2+3− cells (Figure 6A–D). As expected, ∼90% of HER1 phosphorylation in the parental (HER2−3−) cells was found to be due to the R11 homodimer, with most of this contribution coming from the species where both dimer partners were ligand-bound (R11EE; Figure 6A). In the HER2+3− (24H clone) cells, >60% of EGFR phosphorylation was from the R12 dimer (Figure 6C). Dimer contributions to EGFR phosphorylation in the HER2−3+ (B5 clone) and HER2+3+ (D20 clone) cells were similar to that in the parental and 24H cells, respectively. In other words, HER1 and HER2 expression levels were found to dictate the HER1 phosphorylation pattern, with HER3 expression having little to no effect.

As expected, HER2 phosphorylation levels were predicted to be much higher in the HER2+3− and HER2+3+ cell lines compared to the HER2− cell lines (Figure 6E–H). The HER2+3+ cells were found to have the highest HER2 phosphorylation. Whereas in the HER2+3− cells 90% of HER2 phosphorylation was due to the R12 dimer (Figure 6F), there were substantial contributions from both the R12 and R23 dimers in the HER2+3+ cells (Figure 6H). Since the EGFR expression level in the HER2+3+ cells is an order of magnitude higher than the HER3 level (Table S5 in Text S1), this suggests a stronger propensity to form activated R23 dimers compared to R12 dimers. In all cases, we found that the R22 homodimer contributed less than 5% to HER2 activation. Overall, HER2 phosphorylation can be driven via interactions with either EGFR or HER3 with the latter appearing to be the preferred dimer partner.

High levels of HER3 phosphorylation were found only in the HER2+3+ cell line (Figure 6I–L). The R13 dimers (EGF or HRG-bound) were found to contribute significantly to HER3 activation when HER2 levels were low (Figure 6I, 6K). In cell lines that expressed both EGFR and HER2, HER3 activation was found to be dominated by the R23 interaction, which points to the significance of this interaction for HER3 activation.

### HER dimerization and phosphorylation as a function of receptor expression levels

**Figure 7:**
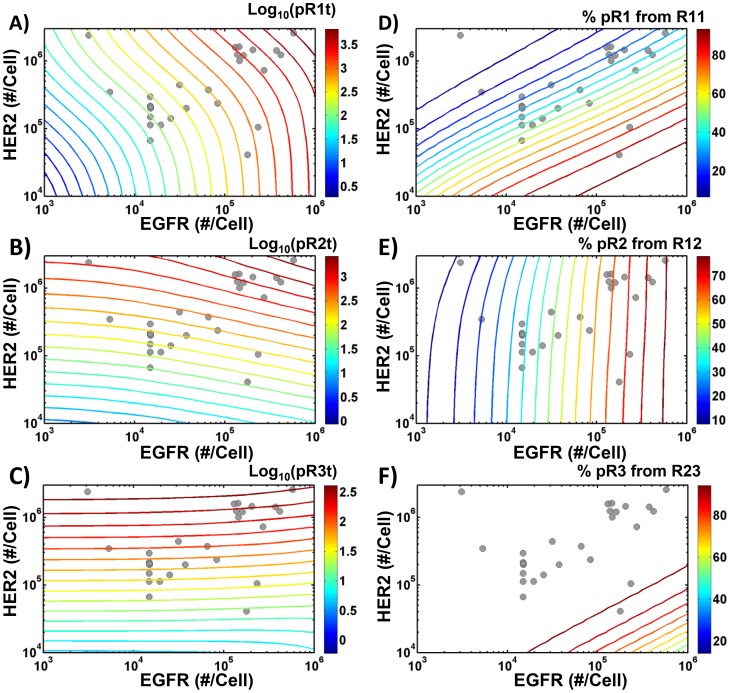
Effect of EGFR and HER2 expression levels on receptor phosphorylation and active dimer formation. **A–C**) Model predictions for the effect of EGFR and HER2 expression levels on EGFR (A), HER2 (B) and HER3 (C) phosphorylation. **D–F**) Effect of EGFR and HER2 expression levels on dimer contributions to receptor phosphorylation. Results are shown for the % contribution of the R11 homodimer to EGFR phosphorylation (D), of the R12 dimer to HER2 phosphorylation (E), and of the R23 dimer to HER3 phosphorylation (F). All predictions were generated for a fixed HER3 expression of 40,000 receptors/cell. This expression level was chosen based on a compilation of HER3 expression levels from the literature (see the text for more details). Results are shown for phosphorylation at t = 60 min following the combined addition of 30 ng/ml EGF and 100 ng/ml HRG. The grey dots in each panel indicate the HER1 and HER2 expression levels of various HER3-expressing cell lines.

Our model can be used to predict the extent of HER phosphorylation and the receptor dimerization pattern for any combination of HER1-3 expression levels. We generated model predictions over a wide range of receptor expression levels (Figure 7). To ensure the relevance of this analysis, we obtained information on the HER expression levels of various cell lines from the literature (Table S5 in Text S1). Since, most HER3-expressing cells typically display a receptor expression level of ∼40,000 molecules/cell (Figure S17 in Text S1), we fixed HER3 at this level. We varied EGFR from 10^3^ to 10^6^ and HER2 from 10^3^ to 3×10^6^ to encompass the receptor expression levels observed in various cell lines (Table S5 and Figure S18 in Text S1). HER1-3 phosphorylation levels (Figure 7A–C) and the percentage contribution from the R11 homodimer to EGFR phosphorylation (Fig 7E), the R12 dimer to HER2 phosphorylation (Figure 7F) and the R23 dimer to HER3 phosphorylation (Figure 7G) are presented in Figure 7 as a function of EGFR and HER2 expression levels. The contribution of the other dimer types to HER1-3 phosphorylation is presented in Figure S19 in Text S1. Simulation results indicate that EGFR phosphorylation increases with both EGFR and HER2 expression, with HER2 expression having a stronger effect at low to moderate EGFR expression (Figure 7A). The EGFR homodimer contributes anywhere from 0–100% of the EGFR phosphorylation signal with the actual contribution increasing with EGFR expression and decreasing with HER2 expression (Figure 7B). The contribution of the R12 dimer to HER1 phosphorylation displays the opposite pattern (Figure S19A in Text S1), while the R13 dimer contributes <10% to HER1 phosphorylation in all cases (Figure S19B in Text S1).

HER2 phosphorylation increases with HER2 expression, with EGFR expression having only a minor effect (Figure 7B). The contribution of the R12 dimer to HER2 phosphorylation shows a broad range, increasing with EGFR expression (Figure 7G). The contribution of the R23 dimer shows the opposite pattern (Figure S19C in Text S1). The HER2 homodimer is predicted to contribute <15% of the HER2 phosphorylation signal in all cases (Figure S19D in Text S1). Interestingly neither the R12 contribution nor the R23 contribution is a strong function of HER2 expression (Figure 7G, Figure S19C in Text S1). Thus, the EGFR expression level is the strongest predictor of which dimer type dominates HER2 signaling.

HER3 phosphorylation is found to be strongly dependent on HER2, but not EGFR expression levels (Figure 7C). Over the range of expression levels seen in actual cells (see dots in Figure 7D), >80% of the HER3 signal is predicted to be due to the R23 dimer (Figure 7D) with the remaining from the R13 dimer (Figure S19E in Text S1).

In order to validate these simulation results, we compared model predictions for HER3 activation in two distinct cell lines – ADRr and ADRrE2 – with similar levels of EGFR and HER3, but distinct HER2 levels with previously published experimental data [27]. As seen, model predictions for the relative change in HER3 activation due to an increase in HER2 expression are in good agreement with the experimental data (Figure 7). Thus, our results indicated that the model predictions may be applicable to other cell lines as well. To enable cell type-specific comparisons, we have computed the phosphorylation levels of HER receptors and HER dimerization patterns for 52 distinct cell lines using their receptor expression levels compiled from the literature (Figures S20, S21, S22 in Text S1).

### Dependence of Erk and Akt activations on HER phosphorylation

One key aspect of receptor signaling is the prediction of how the changes in receptor phosphorylation levels would alter the activation patterns of the downstream elements of the involved signaling pathways. Here, we briefly illustrate how the constructed receptor activation model can be used to quantitatively predict the relative contributions of the HER receptor types and their dimers to the activations of Erk and Akt kinases. Erk and Akt are important regulators of the cell proliferation and mobility processes, and their activation kinetics in HME cells were subject of our earlier investigations [47], [55].

We pursued multilinear regression analysis to determine the relationship between Erk and Akt activation and HER phosphorylation by fitting the coefficients of the regression model to the data collected in our HME cell lines. This analysis was pursued in two different ways by assuming a) that the total receptor phosphorylations are the predictors, i.e., pT(t) = b0+Σ b_i_ * pR_i_(t), where pR_i_(t) is the contribution of receptor type i ( = HER1, 2, or 3) to the activation of the target protein T ( = Erk or Akt) and the sum is over the receptor types, and b) that the receptor dimer contributions are the predictors [55], i.e., pT(t) = b0+Σ b_i_ji_ * pR_i_ji_(t), where pR_i_ji_(t) is the contribution of the dimer Rij to the phosphorylation of receptor type i and the sum is over the receptor dimer types. Comparison of the regression model predictions with the experimental data have shown that the EGFR/HER1 contribution to Erk phosphorylation (pERK) is the dominant predictor and that various receptor dimers could make comparable contributions to the prediction of pERK (Table S1; Figure S24 in Text S1). In contrast to Erk, Akt phosphorylation has a much stronger dependence to the activation through particular receptor dimers: regression analysis indicated that signaling through the HER1-HER3 receptor dimer was the dominant predictor of pAKT (Table S1; Figure S25 in Text S1). Results of this analysis were consistent with the results of other analysis methods such as clustering and targeted inhibition (Gong et al, in preparation).

## Discussion

We have constructed a parsimonious mathematical model for HER1-3 activation that incorporates the important biochemical/biophysical steps involved in the process, and have parameterized it using the data collected in a panel of HME cells that express varying levels of HER1-3. Despite using rate constants from the literature where available, and considering an extensive dataset including total and internal receptor phosphorylation levels and receptor mass measurements as a function of ligand dose, our analysis indicates that not all aspects of the model are equally identifiable. Specifically, we find that while there is considerable uncertainty surrounding the absolute dimer abundances of all but the HER1 homo- and HER1-HER2 hetero-dimer types (Figure S10 in Text S1), the phosphorylation signal from all the dimer types can be predicted with good confidence using the model (Figure S13 in Text S1). Since the tyrosine phosphorylation levels in various dimers are the relevant quantities to consider in the context of signal transduction, the obtained results provide the needed information for probing dimer specific downstream responses. That said the lack of complete model identifiability still highlights the challenges encountered in the construction and parameterization of models for biomolecular networks. Additionally, as in almost all of the earlier studies, possible location-dependence of the HER receptor kinetics was not included in our study. Receptor placement in membrane ruffles or the corralling role of the cytoskeleton elements [56] could be important factors but such complexities cannot be captured with the design of our experiments and hence were omitted.

Previous modeling studies of the co-expression of multiple HER receptors considered both receptor activation and downstream signaling (Erk and Akt activation) as part of an integrated analysis [43], [45]. They involved the use of a single cell type [45], or multiple distinct cell types [43]. These models are useful because they represent comprehensive quantitative frameworks for assembling information regarding HER-mediated signaling, and serve to document the various steps in the process. They have also been utilized in subsequent studies that have focused on therapeutic targets for HER3-mediated signaling [27], [28]. However, due to the large scope of these models, model identifiability is a challenge as noted by the authors themselves [27], [43]. Here, we adopted an alternate approach to establish the quantitative link between HER expression levels and downstream signaling: we constructed a relatively detailed mechanistic model for HER activation, since the available information and datasets allowed us to do so. As a next step, we have used the dimer phosphorylation levels predicted by the model (an aspect that is identifiable given the data), along with data on the activation of MAPK and Akt signaling pathways to quantify differential signaling by the various HER dimers (Supplementary Material). We and others have previously used this conceptual step-wise approach to analyze HER-mediated signaling in cells that co-express EGFR and HER2 [55], [57], [58], [59]. Interestingly, as briefly discussed in the Results section, our recent analysis also indicated that activation of the pro-survival Akt pathway correlated more with HER3 signaling from the smaller R13 dimer pool compared to the substantially larger signal from the R23 dimers (to be submitted).

The current model can predict the abundance of R11 and R12 dimers with much higher confidence than that of the other dimer types (Figure S10 in Text S1). The predictions for dimer abundance, and the associated parameters for these two dimer types, can be compared with previous results. We recently used a simpler model for the activation of a single receptor type (EGFR) to analyze the data for the parental HME cell line, and showed that even under saturating concentrations of EGF, <40% of the receptors dimerize and are phosphorylated [49]. These findings were in agreement with previous experimental data from our laboratory where we quantified the fraction of phosphorylated EGFR [59]. While our previous model [49] neglected the presence of low levels of HER2 and HER3 in the parental cell line, our current analysis explicitly accounts for this aspect (Table S5 in Text S1). Further, our current analysis involves the simultaneous optimization of the model using data from four different HME cell lines. Despite these differences, the current model also predicts that for the parental cell line <40% of the EGFR form homodimers following ligand stimulation, with much lower abundances for the other dimer types (Figure S23 in Text S1). The value of the phosphorylation efficiency factor *pf* for the R11 dimer with two bound EGF molecules estimated here (see the pf11ees values in Table S4 of Text S1) ranges from 2.8×10^−2^ to 3.4×10^−2^, which is in excellent agreement with the mean value of 2.97×10^−2^ estimated in our previous manuscript [49]. This suggests that the four HME cell lines behave in a consistent manner, since the simultaneous analysis of these cell lines yields findings that are consistent with the analysis of the parental cell line in isolation.

We have previously used a much simpler model for HER activation that neglected explicit consideration of receptor-ligand binding, receptor recycling, and sorting to analyze receptor activation in cells co-expressing EGFR and HER2 alone [59]. In that analysis we assumed that the formation of active dimers occurred in a single lumped step, and for each dimer type we used a single lumped *pf* value applicable to all cellular compartments [59]. The analysis indicated decreased stability of the R12 dimer compared to the R11 dimer, which contradicted the assumptions used in other modeling papers [57], [60]. Further, we found that the pf11 and pf12 values were comparable indicating that EGFR phosphorylation occurred with equal efficiency in R11 and R12 dimers, and that pf22 was an order of magnitude smaller than pf21 indicating much lower HER2 phosphorylation efficiency in the R22 homodimer compared to the R12 dimer [59]. Comparison of the dimer dissociation constants obtained in our current analysis (see ku11ees and ku12es in Table S4 of Text S1) also indicates that the R11 dimer is far more stable than the R12 dimer. However, the model predicts that EGFR phosphorylation is 6–35 times more efficient at the cell surface and 1.5–4 times more efficient in the early endosome vesicles when the EGFR is part of the R12 dimer as opposed to the R11 dimer (see pf11ees/pf12es and pf11eei/pf12ei ratios in Table S4 of Text S1). The model, in agreement with our previous findings [59], also predicts that the HER2 phosphorylation is far more efficient in the R12 dimer compared to that in the R22 homodimer (see pf21s/pf22s ratio in Table S4 of Text S1). One way of validating these results would be to measure the absolute abundances of R11 and R12 dimers in these cell lines. While it is possible to address these using FRET or co-IP experiments, these experiments would be challenging due to the difficulties in quantitative interpretation of the FRET signal, and possible differences in antibody pull down efficiencies, respectively.

We used the model trained on data from HME cells to predict the HER activation levels and dimer contributions for a range of cell lines (Figure 7; Figures S20, S21, S22 in Text S1). For these predictions we first had to obtain information on receptor expression levels in various epithelial cell lines (see Table S5 in Text S1). Interestingly, while the data revealed wide variability in the expression levels of EGFR and HER2 among the cell lines, cells that expressed HER3 did so in a relatively narrow range of ∼30,000 to 60,000 receptors/cell (Table S5 in Text S1). Perhaps, this indicates that the *in vivo* quantitative regulation of HER3 signaling occurs via control of the expression level of its main partner HER2 and/or via the differential regulation of its ligands [61].

**Figure 8:**
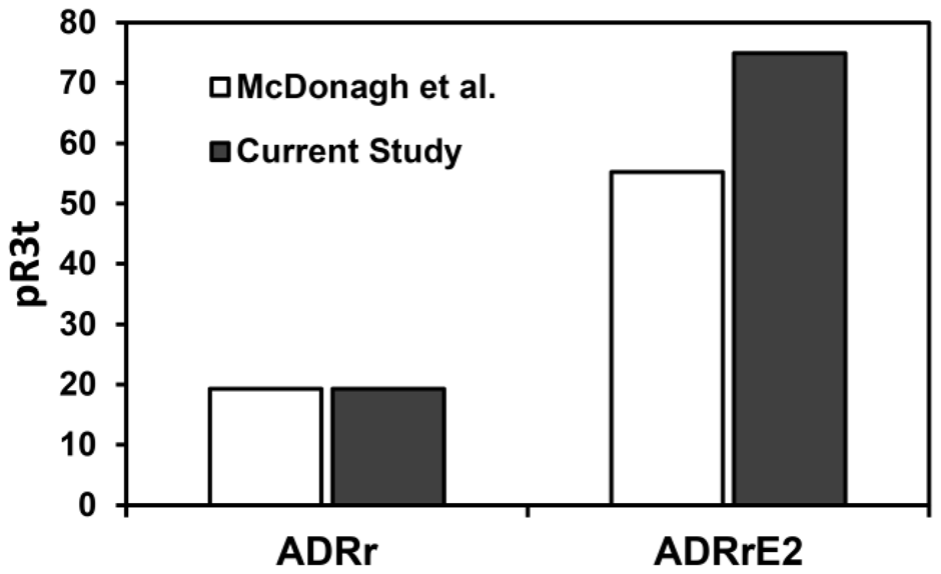
The effect of HER2 expression levels on HER3 phosphorylation. Experimental data (open bars) and model predictions (shaded bars) for the HER3 phosphorylation level (in arbitrary units) at t = 10 min following the addition of 5 ng/ml HRG in two cell lines that respectively express low (ADRr) and high levels of HER2 (ADRrE2). The experimental data (from McDonagh et al., [27]) have been normalized to obtain the same phosphorylation level in the ADRr cell line as in the model prediction.

We partially validated the ability of our model to predict HER phosphorylation dynamics in other cell lines by comparing our results with experimental data [27] for HER3 phosphorylation in the ADRr and ADRrE2 cell lines (Figure 8). In such extrapolations we make the implicit assumption that the rate constants for HER activation processes (receptor-ligand binding, dimerization, phosphorylation, trafficking) are similar across various cell lines, and that knowledge of receptor expression levels alone is sufficient to predict dimerization and phosphorylation patterns. We caution that the validity of the assumption may be questionable, and that extrapolations to other cell lines should be specifically validated (for e.g., by measuring the levels of HER2 phosphorylation relative to a benchmark cell line) when quantitative predictions of dimerization patterns are desired. That said our predictions appear to be in qualitative agreement with published results. For instance, we predict that the R12 dimer is an important component of EGFR and HER2 activation with the contribution of this dimer dependent on both EGFR and HER2 expression levels. This is in agreement with the finding of Defazio-Eli et al. who used the VeraTag™ (Monogram Biosciences, South San Francisco, CA) proximity-based ligation assay to quantify EGFR and HER2 expression levels, R12 dimer abundances and phospho-R12 levels in various cells [62]. Mukherjee et al. [63] also used VeraTag assays to quantify the phosphorylation levels of various HER receptors and relative dimer abundances in panel of breast tumors with particular focus on HER3 activation. They found that the level of HER3 phosphorylation correlated strongly with the level of the R23 dimer, and that the expression level of HER2 is a strong determinant of the level of HER3-PI3K signaling [63]. This is in agreement with our finding that the HER2 expression level is the strongest determinant of HER3phosphorylation (Figure 7C), and that it is the R23 dimer that contributes significantly to HER3 activation (Figure 7F).

To summarize, we have constructed and parameterized a mathematical model that can be used to predict the levels of HER phosphorylation, and the levels of various phosphorylated HER dimers as a function of the HER expression profile. We present predictions of HER1-3 phosphorylation levels and their dimerization patterns for 52 distinct cell lines (Figures S20, S21, S22 in Text S1). These results can be used to determine the dominant dimer type that contributes to HER signaling in each cell line, and hence to device optimal strategies to disrupt HER signaling in a cell lines with known HER expression levels. Importantly, model predictions can be used to determine the relative potencies of the various HER dimers to activate distinct downstream cell signaling pathways, and drive specific cell decisions. In this regard, this manuscript represents a critical piece in the effort to mechanistically link HER expression levels to receptor dimerization, activation, and eventually to the cell phenotype.

## Methods

### Cell culture and treatment

The *parental* human mammary epithelial (HME) cell line used in this study was originally provided by Martha Stampfer (Lawrence Berkeley National Laboratory, Berkeley, CA) as cell line 184A1-1. It expresses approximately 200,000 molecules of EGFR/HER1, and much lower levels of HER2 and HER3 [47], [57], and is designated here as the HER2−3− cell line. We used retroviral transduction to insert the HER2 gene and the HER3 gene into the parental cell line to obtain the 24H (HER2+3−) and B5 (HER2−3+) cell lines, respectively. The HER3 gene was then inserted into the 24H cell line to obtain the D20 cell line (HER2+3+) that expressed all 3 receptors. We have previously described the detailed protocols used for deriving these cell lines [47]. The parental cell were maintained in DFCI-1 medium supplemented with 12.5 ng/ml EGF (PeproTech, Rocky Hill, NJ) as described previously [64]. Growth mediums for the 24H cell line, the B5 cell line, and the D20 cell line were the same as the parental cell line except for the addition of antibiotics G418 (250 µg/ml; Invitrogen, Carlsbad, CA), puromycin (2 µg/ml; Sigma, St. Louis, MO), and both, respectively to ensure selection [47].

When cells grew to near confluency, DFCI-1 medium was replaced with bicarbonate-free DFHB minimal medium lacking all supplements but 0.1% bovine serum albumin. Cells were then brought to quiescence for 12–18 hours before treatment. Cells were activated through the HER receptors by the addition of known concentration of EGF and/or HRG (Peprotech, Rocky Hill, NJ) followed by incubation at 37°C for fixed amounts of time from 5 to 120 min. In the dimerization blocking experiments, cells were preincubated with 10 µg/ml of monoclonal antibody 2C4 (Pertuzumab; generous gift from Genentech, Inc, San Francisco, CA) for 4 hours prior to ligand stimulation. Following stimulation, cells were then solubilized with ice cold lysis buffer (1% NP-40, 20 mM pH 8.0 Tris buffer, 137 mM NaCl, 10% glycerol, 2 mM EDTA, supplemented with 1 mM heat activated sodium orthovanadate and 1% protease inhibitor cocktail III; Calbiochem, La Jolla, CA) for 20 min. Cell lysates were collected with a scraper. Lysates were centrifuged at 13,000 rpm for 10 min at 4°C, and the supernatants were transferred into fresh microtubes. Obtained cell lysates were either analyzed immediately or stored at a −80°C freezer until needed.

Phosphorylated receptor levels in the internal compartments were determined using an acid-stripping protocol, which selectively dephosphorylates cell surface receptors without altering the phosphorylation of internalized receptors [65]. Following cell stimulation with ligands, and acid stripping, cells were washed 3X with ice cold PBS and incubated at room temperature for one minute to allow surface receptor dephosphorylation. After another round of cold PBS washing, cells were solubilized and lysates were prepared as described in the previous paragraph.

### Receptor mass and phosphorylation measurements

ELISA assays to quantify the receptor mass and phosphorylation levels were performed using the R&D DuoSet IC ELISA kits (R&D Systems Inc., Minneapolis, MN). Two types of ELISA data were collected as a function of time following ligand addition for each of the four cell lines used in our study:

1. *HER receptor masses* (mRt) were quantified in total cell lysates using capture and probe antibodies specific to each HER receptor following the addition of 12 ng/ml EGF and/or 40 ng/ml HRG. These experiments involved detailed time course measurements over 2 hours to assess the kinetics of receptor degradation. The EGFR, HER2 and HER3 receptor masses are designated as mR1t, mR2t and mR3t, respectively.
2. *The total extent of HER tyrosine phosphorylation* (pRt) was assayed by pulling total cell lysates down with antibodies specific to each HER receptor, and subsequently probing with a polyclonal phospho-tyrosine antibody. These measurements were done at a few selected time points (0, 10, 30, 60 min) for various concentrations of EGF from 0.6 to 30 ng/ml, and various concentrations of HRG from 4 to 120 ng/ml, with the ligands being added individually. We also assessed the effect of adding the ligands together in experiments where pRt was measured as a detailed time course following the addition of 12 ng/ml EGF and/or 40 ng/ml HRG. The total receptor phosphorylation level for EGFR, HER2 and HER3 receptors are designated as pR1t, pR2t and pR3t, respectively.
3. The *extent of HER phosphorylation in the internal compartments* (pRi) was quantified by measuring receptor phosphorylation following acid stripping. Internal phosphorylation measurements were obtained as a detailed time course following the addition of 12 ng/ml EGF and/or 40 ng/ml HRG. Internal phosphorylation levels for EGFR, HER2 and HER3 receptors are designated as pR1i, pR2i and pR3i, respectively.

The ELISA results were normalized based on the total protein present in the cell lysate (measured using the Bicinchoninic Acid protein quantitation kit, Sigma, St. Louis, MO), and were expressed in units of picograms per microgram of total lysate protein. For each cell line and treatment condition at least two independent measurements were performed, with at least two biological replicates in each experiment.

### Mathematical model for HER activation

The mathematical model for HER activation (Figure 1) is an extension of our recently published multi-compartment model for cells expressing EGFR alone [49]. Here, we consider the interactions between multiple members of the HER family. There are 17 types of species in the mathematical model (Figure 1A) including the ligands EGF and HRG, free and ligand-bound HER monomers, and the various possible homo- and hetero-dimers that can be formed following the addition of EGF and/or HRG. These species are allowed to exist in 3 distinct compartments – the cell surface, early endosomes (EE) and late endosomes (LE) – resulting in a total of 51 model variables. The model combines the key biochemical reactions underlying HER activation (Figure 1B) with receptor trafficking between the cellular compartments (Figure 1C) to predict receptor mass, dimerization and phosphorylation dynamics following ligand stimulation.

In the model biochemical reactions leading to receptor activation – receptor-ligand binding, dimerization and phosphorylation – are allowed to occur at the cell surface and in the EE. Following exit from the EE, receptors destined for degradation become part of multivesicular bodies (MVBs) where they undergo terminal dephosphorylation prior to degradation [66], [67]. To account for this process, we include an idealized LE compartment which is a site for the accumulation of dephosphorylated receptors prior to degradation [49]. We assume that receptors in the LE do not contribute to receptor phosphorylation measurements, but contribute to receptor mass measurements. Since it is unnecessary to track the receptor activation process in the LE, biochemical reactions for this compartment were excluded from the rate equations.

Our general approach is to construct a parsimonious model to avoid over-fitting of the data. We use lumped parameters or scaling factors where detailed kinetic information is unavailable. To ground the model in reality, and to facilitate parameter estimation, we employ previously determined values for rate constants where available. Explicit consideration of the various HER homo- and hetero-dimer types in their different ligand-bound states demands the specification of a large number of model parameters due to the combinatorial complexity. However, we choose this approach because quantitative information is available in the literature regarding the relative affinities of various HER dimer types for EGF and HRG [68], [69], [70]. Further, we can use reasonable simplifying assumptions regarding the trafficking properties of the different species to reduce the number of unknown parameters in the model (see below).

The different reaction types in the model are briefly discussed below along with their associated assumptions, known parameter values, and unknowns. The complete governing equations for the model are presented as part of the Supporting Information. Rate expressions and parameter values used for the biochemical reactions at the cell surface and the EE are in Tables S1 and S2, respectively of Text S1. The trafficking parameters are presented in Table S3 of Text S1. Estimates for unknown model parameters obtained here by fitting to the experimental data are tabulated in Table S4 of Text S1.

#### Receptor-ligand binding

We model the reversible binding of EGF to EGFR, and HRG to HER3 using mass action kinetics. On and off rates for the EGF-EGFR interaction at the cell surface and in the low pH environment of the EE are available in the literature [57], [68], [71]. Rate constants for the HRG-HER3 interaction at the cell surface are also available [68]. Due to the lack of information, we assume that the on and off rates for this reaction in the EE are the same as those for the EGF-EGFR interaction in that compartment [44]. In general we assume identical receptor-ligand binding kinetics irrespective of whether the receptor is a monomer or is part of a dimer. The exceptions to this rule are as follows: i) the presence of HRG is assumed to decrease the EGF binding affinity of the HER1-HER3 dimer by a factor of 3 [69], ii) the HER1-HER2 dimer is assumed to have a ∼60% stronger affinity for EGF compared to HER1 alone [68]; the stronger affinity is also supported by a recent study of ligand binding to cells co-expressing HER1 and HER2 [70] iii) the HER2-HER3 dimer is assumed to have a 25-fold stronger affinity for HRG compared to HER3 alone [68]. Overall, all the receptor-ligand binding parameters are treated as known quantities in our analysis (parameter values in Tables S1 and S2 of Text S1).

#### Receptor dimerization

We assume that ligand-bound EGFR and HER3 molecules, and the HER2 receptor, which has no ligand, can reversibly form homo- and hetero-dimers according to mass action kinetics. Although pre-formed receptor dimers involving EGFR and HER3 may exist in the absence of ligand addition, we assume that such basal dimerization occurs at a low level and contributes insignificant amounts to receptor activation. This is in line with the idea that ligand-induced conformational changes greatly enhance dimer stability and kinase activity of the HER receptors [29], [31]. We assume that the dissociation of ligand from a dimer containing a single ligand molecule results in an instantaneous dissociation of the dimer (see Tables S1 and S2 in Text S1). This eliminates the need to track the unstable ligand-free dimers that would form in such a scenario. We assume that receptor dimerization is a diffusion-limited process [72], [73], and employ the same forward rate constant for all dimerization reactions (Tables S1 and S2 in Text S1). The reverse rates for the 10 distinct dimer types at the cell surface and the EE are treated as unknowns (Table S1, S2 in Text S1). These 20 parameters are estimated as part of the model optimization (estimated values are reported in Table S4 of Text S1).

#### Receptor phosphorylation

Dimerization results in *trans*-phosphorylation of the receptor cytoplasmic tails at multiple tyrosine sites. The dimerization and phosphorylation reactions are expected to be rapid relative to the time scale of experimental sampling, preventing reliable parameterization of the individual steps of these consecutive reactions. Hence, we treat receptor phosphorylation/activation as a lumped process. We explicitly track the dimers, while the contribution of the dimers to the phosphorylation signal is accounted for with dimer-specific multiplicative phosphorylation factors (*pf*), which are used to convert dimer abundances to the phosphorylation signal measured in the ELISA experiments [49]. The *pf* is a lumped factor that accounts for the characteristics of all possible tyrosine sites in a dimer. It captures the relative potency of various dimer types in contributing to receptor phosphorylation. Here we assume that the *pf* values are time-invariant biophysical constants that do not depend upon the ligand concentration since they capture molecular scale processes within an already formed dimer. For example, the contributions of the EGF and HRG bound HER1-HER3 dimer to the EGFR and HER3 phosphorylation signals at the cell surface are respectively expressed as [R13EHs]*pf13ehs and [R13EHs]*pf31ehs where [R13EHs] is the dimer abundance and pf13ehs and pf31ehs are cell surface phosphorylation factors. In our notation specific pf values are written as pf<ijc> where i and j specify the HER types in the dimer; i is the receptor whose phosphorylation level is being computed; c is the cell compartment. An illustrative expression for calculating the total EGFR phosphorylation signal at the surface using the various relevant *pf* values is shown in Figure 1B. Similar expressions for calculating the other relevant phosphorylation levels are presented in Supporting Information. Here, since the HER3 receptor lacks kinase activity, we set the pf values for the HER3 homodimers to 0. In order to compute the HER1, HER2 and HER3 phosphorylation levels from the various dimer abundances, we need to specify a total of 13 *pf* values (pf11es, pf11ees, pf12es, pf13es, pf13hs, pf13ehs for pR1s; pf21es, pf22s, pf23hs for pR2s; pf32hs, pf31es, pf31hs, pf31ehs for pR3s) each at the cell surface and the EE. These 26 pf values are unknowns that are estimated as part of the optimization (Table S4 in Text S1).

#### Receptor trafficking

Internalization, recycling and degradation are treated as first-order processes with species-specific rate constants. The pinocytosis of free ligand molecules is ignored. The internalization rate (*kt* for monomer, *ke* for dimers), endosomal exit rate *kx*, and recycling fraction *f* have been previously determined for EGFR and HER2 species in HME cells ([57], [58]; see Table S3 in Text S1). For HER3-related parameters, we assume that the trafficking properties of HER3 are identical to that of HER2 [38]. In order to calculate the individual trafficking rates using the *kx* and *f* values, we define a parameter *δ* for each species as the ratio of the entry to exit rates for the LE (see [49]). For any given species *i* we can then write the recycling rate *kri*, the LE entry rate *kli*, and the degradation rate *kdi*, as *kri* = *kxi fi* (1+*δi*); *kli* = *kxi* (1−*fi*)(1+*δi*) and *kdi* = *kxi* (1−*fi*)(1+*δi*)/*δi* (see [49]). We assume that sorting occurs prior to incorporation in the LE, and that once in the LE, all molecules are degraded at the same rate. With this assumption, given *δ*1, the *δ* value for the EGFR monomer, the *δ* value for any species *i* can be calculated using the expression 1+1/*δi* = [*kx*1(1−*f*1)(1+1/*δ*1)]/[*kxi* (1−*fi*)] where *kx*1 and *f*1 are the reported endosomal exit rates and recycling fractions for the EGFR monomer [49]. Overall, given the known trafficking properties of the receptors (Table S4 in Text S1), we are left with a single unknown parameter, *δ*1, which is estimated as part of model optimization (Table S4 in Text S1).

### Parameter estimation

There are 47 unknown model parameters (described above), which include the dimer dissociation rates, the pf values for the various dimer types and the trafficking parameter *δ*1. Given values for the 47 parameters and the HER1-3 expression levels (Table S5 in Text S1), the model can be simulated for any given concentration of EGF and/or HRG to predict the total (pRt) and internal (pRi) receptor phosphorylation levels as well as the receptor mass (mRt) for the three HER receptor types. In order to estimate the unknown parameters, we simultaneously considered these 3 distinct measurement types for each of the 4 HME cells. We constructed scaled residual vectors (residual = model prediction−experimental data) for the pR and mR predictions by dividing each of these residuals by the maximum values measured in the phosphorylation and receptor mass measurements. This ensures roughly equal importance to the distinct measurement types during parameter estimation. We then concatenated the scaled residual vectors and used *lsqnonlin* – the MATLAB (Natick, MA) nonlinear least squares regression function to determine optimal parameter values.

During optimization, initial guesses for the unknown kinetic rate parameters were generated by sampling the parameters from broad uniform distributions: guesses for the dimer dissociation rates ranged from 10^−3^ to 10^3^; the pf values from 10^−6^ to 1 and *δ*1 from 0.1 to 10. To ensure convergence, we adopted a progressive optimization approach. HER1-related parameters were first estimated using the parental (HER2−3−) cell line. These values were then used as initial guesses in the estimation of parameters related to HER1-HER2 interactions using the parental and HER2+3− cell lines; and parameters related to HER1-HER3 interactions using the parental and HER2−3+ cell lines. The parameter sets obtained from these simpler optimizations were used as initial guesses in the final set of optimization runs where all 47 parameters were estimated by simultaneously considering the data from all four cell lines. These optimization runs were repeated 500 times with randomly generated initial guesses for parameters related to the HER2-HER3 interaction. We used the overall root-mean-squared error (RMSE) between the experimental data and model predictions to assess the goodness of the fit.

### Model predictions for HER dimerization and activation

Following parameter estimation, model predictions were generated for dimer abundances, the HER1-3 phosphorylation signal from various dimers (product of abundance and appropriate pf value), and the total HER1-3 phosphorylation levels (sum of the relevant dimer phosphorylation signals). These results were used to compute the fractional contribution of the various dimer types to the phosphorylation of the HER1-3 receptors. Predictions were generated both for HME cells as well as a panel of 48 cell lines for which HER expression levels were compiled from the literature (Table S5 in Text S1). Unless specified otherwise, all predictions represent the HER dimerization and activation pattern at t = 60 min following the addition of saturating doses of both ligands, specifically 30 ng/ml EGF and 100 ng/ml HRG.

## Supporting Information

Text S1 *Supplemental Methods* describing details of the modeling and analysis methodology, *Supplemental Tables* S1, S2, S3, S4, S5, and *Supplemental Figures* S1, S2, S3, S4, S5, S6, S7, S8, S9, S10, S11, S12, S13, S14, S15, S16, S17, S18, S19, S20, S21, S22, S23, S24, S25. Detailed figure and table legends are provided in the file.
(PDF)

Table S1 Multilinear regression analysis model parameters.
(XLS)
