# Supplementary material for: Model-Based Analysis of HER Activation in Cells Co-Expressing EGFR, HER2 and HER3
Source: PLoS Comput Biol. 2013 Aug 22;9(8):e1003201. doi: 10.1371/journal.pcbi.1003201 (PMC3749947; doi:10.1371/journal.pcbi.1003201)
Supplement: Text S1 — Supplemental Methods describing details of the modeling and analysis methodology, Supplemental Tables S1, S2, S3, S4, S5, and Supplemental Figures S1, S2, S3, S4, S5, S6, S7, S8, S9, S10, S11, S12, S13, S14, S15, S16, S17, S18, S19, S20, S21, S22, S23, S24, S25. Detailed figure and table legends are provided in the file. (PDF) [file pcbi.1003201.s001.pdf]

SUPPORTING INFORMATION TEXT S1 FOR

Model-based analysis of HER activation in cells co-expressing EGFR, HER2 and HER3

Harish Shankaran, Yi Zhang, Yunbing Tan, and Haluk Resat

SUPPLEMENTAL METHODS

1. Governing equations for the mathematical model

The equations describing the rates of change of the 51 distinct model variables (17 molecular species types in 3 distinct compartments) are provided below. In the following equations terms of the type "v<i>s" refer to the ith biochemical reaction flux at the cell surface; "v<i>e" refers to the flux for the ith reaction in the EE. How these fluxes depend on the model parameters, and the values used for these parameters are provided in Tables S1 (surface fluxes) and S2 (EE fluxes). Nav denotes Avogadro's number (6.022×10^{23}). Vmc refers to the volume of media per cell and is set equal to 5×10^{-10} liters/cell. Ve is the volume of the EE which is set to 3×10^{-14} liters [1]. The concentrations for EGF and HRG are expressed in molars (M) with fluxes being written as M/min, while all other fluxes are written in terms of the rate of change of absolute abundance (numbers/min). QR1, QR2 and QR3 are zero-order receptor synthesis fluxes (numbers/min), which are calculated so as to maintain steady-state levels of EGFR/HER1, HER2 and HER3 in the absence of ligand addition (see next section on *Initial Conditions*). Terms of type "kt<x>" and "ke<x>" are internalization rates for monomers and dimers respectively in min^{-1} with the x specifying the species that the rate applies to. Terms of type "kr<x>", "kl<x>" and "kd<x>" correspond to rate constants for recycling from the EE to the surface, sorting from the EE to the LE, and degradation from the LE, respectively. These rate constants are calculated from the species-specific kx, f and δ values. For any given species i, we write the recycling rate kri, the LE entry rate kli, and the degradation rate kdi, as $kri = kxi\, fi\,(1 + \delta i)$; $kli = kxi\,(1 - fi)(1+\delta i)$ and $kdi = kxi\,(1- fi)(1 + \delta i)/\delta i$ (see [2]). Given a single parameter $\delta 1$, the δ value for the EGFR monomer, the δ for any species i is calculated using the expression $1+ 1/\delta i = [kx1(1-f1)(1 + 1/\delta 1)]/[kxi\,(1 - fi)]$ where $kx1$ and $f1$ are the reported endosomal exit rates and recycling fractions for the EGFR monomer; kxi and fi are corresponding exit rates and recycling fractions for the species i.

kx and *f* values for various species can be found in Table S3. The single δ value $\delta 1$ is estimated as part of the model optimization, and parameter estimates are provided in Table S4. In the following equations species at the cell surface, EE and LE are denoted "<S>s", "<S>e" and "S<l>" respectively. The specific symbols "S" used for the various species are explained in the flux expression for reactions at the cell surface.

FLUXES FOR SPECIES AT THE CELL SURFACE

Flux for EGF (E) at the Cell Surface in M/min:

```
dEs_dt = (- v1s + v2s - v3s + v4s - v5s + v6s + v13s + v14s + v16s)/(Nav*Vmc)
         - kte*Es + kre*Ee*Ve/Vmc
```

Flux for HRG (H) at the Cell Surface in M/min:

```
dHs_dt = (- v7s + v8s - v9s + v10s - v11s + v12s + v15s + v17s
         + v18s)/(Nav*Vmc) - kth*Hs + krh*He*Ve/Vmc
```

Flux for unbound EGFR monomer (R1) at the Cell Surface:

```
dR1s_dt = - v1s + v2s + 2*v13s + v14s + v16s + v17s - v21s + v22s - v35s
          + v36s - kt1*R1s + kr1*R1e + QR1
```

Flux for HER2 monomer (R2) at the Cell Surface:

```
dR2s_dt =  v14s + v15s - 2*v25s + 2*v26s - v23s + v24s - v27s + v28s
          - kt2*R2s + kr2*R2e + QR2
```

Flux for HER3 monomer (R3) at the Cell Surface:

```
dR3s_dt = - v7s + v8s + v15s + v16s + v17s + 2*v18s - v31s + v32s - v33s
          + v34s - kt3*R3s + kr3*R3e + QR3
```

Flux for EGF-bound EGFR monomer (R1E) at the Cell Surface:

```
dR1Es_dt =  v1s - v2s - 2*v19s + 2*v20s - v21s + v22s - v23s + v24s - v33s
          + v34s - v37s + v38s - kt1e*R1Es + kr1e*R1Ee
```

Flux for HRG-bound HER3 monomer (R3H) at the Cell Surface:

```
dR3Hs_dt =  v7s - v8s - 2*v29s + 2*v30s - v27s + v28s - v31s + v32s - v35s
          + v36s - v37s + v38s - kt3h*R3Hs + kr3h*R3He
```

Flux for single EGF bound EGFR homodimer (R11E) at the Cell Surface:

```
dR11Es_dt = - v3s + v4s + v21s - v22s - v13s - ke11e*R11Es + kr11e*R11Ee
```

Flux for EGFR homodimer with 2 bound EGFs (R11EE) at the Cell Surface:

```
dR11EEs_dt =  v3s - v4s + v19s - v20s - ke11ee*R11EEs + kr11ee*R11EEe
```

Flux for single EGF bound EGFR-HER2 dimer (R12E) at the Cell Surface:

```
dR12Es_dt =  v23s - v24s - v14s - ke12e*R12Es + kr12e*R12Ee
```

Flux for HER2-HER2 homodimer (R22) at the Cell Surface:

```
dR22s_dt =  v25s - v26s - ke22*R22s + kr22*R22e
```

Flux for HRG bound HER2-HER3 dimer (R23H) at the Cell Surface:

```
dR23Hs_dt =  v27s - v28s - v15s - ke23h*R23Hs + kr23h*R23He
```

Flux for single HRG bound HER3-HER3 dimer (R33H) at the Cell Surface:

```
dR33Hs_dt = - v9s + v10s + v31s - v32s - v18s - ke33h*R33Hs + kr33h*R33He
```

Flux for HER3-HER3 homodimer with 2 bound HRGs (R33HH) at the Cell surface:

```
dR33HHs_dt =  v9s - v10s + v29s - v30s - ke33hh*R33HHs + kr33hh*R33HHe
```

Flux for EGF bound EGFR-HER3 dimer (R13E) at the Cell Surface:

```
dR13Es_dt =  - v11s + v12s + v33s - v34s - v16s - ke13e*R13Es + kr13e*R13Ee
```

Flux for HRG bound EGFR-HER3 dimer (R13H) at the Cell Surface:

```
dR13Hs_dt =  - v5s + v6s + v35s - v36s - v17s - ke13h*R13Hs + kr13h*R13He
```

Flux for EGF and HRG bound EGFR-HER3 dimer (R13EH) at the Cell Surface:

```
dR13EHs_dt =  v11s - v12s + v5s - v6s + v37s - v38s - ke13eh*R13EHs
             + kr13eh*R13EHe
```

FLUXES FOR SPECIES IN THE EE

```
dEe_dt = (- v1e + v2e - v3e + v4e - v5e + v6e + v13e + v14e + v16e
          )/(Nav*Ve) + kte*Es*Vmc/Ve - kre*Ee - kle*Ee

dHe_dt = (- v7e + v8e - v9e + v10e - v11e + v12e + v15e + v17e + v18e
          )/(Nav*Ve) + kth*Hs*Vmc/Ve - krh*He - klh*He

dR1e_dt = - v1e + v2e + 2*v13e + v14e + v16e + v17e - v21e + v22e - v35e
          + v36e + kt1*R1s - kr1*R1e - kl1*R1e

dR2e_dt = v14e + v15e - 2*v25e + 2*v26e - v23e + v24e - v27e + v28e
          + kt2*R2s - kr2*R2e - kl2*R2e

dR3e_dt = - v7e + v8e + v15e + v16e + v17e + 2*v18e - v31e + v32e - v33e
          + v34e + kt3*R3s - kr3*R3e - kl3*R3e

dR1Ee_dt = v1e - v2e - 2*v19e + 2*v20e - v21e + v22e - v23e + v24e - v33e
          + v34e - v37e + v38e + kt1e*R1Es - kr1e*R1Ee - kl1e*R1Ee

dR3He_dt = v7e - v8e - 2*v29e + 2*v30e - v27e + v28e - v31e + v32e - v35e
          + v36e - v37e + v38e + kt3h*R3Hs - kr3h*R3He - kl3h*R3He

dR11Ee_dt = - v3e + v4e + v21e - v22e - v13e + ke11e*R11Es - kr11e*R11Ee
          - kl11e*R11Ee

dR11EEe_dt = v3e - v4e + v19e - v20e + ke11ee*R11EEs - kr11ee*R11EEe
          - kl11ee*R11EEe

dR12Ee_dt =  v23e - v24e - v14e + ke12e*R12Es - kr12e*R12Ee - kl12e*R12Ee

dR22e_dt =  v25e - v26e + ke22*R22s - kr22*R22e - kl22*R22e

dR23He_dt =  v27e - v28e - v15e + ke23h*R23Hs - kr23h*R23He - kl23h*R23He

dR33He_dt = - v9e + v10e + v31e - v32e - v18e + ke33h*R33Hs - kr33h*R33He
            - kl33h*R33He
```

```
dR33HHe_dt = v9e - v10e + v29e - v30e + ke33hh*R33HHs - kr33hh*R33HHe
            - kl33hh*R33HHe

dR13Ee_dt = - v11e + v12e + v33e - v34e - v16e + ke13e*R13Es - kr13e*R13Ee
            - kl13e*R13Ee

dR13He_dt = - v5e + v6e + v35e - v36e - v17e + ke13h*R13Hs - kr13h*R13He
            - kl13h*R13He

dR13EHe_dt = v11e - v12e + v5e - v6e + v37e - v38e + ke13eh*R13EHs
            - kr13eh*R13EHe - kl13eh*R13EHe
```

FLUXES FOR SPECIES IN THE LE

```
dEl_dt = kle*Ee*Ve/Vl - kde*El

dHl_dt = klh*He*Ve/Vl - kdh*Hl

dR1l_dt = kl1*R1e - kd1*R1l

dR2l_dt = kl2*R2e - kd2*R2l

dR3l_dt = kl3*R3e - kd3*R3l

dR1El_dt = kl1e*R1Ee - kd1e*R1El

dR3Hl_dt = kl3h*R3He - kd3h*R3Hl

dR11El_dt = kl11e*R11Ee - kd11e*R11El

dR11EEl_dt = kl11ee*R11EEe - kd11ee*R11EEl

dR12El_dt = kl12e*R12Ee - kd12e*R12El

dR22l_dt = kl22*R22e - kd22*R22l

dR23Hl_dt = kl23h*R23He - kd23h*R23Hl

dR33Hl_dt = kl33h*R33He - kd33h*R33Hl

dR33HHl_dt = kl33hh*R33HHe - kd33hh*R33HHl

dR13El_dt = kl13e*R13Ee - kd13e*R13El

dR13Hl_dt = kl13h*R13He - kd13h*R13Hl

dR13EHl_dt = kl13eh*R13EHe - kd13eh*R13EHl
```

2. Initial conditions

The initial conditions of the system prior to ligand addition were determined by solving the rate equations for the steady-state of the system in the absence of ligand. Given the expression

levels for EGFR, HER1 and HER2, (R1t0, R2t0, R3t0, respectively; see Table S5 for HER expression levels in various cell lines), we placed all of the receptors at the cell surface to begin with. The abundances of all other species were set to 0. The receptor synthesis rates were set equal to the degradation rates of the respective receptors (i.e. QR1 = kd1*R1l, QR2= kd2*R2l + 2*kd22*R22l; QR3 = kd3*R3l). We then solved the system until steady-state levels were obtained for receptor numbers at the cell surface, EE and the LE. This results in steady-state levels of EGFR, HER2 and HER3 monomers as well as HER2 homodimers (R22) in the 3 cellular compartments. These receptor numbers were used for the ligand addition simulations where the Es0 and Hs0 were respectively set equal to the added EGF and HRG doses. The QR1, QR2 and QR3 receptor synthesis values obtained for the no ligand steady state were then used as constant synthesis rates during the ligand addition simulations.

3. Calculating HER receptor mass and phosphorylation levels from the model variables

This section describes how the model variables were used to compute receptor mass and phosphorylation levels in a form that could be compared directly with the experimental data. We also describe calculation of the fractional contributions of various dimers to the phosphorylation levels of the HER1-3 receptors.

For receptor mass, we first calculated the absolute numbers of EGFR (R1c), HER2 (R2c) and HER3 (R3c) in the cell by combining appropriate model variables as follows:

```
Total EGFR abundance (numbers/cell)
R1c =
    R1s + R1Es + 2*R11Es + 2*R11EEs + R12Es + R13Es + R13Hs + R13EHs +
    R1e + R1Ee + 2*R11Ee + 2*R11EEe + R12Ee + R13Ee + R13He + R13EHe +
    R1l + R1El + 2*R11El + 2*R11EEl + R12El + R13El + R13Hl + R13EHl

Total EGFR abundance (numbers/cell)
R2c =
    R2s + 2*R22s + R12Es + R23Hs +
    R2e + 2*R22e + R12Ee + R23He +
    R2l + 2*R22l + R12El + R23Hl

Total EGFR abundance (numbers/cell)
R3c =
    R3s + R3Hs + 2*R33Hs + 2*R33HHs + R23Hs + R13Es + R13Hs + R13EHs +
    R3e + R3He + 2*R33He + 2*R33HHe + R23He + R13Ee + R13He + R13EHe +
    R3l + R3Hl + 2*R33Hl + 2*R33HHl + R23Hl + R13El + R13Hl + R13EHl
```

These absolute receptor numbers were then scaled to convert them into the units for the ELISA receptor abundance measurements as follows:

Total EGFR abundance (ELISA units)
```
mR1t = R1c*mf1,  with mf1 = mR10(Parental)/2e5
```

Total HER2 abundance (ELISA units)
```
mR2t = R2c*mf2,  with mf2 = mR20(24H)/6e5
```

Total HER3 abundance (ELISA units)
```
mR3t = R3c*mf3,  with mf3 = mR30(B5)/2.8e4
```

In the above expressions mf1, mf2 and mf3 are mass scaling factors for EGFR, HER2 and HER3. mR10(Parental) is the experimentally determined value for the EGFR receptor mass (in ELISA units) in the Parental cell in the absence of ligand addition. Similarly, mR20(24H) is the ELISA measurement for the HER2 receptor mass in the 24H (HER2+3−) cell in the absence of ligand addition, and mR30(B5) is the ELISA measurement for the HER3 receptor mass in the B5 (HER2−3+) cell in the absence of ligand addition. Here, we take advantage of the previously estimated values for the absolute EGFR number in the Parental cell [3]; the HER2 number in the 24H cell [3], and the HER3 number in the B5 cell [4]. The time dependent predictions for mR1t, mR2t and mR3t calculated above were compared with the corresponding experimental data for the HME cells to construct residual vectors to be minimized during parameter estimation.

The dimer abundances predicted by the model, and the 26 distinct phosphorylation efficiency factors (pf) values (see Table S4) were used to determine the HER1-3 phosphorylation signals from the various dimers (product of dimer abundance and appropriate pf value), and the total HER1-3 phosphorylation levels (sum of the relevant dimer phosphorylation signals) as described below.

Specifically, model predictions for the EGFR phosphorylation levels in the various cellular compartments ("s", surface; "i", internal; and "t", total) from the different EGFR containing dimer species were calculated as follows:

EGFR phosphorylation signal from R11E (ELISA units)
```
pR1i_11e = 2*pf11ei*R11Ee          (internal signal)
pR1s_11e = 2*pf11es*R11Es          (surface signal)
pR1t_11e = pR1i_11e + pR1s_11e     (total signal)
```

EGFR phosphorylation signal from R11EE (ELISA units)
```
pR1i_11ee = 2*pf11eei*R11EEe
pR1s_11ee = 2*pf11ees*R11EEs
pR1t_11ee = pR1i_11ee + pR1s_11ee
```

EGFR phosphorylation signal from R12E (ELISA units)
```
pR1i_12e = pf12ei*R12Ee
pR1s_12e = pf12es*R12Es
pR1t_12e = pR1i_12e + pR1s_12e
```

EGFR phosphorylation signal from R13E (ELISA units)
```
pR1i_13e = pf13ei*R13Ee
pR1s_13e = pf13es*R13Es
pR1t_13e = pR1i_13e + pR1s_13e
```

EGFR phosphorylation signal from R13H (ELISA units)
```
pR1i_13h = pf13hi*R13He
pR1s_13h = pf13hs*R13Hs
pR1t_13h = pR1i_13h + pR1s_13h
```

EGFR phosphorylation signal from R13EH (ELISA units)
```
pR1i_13eh = pf13ehi*R13EHe
pR1s_13eh = pf13ehs*R13EHs
pR1t_13eh = pR1i_13eh + pR1s_13eh
```

In the notation used above specific pf values are written as pf<*ijc*> where *i* and *j* specify the HER types in the dimer; *i* is the receptor whose phosphorylation level is being computed; *c* is the cell compartment. The total EGFR phosphorylation levels were computed from the above dimer contributions as follows:

Total EGFR phosphorylation signal (ELISA units):
```
pR1i = pR1i_11e + pR1i_11ee + pR1i_12e
     + pR1i_13e + pR1i_13h + pR1i_13eh                      (internal)

pR1s = pR1s_11e + pR1s_11ee + pR1s_12e
     + pR1s_13e + pR1s_13h + pR1s_13eh                      (surface)

pR1t = pR1t_11e + pR1t_11ee + pR1t_12e
     + pR1t_13e + pR1t_13h + pR1t_13eh                      (total)
```

Expressions for the HER2 phosphorylation signal from the various HER2 dimers, and the total HER2 phosphorylation level are as follows:

HER2 phosphorylation signal from R22 (ELISA units)
```
pR2i_22 = 2*pf22i*R22e        (internal signal)
pR2s_22 = 2*pf22s*R22s        (surface signal)
pR2t_22 = pR2i_22 + pR2s_22   (total signal)
```

HER2 phosphorylation signal from R12E (ELISA units)
```
pR2i_12e = pf21ei*R12Ee
pR2s_12e = pf21es*R12Es
pR2t_12e = pR2i_12e + pR2s_12s
```

HER2 phosphorylation signal from R23H (ELISA units)
```
pR2i_23h = pf23hi*R23He
```

```
pR2s_23h = pf23hs*R23Hs
pR2t_23h = pR2i_23h + pR2s_23h
```

Total HER2 phosphorylation signal (ELISA units)

```
pR2i = pR2i_22 + pR2i_12e + pR2i_23h                  (internal)
pR2s = pR2s_22 + pR2s_12e + pR2s_23h                  (surface)
pR2t = pR2t_22 + pR2t_12e + pR2t_23h                  (total)
```

Expressions for the HER3 phosphorylation signal from the various HER3 dimers, and the total HER3 phosphorylation level are as follows:

HER3 phosphorylation signal from R13E (ELISA units)

```
pR3i_13e = pf31ei*R13Ee            (internal signal)
pR3s_13e = pf31es*R13Es            (surface signal)
pR3t_13e = pR3i_13e + pR3s_13e     (total signal)
```

HER3 phosphorylation signal from R13H (ELISA units)

```
pR3i_13h = pf31hi*R13He
pR3s_13h = pf31hs*R13Hs
pR3t_13e = pR3i_13h + pR3s_13h
```

HER3 phosphorylation signal from R13EH (ELISA units)

```
pR3i_13eh = pf31ehi*R13EHe
pR3s_13eh = pf31ehs*R13EHs
pR3t_13eh = pR3i_13eh + pR3s_13eh
```

HER3 phosphorylation signal from R23H (ELISA units)

```
pR3i_23h = pf32hi*R23He
pR3s_23h = pf32hs*R23Hs
pR3t_23h = pR3i_23h + pR3s_23h
```

Total HER3 phosphorylation signal (ELISA units)

```
pR3i = pR3i_13e + pR3i_13h + pR3i_13eh + pR3i_23h     (internal)
pR3s = pR3s_13e + pR3s_13h + pR3s_13eh + pR3s_23h     (surface)
pR3t = pR3t_13e + pR3t_13h + pR3t_13eh + pR3t_23h     (total)
```

The time dependent predictions for pR1i, pR2i, pR3i, pR1t, pR2t and pR3t calculated above were compared with the corresponding experimental data for the HME cells to construct residual vectors to be minimized during parameter estimation. Further, the above results were used to calculate the % contributions of the various dimer types to EGFR phosphorylation. For instance the % contribution of EGFR homodimers to EGFR phosphorylation was calculated as 100* (pR1t_11e + pR1t_11ee)/pR1t.

4. Estimation of HER receptor expression levels for various cell lines

We used absolute expression levels (in molecules/cell) of EGFR, HER2 and HER3 from the literature, where available, for various cell lines [5,6,7]. All such numbers used here were determined in previous studies using quantitative flow cytometry. Neve et al. [8] have published

a Western blot-based relative quantification of EGFR, HER2 and HER3 protein levels in a large panel of cell lines. In order to convert these numbers to absolute receptor expression levels, we considered cell lines for which both types of information (Western blot measurements of protein levels, HER expression levels in numbers/cell) were available, and plotted the absolute HER expression levels against the corresponding Western blot values (Fig. S17). As seen, a linear relationship was observed for EGFR (Fig. S17A) and HER2 (Fig. S17B). Hence, we used a fitted linear regression line (black dotted lines in panels Figs. S17A and S17B) to derive absolute receptor numbers from Western blot data for these receptors. For HER3 (Fig. S17C), cells that expressed the receptor appeared to all have roughly the same absolute expression level. Hence, we assumed that all Western blot measurements below a certain threshold (see red dotted line) corresponded to the absence of HER3; with measurements above this threshold indicating an HER3 level of 38,000 receptors/cell (mean expression level in HER3+ cells; see black dotted line in Fig. S17C). These relationships were then used to convert the Western blot measurements of EGFR, HER2 and HER3 from Neve et al. [8] to absolute receptor expression levels. The expression levels for various cell lines used in our analysis are presented in Table S5. These numbers are also plotted in Fig. S18 to illustrate the range of variation for EGFR, HER2 and HER3 expression levels across the different cell lines.

5. Regression analysis of Erk and Akt phosphorylation

Multilinear regression models were fit to Erk and Akt activation data collected in the panel of HME cell lines to determine the relationship between receptor activation and the activation of these downstream molecules. pERK and pAKT levels were measured as a function of time following stimulation of Parental and 24H cells with 12 ng/ml EGF. B5 and D20 cells were treated with 12ng/ml EGF and 40 ng/ml HRG alone or in combination. pERK and pAKT levels were measured using ELISA experiments and were normalized using total protein measurements as described in [4], and are expressed in arbitrary scaled units.

Regression analysis used the receptor activation or the activation of the receptor dimers as predictors of pERK and pAKT. Specifically, we used the time dependent phosphorylation levels from various receptor dimers (obtained from our mathematical model for HER activation) as the predictors for Erk and Akt levels. Predictions of the regression models (Table S6) were compared to the experimental data for phospho-ERK (pERK) and phospho-AKT (pAKT)

collected in our HME cell lines (Figures S24 and S25). Because the phospho-receptor levels are measured and normalized differently, and since each is in arbitrary units, the coefficients of the utilized regression expressions $pT(t) = b0 + \sum b_i * pR_i(t)$ and $pT(t) = b0 + \sum b_{i\_ji} * pR_{i\_ji}(t)$ cannot be compared to each other. I.e., the magnitudes of the regression coefficients b (Table S6) are not meaningful and the criteria for evaluating the importance of a receptor contribution are the p-values.

Alternative regression models where the predictor terms with negligible importance were left out of the analysis was also pursued. Validity of the considered models were compared using the Akaike Information Criterion (AIC) [2]. Table S6 and Figures S24 and S25 also report the results of these alternative models with the lowest, i.e., the best AIC score. For example, analysis of pAKT (Table S6) indicated that a model with only two distinct dimer signals as the predictors

$pAKT(t) = b_0 + b_{1\_13}*pR_{1\_13}(t) + b_{3\_13}*pR_{3\_13}(t)$ (with AIC = 223; Model 6)

was a more powerful predictor than a model that included all the receptor types but omitted the dimers

$pAKT(t) = b_0 + b_1*pR_1 (t) + b_2*pR_2(t) + b_3*pR_3(t)$ (with AIC = 242; Model 1).

This analysis clearly illustrated that activation patterns of the HER signaling pathways require the ability to distinguish the stimulation through different dimer complexes. Obtaining this information directly from experiments is nearly infeasible. In this respect, integrated studies based on model-based analysis can fulfill a critical role.

6. References

1. Hendriks BS, Wiley HS, Lauffenburger D (2003) HER2-mediated effects on EGFR endosomal sorting: Analysis of biophysical mechanisms. Biophys J 85: 2732-2745.
2. Shankaran H, Zhang Y, Chrisler WB, Ewald JA, Wiley HS, et al. (2012) Integrated experimental and model-based analysis reveals the spatial aspects of EGFR activation dynamics. Mol Biosyst 8: 2868-2882.
3. Hendriks BS, Opresko LK, Wiley HS, Lauffenburger D (2003) Coregulation of epidermal growth factor receptor/human epidermal growth factor receptor 2 (HER2) levels and locations: Quantitative analysis of HER2 overexpression effects. Cancer Research 63: 1130-1137.
4. Zhang Y, Opresko L, Shankaran H, Chrisler WB, Wiley HS, et al. (2009) HER/ErbB receptor interactions and signaling patterns in human mammary epithelial cells. BMC Cell Biol 10: 78.
5. DeFazio-Eli L, Strommen K, Dao-Pick T, Parry G, Goodman L, et al. (2011) Quantitative assays for the measurement of HER1-HER2 heterodimerization and phosphorylation in cell lines and breast tumors: applications for diagnostics and targeted drug mechanism of action. Breast Cancer Res 13: R44.
6. McDonagh CF, Huhalov A, Harms BD, Adams S, Paragas V, et al. (2012) Antitumor activity of a novel bispecific antibody that targets the ErbB2/ErbB3 oncogenic unit and inhibits heregulin-induced activation of ErbB3. Mol Cancer Ther 11: 582-593.
7. Mukherjee A, Badal Y, Nguyen XT, Miller J, Chenna A, et al. (2011) Profiling the HER3/PI3K pathway in breast tumors using proximity-directed assays identifies correlations between protein complexes and phosphoproteins. PLoS One 6: e16443.
8. Neve RM, Chin K, Fridlyand J, Yeh J, Baehner FL, et al. (2006) A collection of breast cancer cell lines for the study of functionally distinct cancer subtypes. Cancer Cell 10: 515-527.
9. French AR, Tadaki DK, Niyogi SK, Lauffenburger DA (1995) Intracellular trafficking of epidermal growth factor family ligands is directly influenced by the pH sensitivity of the receptor/ligand interaction. J Biol Chem 270: 4334-4340.
10. Jones JT, Akita RW, Sliwkowski MX (1999) Binding specificities and affinities of egf domains for ErbB receptors. FEBS Lett 447: 227-231.

11. Karunagaran D, Tzahar E, Liu N, Wen D, Yarden Y (1995) Neu differentiation factor inhibits EGF binding. A model for trans-regulation within the ErbB family of receptor tyrosine kinases. J Biol Chem 270: 9982-9990.
12. Kholodenko BN, Demin OV, Moehren G, Hoek JB (1999) Quantification of short term signaling by the epidermal growth factor receptor. J Biol Chem 274: 30169-30181.
13. Hendriks BS, Opresko LK, Wiley HS, Lauffenburger D (2003) Quantitative analysis of HER2-mediated effects on HER2 and epidermal growth factor receptor endocytosis: distribution of homo- and heterodimers depends on relative HER2 levels. J Biol Chem 278: 23343-23351.
14. Hendriks BS, Cook J, Burke JM, Beusmans JM, Lauffenburger DA, et al. (2006) Computational modelling of ErbB family phosphorylation dynamics in response to transforming growth factor alpha and heregulin indicates spatial compartmentation of phosphatase activity. Syst Biol (Stevenage) 153: 22-33.

SUPPLEMENTAL TABLES

Table S1. Biochemical reactions at the cell surface: Rate expressions and parameter values

REACTION #	REACTION	FLUX EXPRESSION	PARAMETER	VALUE
Reversible binding of EGF to EGFR in monomers and dimers				
1s [1]	R1s + Es → R1Es	v1s = kon1s [R1s] [Es]	kon1s (/M/min)	6.3×10^{7}
2s [2]	R1Es → R1s + Es	v2s = koff1s [R1Es]	koff1s (/min)	0.12
3s [3]	R11Es + Es → R11EEs	v3s = kon11es [R11Es] [Es]	kon11es (/M/min)	6.3×10^{7}
4s [3]	R11EEs → R11Es + Es	v4s = koff11es [R1EEs]	koff11es (/M/min)	0.12
5s [3]	R13Hs + Es → R13EHs	v5s = kon13hs [R11Es] [Es]	kon13hs (/M/min)	6.3×10^{7}
6s [4]	R13EHs → R13Hs + Es	v6s = koff13hs [R1EEs]	koff13hs (/M/min)	0.36
Reversible binding of HRG to HER3 in monomers and dimers				
7s [5]	R3s + Hs → R3Hs	v7s = kon3s [R3s] [Hs]	kon3s (/M/min)	6.3×10^{7}
8s [2]	R3Hs → R3s + Hs	v8s = koff3s [R3Hs]	koff3s (/min)	0.34
9s [3]	R33Hs + Hs → R33HHs	v9s = kon33hs [R33Hs] [Hs]	kon33hs (/M/min)	6.3×10^{7}
10s [3]	R33HHs → R33Hs + Hs	v10s = koff33hs [R33HHs]	koff33hs (/min)	0.34
11s [3]	R13Es + Hs → R13EHs	v11s = kon13es [R13Es] [Hs]	kon13es (/M/min)	6.3×10^{7}
12s [3]	R13EHs → R13Es + Hs	v12s = koff13es [R13EHs]	koff13es (/min)	0.34
Irreversible ligand dissociation from single ligand bound dimers				
13s [3]	R11Es → 2R1s + Es	v13s = koffi11es [R11Es]	koffi11es (/min)	0.12
14s [6]	R12Es → R1s + R2s + Es	v14s = koffi12es [R12Es]	koffi12es (/min)	0.072
15s [7]	R23Hs → R2s + R3s + Hs	v15s = koffi23hs [R23Hs]	koffi23hs (/min)	0.014
16s [3]	R13Es → R1s + R3s + Es	v16s = koffi13es [R13Es]	koffi13es (/min)	0.12
17s [3]	R13Hs → R1s + R3s + Hs	v17s = koffi13hs [R13Hs]	koffi13hs (/min)	0.34
18s [3]	R33Hs → 2R3s + Hs	v18s = koffi33hs [R33Hs]	koffi33hs (/min)	0.34
Reversible dimerization				
19s [8]	R1Es + R1Es → R11EEs	v19s = kc [R1Es][R1Es]	kc [$(\#/cell)^{-1}$ min^{-1}]	2×10^{-4}
20s [9]	R11EEs → R1Es + R1Es	v20s = ku11ees [R11EEs]	ku11ees (/min)	Unknown
21s	R1Es + R1s → R11Es	v21s = kc [R1Es][R1s]	kc [$(\#/cell)^{-1}$ min^{-1}]	2×10^{-4}
22s [9]	R11Es → R1Es + R1s	v22s = ku11es [R11Es]	ku11es (/min)	Unknown
23s	R1Es + R2s → R12Es	v23s = kc [R1Es][R2s]	kc [$(\#/cell)^{-1}$ min^{-1}]	2×10^{-4}
24s [9]	R13Es → R1Es + R2s	v24s = ku12es [R12Es]	ku12es (/min)	Unknown
25s	R2s + R2s → R22s	v25s = kc [R2s][R2s]	kc [$(\#/cell)^{-1}$ min^{-1}]	2×10^{-4}
26s [9]	R22s → R2s + R2s	v26s = ku22s [R22s]	ku22s (/min)	Unknown
27s	R2s + R3Hs → R23Hs	v27s = kc [R2s][R3Hs]	kc [$(\#/cell)^{-1}$ min^{-1}]	2×10^{-4}
28s [9]	R23Hs → R2s + R3Hs	v28s = ku23hs [R23Hs]	ku23hs (/min)	Unknown
29s	R3Hs + R3Hs → R33HHs	v29s = kc [R3Hs][R3Hs]	kc [$(\#/cell)^{-1}$ min^{-1}]	2×10^{-4}
30s [9]	R33HHs → R3Hs + R3Hs	v30s = ku33hhs [R33HHs]	ku33hhs (/min)	Unknown

31s	R3Hs + R3s → R33Hs	v31s = kc [R3Hs][R3s]	kc [(#/cell)$^{-1}$ min^{-1}]	2×10^{-4}
32s [9]	R33Hs → R3Hs + R3s	v32s = ku33hs [R33Hs]	ku33hs (/min)	Unknown
33s	R1Es + R3s → R13Es	v33s = kc [R1Es][R3s]	kc [(#/cell)$^{-1}$ min^{-1}]	2×10^{-4}
34s [9]	R13Es → R1Es + R3s	v34s = ku13es [R13Es]	ku13es (/min)	Unknown
35s	R1s + R3Hs → R13Hs	v35s = kc [R1s][R3Hs]	kc [(#/cell)$^{-1}$ min^{-1}]	2×10^{-4}
36s [9]	R13Hs → R1s + R3Hs	v36s = ku13hs [R13Hs]	ku13hs (/min)	Unknown
37s	R1Es + R3Hs → R13EHs	v37s = kc [R1s][R3Hs]	kc [(#/cell)$^{-1}$ min^{-1}]	2×10^{-4}
38s [9]	R13EHs → R1Es + R3Hs	v38s = ku13ehs [R13EHs]	ku13ehs (/min)	Unknown

[1] From [9]

[2] Set to match the Kd value reported in [10]

[3] Rate constants for EGF and HRG binding and dissociation assumed to be same for receptor monomers and dimers. Hence these rates are set equal to kon1s or koff1s for EGF binding/dissociation, and kon3s, koff3s for HRG binding/dissociation as appropriate.

[4] Based on Karunagaran et al. [11]: Presence of HRG is assumed to decrease the EGF binding affinity of the R13 dimer by a factor of 3 (koff is set to 3*koff1s)

[5] Assumed that kon is the same for the HRG-HER3 and EGF-EGFR interactions. koff is set to match Kd reported in the literature [10]

[6] Assumed equal to 0.6*koff1s; Based on Jones et al [10] the R12 dimer is assumed to have a stronger affinity for EGF compared to the R11 dimer

[7] Assumed equal to 0.04*koff3s; Based on Jones et al [10], the R23 dimer is assumed to have a 25-fold stronger affinity for HRG compared to HER3 alone

[8] kc was assumed to be 1/5th of the diffusion-limited encounter rate reported in Kholodenko et al [12]

[9] These dimer dissociation parameters were treated as unknowns and estimated by fitting the model to experimental data in the current study

Table S2. Biochemical reactions in the early endosomes: Rate expressions and parameter values

REACTION #	REACTION	FLUX EXPRESSION	PARAMETER	VALUE
Reversible binding of EGF to EGFR in monomers and dimers				
1e [1]	R1e + Ee → R1Ee	v1e = kon1i [R1e] [Ee]	kon1i (/M/min)	8.5×10^{6}
2e [1]	R1Ee → R1e + Ee	v2e = koff1i [R1Ee]	koff1i (/min)	0.66
3e [2]	R11Ee + Ee → R11EEe	v3e = kon11ei [R11Ee] [Ee]	kon11ei (/M/min)	8.5×10^{6}
4e [2]	R11EEe → R11Ee + Ee	v4e = koff11ei [R1EEe]	koff11ei (/M/min)	0.66
5e [2]	R13He + Ee → R13EHe	v5e = kon13hi [R11Ee] [Ee]	kon13hi (/M/min)	8.5×10^{6}
6e [3]	R13EHe → R13He + Ee	v6e = koff13hi [R1EEe]	koff13hi (/M/min)	1.98
Reversible binding of HRG to HER3 in monomers and dimers				
7e [4]	R3e + He → R3He	v7e = kon3i [R3e] [He]	kon3i (/M/min)	8.5×10^{6}
8e [4]	R3He → R3e + He	v8e = koff3i [R3He]	koff3i (/min)	0.66
9e [2]	R33He + He → R33HHe	v9e = kon33hi [R33He] [He]	kon33hi (/M/min)	8.5×10^{6}
10e [2]	R33HHe → R33He + He	v10e = koff33hi [R33HHe]	koff33hi (/min)	0.66
11e [2]	R13Ee + He → R13EHe	v11e = kon13ei [R13Ee] [He]	kon13ei (/M/min)	8.5×10^{6}

12e [2]	R13EHe → R13Ee + He	v12e = koff13ei [R13EHe]	koff13ei (/min)	0.66
Irreversible ligand dissociation from single ligand bound dimers				
13e [2]	R11Ee → 2R1e + Ee	v13e = koffi11ei [R11Ee]	koffi11ei (/min)	0.66
14e [5]	R12Ee → R1e + R2e + Ee	v14e = koffi12ei [R12Ee]	koffi12ei (/min)	0.396
15e [6]	R23He → R2e + R3e + He	v15e = koffi23hi [R23He]	koffi23hi (/min)	0.026
16e [2]	R13Ee → R1e + R3e + Ee	v16e = koffi13ei [R13Ee]	koffi13ei (/min)	0.66
17e [2]	R13He → R1e + R3e + He	v17e = koffi13hi [R13He]	koffi13hi (/min)	0.66
18e [2]	R33He → 2R3e + He	v18e = koffi33hi [R33He]	koffi33hi (/min)	0.66
Reversible dimerization				
19e [7]	R1Ee + R1Ee → R11EEe	v19e = kc [R1Ee][R1Ee]	kc [$(\#/\text{cell})^{-1}$ min^{-1}]	2×10^{-4}
20e [8]	R11EEe → R1Ee + R1Ee	v20e = ku11eei [R11EEe]	ku11eei (/min)	Unknown
21e	R1Ee + R1e → R11Ee	v21s = kc [R1Ee][R1e]	kc [$(\#/\text{cell})^{-1}$ min^{-1}]	2×10^{-4}
22e [8]	R11Ee → R1Ee + R1e	v22s = ku11ei [R11Ee]	ku11ei (/min)	Unknown
23e	R1Ee + R2e → R12Ee	v23s = kc [R1Ee][R2e]	kc [$(\#/\text{cell})^{-1}$ min^{-1}]	2×10^{-4}
24e [8]	R13Ee → R1Ee + R2e	v24s = ku12ei [R12Ee]	ku12ei (/min)	Unknown
25e	R2e + R2e → R22e	v25s = kc [R2e][R2e]	kc [$(\#/\text{cell})^{-1}$ min^{-1}]	2×10^{-4}
26e [8]	R22e → R2e + R2e	v26s = ku22i [R22e]	ku22i (/min)	Unknown
27e	R2e + R3He → R23He	v27s = kc [R2e][R3He]	kc [$(\#/\text{cell})^{-1}$ min^{-1}]	2×10^{-4}
28e [8]	R23He → R2e + R3He	v28s = ku23hi [R23He]	ku23hi (/min)	Unknown
29e	R3He + R3He → R33HHe	v29s = kc [R3He][R3He]	kc [$(\#/\text{cell})^{-1}$ min^{-1}]	2×10^{-4}
30e [8]	R33HHe → R3He + R3He	v30s = ku33hhi [R33HHe]	ku33hhi (/min)	Unknown
31e	R3He + R3e → R33He	v31s = kc [R3He][R3e]	kc [$(\#/\text{cell})^{-1}$ min^{-1}]	2×10^{-4}
32e [8]	R33He → R3He + R3e	v32s = ku33hi [R33He]	ku33hi (/min)	Unknown
33e	R1Ee + R3e → R13Ee	v33s = kc [R1Ee][R3e]	kc [$(\#/\text{cell})^{-1}$ min^{-1}]	2×10^{-4}
34e [8]	R13Ee → R1Ee + R3e	v34s = ku13ei [R13Ee]	ku13ei (/min)	Unknown
35e	R1e + R3He → R13He	v35s = kc [R1e][R3He]	kc [$(\#/\text{cell})^{-1}$ min^{-1}]	2×10^{-4}
36e [8]	R13He → R1e + R3He	v36s = ku13hi [R13He]	ku13hi (/min)	Unknown
37e	R1Ee + R3He → R13EHe	v37s = kc [R1e][R3He]	kc [$(\#/\text{cell})^{-1}$ min^{-1}]	2×10^{-4}
38e [8]	R13EHe → R1Ee + R3He	v38s = ku13ehi [R13EHe]	ku13ehi (/min)	Unknown

[1] From French et al. [9]

[2] Rate constants for EGF and HRG binding and dissociation were assumed to be same for receptor monomers and dimers. Hence these rates were set equal to kon1i or koff1i for EGF binding/dissociation, and kon3i, koff3i for HRG binding/dissociation as appropriate

[3] Based on Karunagaran et al. [11]: Presence of HRG was assumed to decrease the EGF binding affinity of the R13 dimer by a factor of 3 (koff is set to 3*koff1i)

[4] The binding and dissociation rates for the HRG-HER3 interaction in the cell interior were set equal to the values for the EGF-EGFR interaction due to lack of information

[5] Assumed equal to 0.6*koff1i; Based on Jones et al. [10], the R12 dimer was assumed to have a stronger affinity for EGF compared to the R11 dimer

[6] Assumed equal to 0.04*koff3i; Based on Jones et al. [10], the R23 dimer was assumed to have a 25-fold stronger affinity for HRG compared to HER3 alone

[7] kc was assumed to be 1/5th of the diffusion-limited encounter rate reported in Kholodenko et al. [12]

[8] These dimer dissociation parameters were treated as unknowns and were estimated by fitting the model to experimental data in the current study

Table S3. Trafficking Parameters

	Internalization rates (/min)		Endosomal exit rates (/min)		Recycling Fractions	
**SPECIES**	**Parameter**	**Value**	**Parameter**	**Value**	**Parameter**	**Value**
**EGF** [2]	kte	0	kxe	0.08	fe	0.8
**HRG** [2]	kth	0	kxh	0.07	fh	0.94
**R1** [3]	kt1	0.07	kx1	0.08	f1	0.8
**R2** [3]	kt2	0.01	kx2	0.07	f2	0.94
**R3** [4]	kt3	0.01	kx3	0.07	f3	0.94
**R1E** [5]	kt1e	0.07	kx1e	0.08	f1e	0.8
**R3H** [5]	kt3h	0.01	kx3h	0.07	f3h	0.94
**R11E** [3]	ke11e	0.28	kx11e	0.03	f11e	0.5
**R11EE** [3]	ke11ee	0.28	kx11ee	0.03	f11ee	0.5
**R12E** [6]	ke12e	0.1	kx12e	0.04	f12e	0.8
**R22** [3]	ke22	0.01	kx22	0.07	f22	0.94
**R23H** [4]	ke23h	0.01	kx23h	0.07	f23h	0.94
**R33H** [4]	ke33h	0.01	kx33h	0.07	f33h	0.94
**R33HH** [4]	ke33hh	0.01	kx33hh	0.07	f33hh	0.94
**R13E** [4]	ke13e	0.1	kx13e	0.04	f13e	0.8
**R13H** [4]	ke13h	0.1	kx13h	0.04	f13h	0.8
**R13EH** [4]	ke13eh	0.1	kx13eh	0.04	f13eh	0.8

[1] Species-specific values for internalization, endosomal exit and recycling fraction are shown. In addition to these, we require a δ value for each species that specifies how it partitions between the early and late endosomes. With the assumption that once in the LE all molecules are degraded at the same rate, the δ values for all species can be calculated given the δ value for any one species. Here we determine δ_1 for the EGFR monomer as part of the parameter estimation, and calculate the values for all other species from this value as described in the *Governing Equations* section of the Supplemental Text.

[2] Pinocytosis of free ligand molecules is neglected since it is expected to be much slower compared to receptor-mediated endocytosis. Endosomal exit rates and recycling fractions for free ligands EGF and HRG are set to the values for the EGFR monomer and the HER3 monomer, respectively

[3] From [3,13]

[4] HER3 is assumed to traffic like HER2 in all respects: HER3 monomers and homodimers are assumed to traffic like the HER2 monomers and homodimers; The R13 receptor is assumed to traffic like the R12 receptor; The R23 dimer is assumed to traffic like the R22 dimer

[5] Free and ligand-bound monomers are treated as unphosphorylated species with similar trafficking properties

[6] Based on [3,13,14]

Table S4. Parameter estimates obtained by fitting the model to the experimental datasets[1]

Parameter	Par Set 1	Par Set 2	Par Set 3	Par Set 4	Par Set 5	Par Set 6	Par Set 7 [2]	Range[3]
**ku11es**	2.76E+00	6.58E+00	1.41E+00	1.49E+00	2.30E+00	9.51E-01	3.46E+00	0.84
**ku11ees**	7.43E-02	4.26E-01	1.48E-05	5.53E-04	7.42E-04	1.80E-04	3.39E-01	4.46
**ku11ei**	3.24E+01	8.69E+01	6.33E+00	2.28E+01	7.90E+01	1.05E+02	9.02E+01	1.22
**ku11eei**	2.13E-01	5.05E-03	4.48E-02	1.03E-01	2.41E-01	3.04E-01	7.02E-02	1.78
**delta1**	1.95E+00	3.57E+00	5.38E+00	1.95E+00	1.88E+00	2.54E+00	3.56E+00	0.46
**pf11es**	7.04E-03	6.19E-03	8.45E-03	6.77E-03	6.57E-03	1.05E-02	9.53E-03	0.23
**pf11ees**	3.41E-02	2.82E-02	2.24E-02	3.43E-02	2.91E-02	2.74E-02	2.84E-02	0.19
**pf11ei**	1.05E-01	3.60E-01	3.04E-02	6.79E-02	2.06E-01	2.67E-01	3.11E-01	1.07
**pf11eei**	2.61E-03	6.04E-03	1.36E-02	2.99E-03	2.77E-03	5.12E-03	7.01E-03	0.72
**ku12es**	8.60E+01	3.78E+02	2.75E+01	1.23E+02	3.51E+01	1.41E+02	1.36E+02	1.14
**ku22s**	7.40E+02	2.20E+02	1.69E+00	9.23E+02	9.78E-01	8.21E+02	9.90E+02	3.01
**ku12ei**	8.24E-01	8.50E-01	3.83E-01	3.58E-01	4.38E+00	9.89E-01	4.48E-01	1.09
**ku22i**	1.81E+00	9.54E+00	2.87E+01	9.09E-01	7.12E+02	3.15E-01	2.12E+00	3.35
**pf12es**	2.06E-01	7.36E-01	8.02E-01	3.68E-01	1.00E+00	4.32E-01	2.52E-01	0.69
**pf21es**	1.10E-01	3.90E-01	4.06E-01	2.09E-01	5.68E-01	2.52E-01	1.30E-01	0.71
**pf22s**	4.44E-05	1.18E-06	1.61E-06	1.71E-06	2.88E-06	3.30E-05	2.59E-05	1.58
**pf12ei**	9.36E-03	9.73E-03	9.23E-03	8.31E-03	9.41E-03	1.50E-02	1.04E-02	0.26
**pf21ei**	3.76E-03	3.31E-03	3.01E-03	3.24E-03	3.75E-03	6.76E-03	3.50E-03	0.35
**pf22i**	4.54E-04	6.93E-04	1.09E-03	3.83E-04	9.21E-03	3.77E-04	4.63E-04	1.39
**ku33hs**	1.87E-02	2.26E+00	9.76E+01	1.01E+02	4.81E+01	5.03E-03	5.24E+00	4.30
**ku33hhs**	9.32E-03	2.06E+02	3.29E-02	1.03E-03	4.98E+00	3.99E-01	1.49E-02	5.30
**ku13es**	6.37E+02	1.65E+02	2.48E+01	2.61E+01	6.43E+02	6.29E+01	3.75E+02	1.41
**ku13hs**	3.44E+00	7.89E-01	8.90E+01	5.42E+02	4.97E+00	1.15E+00	1.90E+00	2.84
**ku13ehs**	5.97E+01	6.92E-01	6.24E+02	2.02E+02	1.04E+00	3.17E-01	6.22E-01	3.29
**ku33hi**	7.53E+02	1.28E+02	2.91E+00	1.97E-01	2.95E-01	7.51E-03	1.11E-03	5.83
**ku33hhi**	1.65E-01	6.97E+02	3.65E+00	2.82E-01	3.88E-02	2.44E-02	2.10E-02	4.52
**ku13ei**	4.19E+02	4.11E+00	1.24E+00	1.22E+00	5.12E+02	1.35E-01	9.99E+02	3.87
**ku13hi**	5.24E+02	3.62E+01	6.69E+00	1.38E+02	5.50E+00	5.29E+01	4.67E+00	2.05
**ku13ehi**	6.29E+00	4.65E+01	4.36E+00	3.07E+00	3.34E+02	8.64E-01	1.05E+02	2.59
**pf13es**	9.86E-01	4.56E-01	1.20E-01	1.08E-01	7.90E-01	4.13E-01	8.30E-01	0.96
**pf13hs**	9.89E-04	8.94E-06	7.26E-03	6.82E-02	1.54E-04	6.99E-04	2.36E-04	3.88
**pf13ehs**	1.00E-06	1.00E-06	1.02E-06	5.81E-06	1.00E-06	1.00E-06	1.00E-06	0.76
**pf31es**	1.50E-01	1.90E-02	8.81E-03	1.04E-02	1.34E-01	2.68E-02	6.45E-02	1.23
**pf31hs**	2.28E-03	1.53E-03	2.06E-06	2.11E-02	7.55E-04	6.37E-04	1.52E-03	4.01
**pf31ehs**	1.43E-01	2.98E-06	4.47E-01	5.77E-01	1.09E-06	5.45E-05	1.85E-06	5.72
**pf13ei**	1.00E-06	1.00E-06	1.00E-06	2.00E-04	1.08E-06	1.09E-06	1.00E-06	2.30
**pf13hi**	1.00E-06	1.01E-06	1.00E-06	1.02E-06	1.00E-06	1.00E-06	1.00E-06	0.01
**pf13ehi**	1.00E-06	1.00E-06	1.00E-06	1.00E-06	1.00E-06	1.00E-06	1.00E-06	0.00
**pf31ei**	1.51E-01	2.22E-03	1.52E-03	8.55E-04	1.25E-01	4.45E-04	3.68E-01	2.92

**pf31hi**	3.58E-04	2.11E-03	4.42E-03	6.15E-02	1.56E-03	5.61E-03	1.06E-03	2.23
**pf31ehi**	2.92E-03	5.48E-05	6.37E-04	3.75E-04	1.24E-04	1.05E-06	4.30E-06	3.44
**ku23hs**	8.23E+01	1.45E+01	1.19E+02	1.23E+01	1.85E+02	2.16E+00	9.59E+01	1.93
**ku23hi**	1.18E+02	3.22E+02	1.63E+01	1.98E+01	1.30E+02	3.85E+00	1.69E+02	1.92
**pf23hs**	1.36E-01	2.25E-02	4.04E-01	6.96E-03	6.72E-01	5.83E-03	7.70E-02	2.06
**pf32hs**	9.22E-02	1.53E-02	3.49E-01	6.50E-03	5.27E-01	5.51E-03	5.74E-02	1.98
**pf23hi**	4.23E-01	2.05E-01	5.40E-02	2.17E-01	1.62E-01	5.41E-02	8.68E-01	1.21
**pf32hi**	4.52E-01	1.77E-01	3.96E-02	1.47E-01	1.16E-01	3.74E-02	6.76E-01	1.26

[1] The 7 representative parameter sets obtained from the clustering of the 188 convergent solutions are shown. Units for dimer dissociation rates (ku values) are min^{-1}. The pf values and delta1 value are dimensionless. The dimer type and cell compartment are specified in the individual dimer dissociation and pf parameters. For e.g., ku11es is the dissociation rate for the R11E dimer (EGFR homodimer with a single bound EGF) at the cell surface

[2] Parameter Set 7 is the best overall fit, i.e., it is the solution with the lowest RMSE

[3] Order of magnitude variation in parameter value between the 7 solutions [log10(Max(Par)) − log10(Min(Par))]. Parameters showing a greater than 2-orders of magnitude variation among the 7 parameter sets are highlighted

Table S5. HER expression levels in various cell lines

**CellLine** *	**Receptor expression (#/cell)**		
	**EGFR**	**HER2**	**HER3**
HBL100 [1; 1 ;1]	68177	0	0
MDAMB468 [2; 2, 3 ;3]	3389807	842	17029
184A1N4 [1; 1 ;1]	248476	1273	0
HS578TT [1; 1 ;1]	119835	12678	0
184B5 [1; 1 ;1]	152390	14513	0
5F12A [1; 1 ;1]	45401	17135	0
BT549 [1; 1 ;1]	146893	20151	0
Parental (HER2−3−) [4; 4 ;4]	**200000**	**30000**	**973**
B5 (HER2−3+) [4; 4 ;4]	**189126**	**30000**	**28000**
MDAMB231 [1; 3 ;3]	196971	37524	1869
ADRr [5; 5 ;5]	177818	40792	33205
ACHN [5; 5 ;5]	448284	45456	15200
5F7 [1; 3 ;3]	17674	46349	29584
MALME3M [5; 5 ;5]	2914	56422	67367
HCC1500 [1; 1 ;1]	280272	56727	0
5F10A [1; 1 ;1]	39874	58038	0
T47D [1; 3 ;3]	14941	66247	32333
BT20 [1; 1 ;1]	106381	69443	0
HCC1143 [1; 1 ;1]	232836	104840	38000
MDAMB435 [1; 1 ;1]	14941	112575	38000
CAMA1 [1; 1 ;1]	19436	113361	38000
600MPE [1; 1 ;1]	25206	141547	38000
HCC70 [1; 1 ;1]	171036	141941	0

LS180 [5; 5 ;5]	122520	143339	28841
MDAMB134VI [1; 1 ;1]	14941	148889	38000
IGROV1 [5; 5 ;5]	149031	158418	53555
ZR751 [5; 5 ;5]	37409	199132	39492
HCC1599 [1; 1 ;1]	15002	200148	38000
LY2 [1; 1 ;1]	15002	208669	38000
BT483 [1; 1 ;1]	14941	215224	38000
HCC38 [1; 1 ;1]	82997	236200	38000
MDAMB415 [1; 1 ;1]	14941	293883	38000
MDAMB157 [1; 1 ;1]	289292	296767	0
MDAMB453 [2; 2, 3 ;3]	5316	345911	48116
MDAMB361 [5; 5 ;5]	65855	371731	32981
ZR75B [1; 1 ;1]	31401	443072	38000
MDAMB175VII [1; 1 ;1]	24629	555161	0
24H (HER2+3−) [4; 4 ;4]	**136938**	**600000**	**2000**
D20 (HER2+3+) [4; 4 ;4]	**85896**	**643777**	**28770**
ADRrE2 [5; 5 ;5]	271000	722000	34400
HCC1187 [1; 1 ;1]	173010	819717	0
SKOV3 [2, 5; 2, 5, 3 ;5, 3]	325952	943647	6929
HCC1569 [1; 1 ;1]	144524	1004565	38000
CALU3 [5; 5 ;5]	161357	1196976	30031
NCIN87 [5; 5 ;5]	417753	1233479	34678
UACC812 [1; 1 ;1]	136902	1240541	38000
HCC202 [1; 1 ;1]	371712	1423160	38000
AU565 [2; 2 ;1]	204560	1447688	38000
BT474 [5; 5, 3 ;5, 3]	129436	1576653	40587
SKBR3 [2; 2, 3 ;3]	143599	1620770	37347
ZR7530 [5; 5 ;5]	3091	2395485	43897
SUM225CWN [1; 1 ;1]	577521	2582327	38000

Direct measurements of absolute receptor numbers (using quantitative FACS) were used where available. Numbers from multiple literature sources were averaged as necessary. When absolute measurements were unavailable, Western blot-based measurements were converted to receptor numbers as described in Supplemental Text.

* The superscripts next to each cell line name contain information about how the estimates were obtained for the 3 receptor types. Superscripts are of the form "<i>; <j>; <k>" where <i>, <j> and <k> refer to the sources of the number for HER1, HER2 and HER3, respectively. The sources are as follows:

1: Estimate was based on the Western blot measurements reported in Neve et al, 2006 [8]. These were converted to absolute numbers as described in Supplemental Text.

2: Defazio-Eli et al [5]

3: Mukherjee et al [7]

4: Current study. In this case ELISA measurements of receptor mass made here were converted to absolute receptor numbers as described in our previous work [4]

5: McDonagh et al. [6]

SUPPLEMENTAL FIGURES:

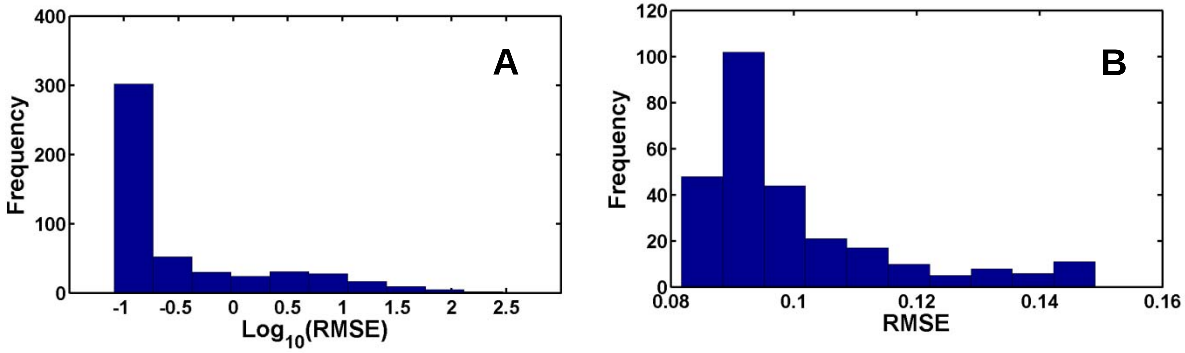

Figure S1. Goodness of fit for the 500 distinct optimization runs. Histogram distributions of the root-mean-squared error (RMSE) between model predictions and the experimental dataset. A) Distribution of RMSE values from all 500 runs plotted on a log-scale. B) Distribution for RMSE <= 0.15 plotted on a linear scale. Based on these results we deemed the 188 solutions with RMSE <= 0.1 to have converged, and selected these parameter sets for further analysis

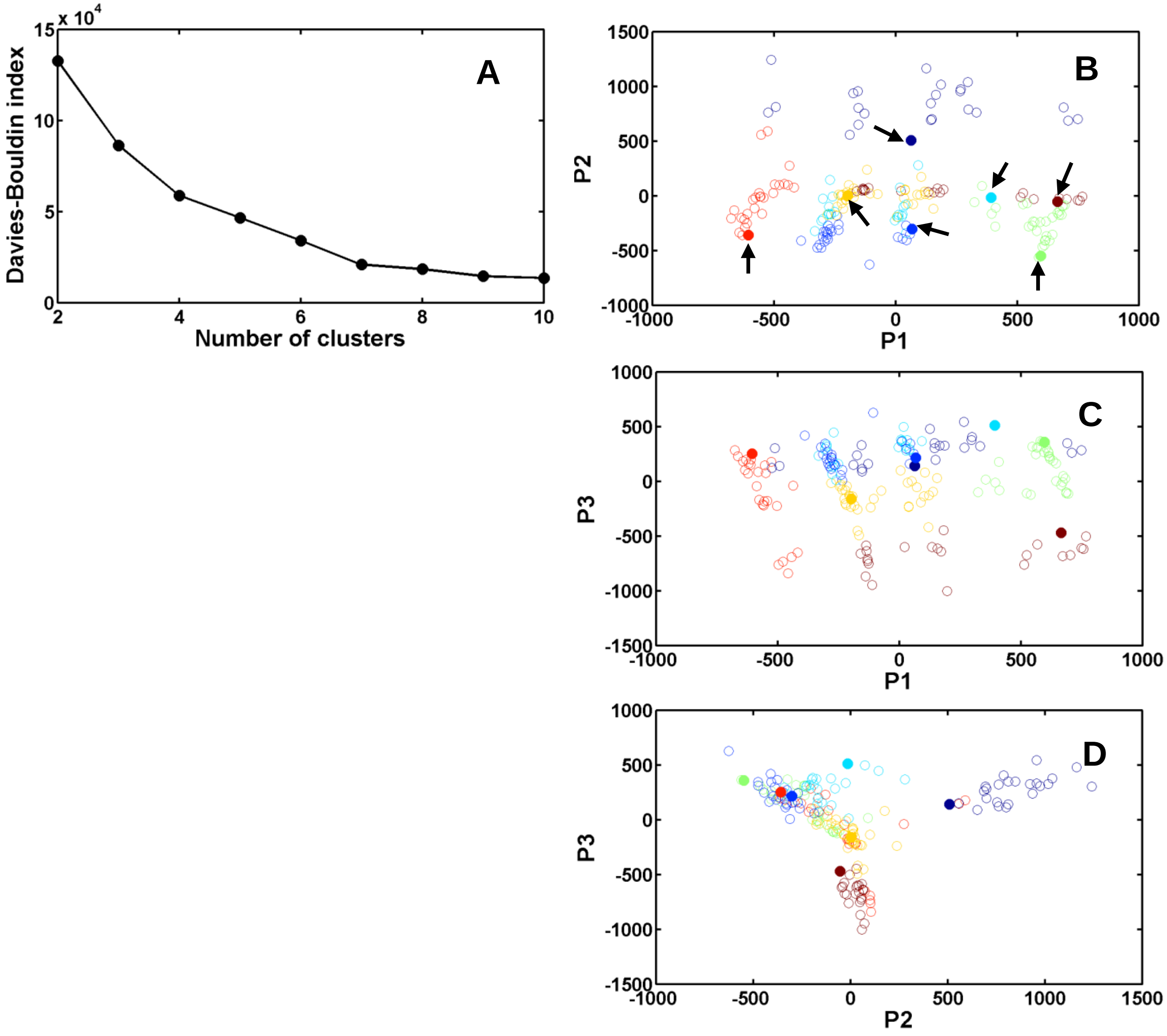

Figure S2. Clustering of parameter values and selection of representative parameter sets. Solutions with RMSE <= 0.1 were subjected to *k*-means clustering**.** We varied the number of clusters during the process, and evaluated the quality of the clustering by computing the Davies-Bouldin index. The DB index for a given number of clusters K is given by $DB(K) = 1/K\ \Sigma_{i=1..K}$ { $[(\Delta(C_i) + (\Delta C_j)]/\delta(C_i, C_j)$ } where $\Delta(C_i)$ represents the sum of distances from the objects within cluster i to the centroid of cluster i, and $\delta(C_i,C_j)$ represents the distance between the centroids of cluster i and j. **A)** DB index as a function of the number of clusters. Based on these results we chose to partition the solutions into 7 distinct clusters. **B-D)** The 188 parameter sets are shown projected onto the top 3 principal component axes. Solutions are color coded based on their cluster membership. Filled circles represent the solutions that have the smallest RMSE within each of the 7 clusters. These solutions are indicated by the arrows in panel B.

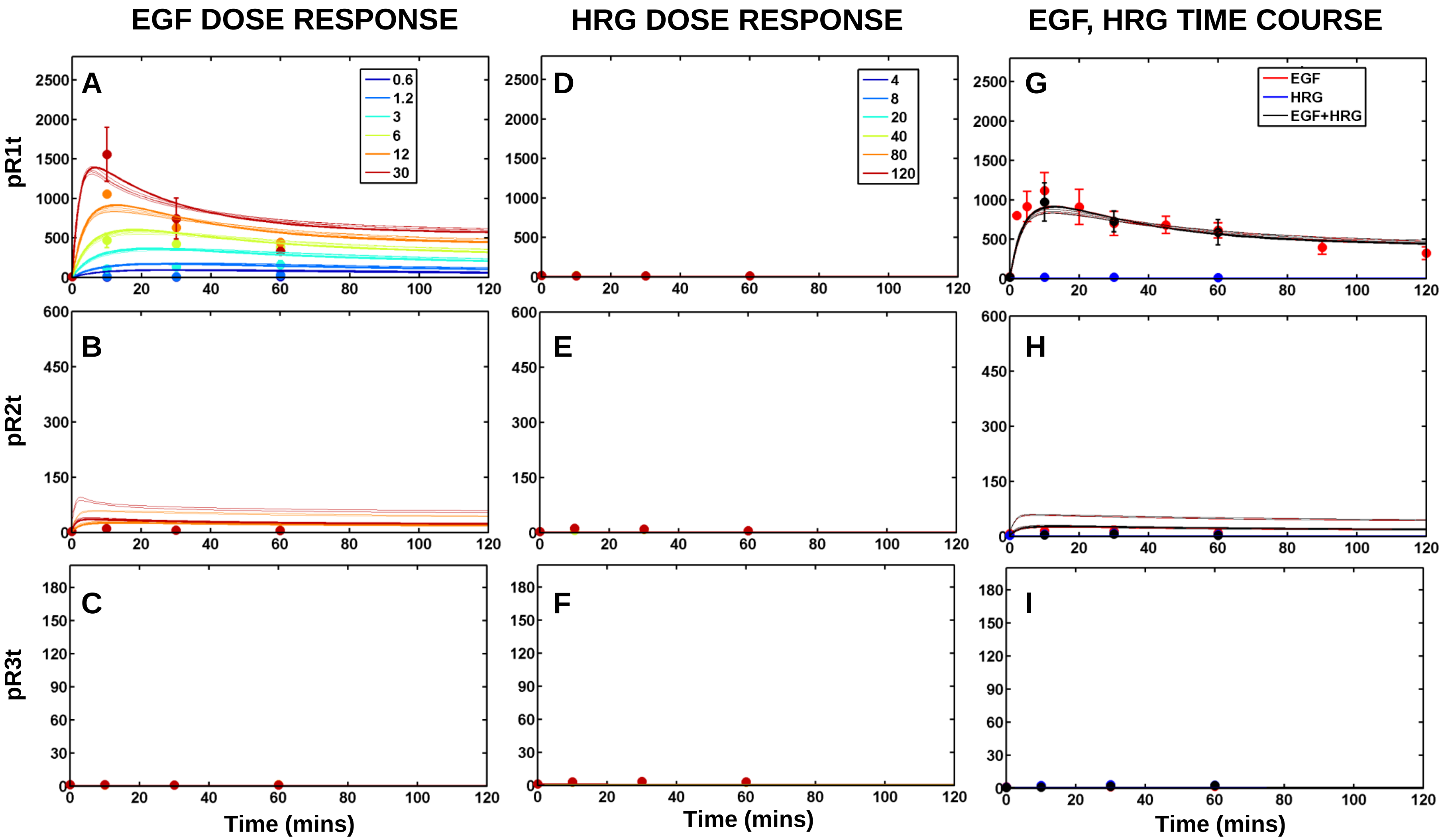

Figure S3. Total receptor phosphorylation in the Parental (HER2-3-) cell line. Experimental data (markers) and model predictions (lines) for total HER phosphorylation. **A-C)** HER1 (A), HER2 (B) and HER3 (C) phosphorylation in response to the indicated doses of EGF, **D-F)** HER1 (D), HER2 (E) and HER3 (F) phosphorylation in response to the indicated doses of HRG, **G-I)** HER1 (G), HER2 (H) and HER3 (I) phosphorylation in response to 12 ng/ml EGF (red), 40 ng/ml HRG (blue) or both (black). For experimental data mean and SD values calculated based on multiple replicates (N>=2) are presented. Model predictions using each of the 7 representative parameter sets are shown (see distinct lines of the same color), with dark lines being used for predictions from the best fit parameter set.

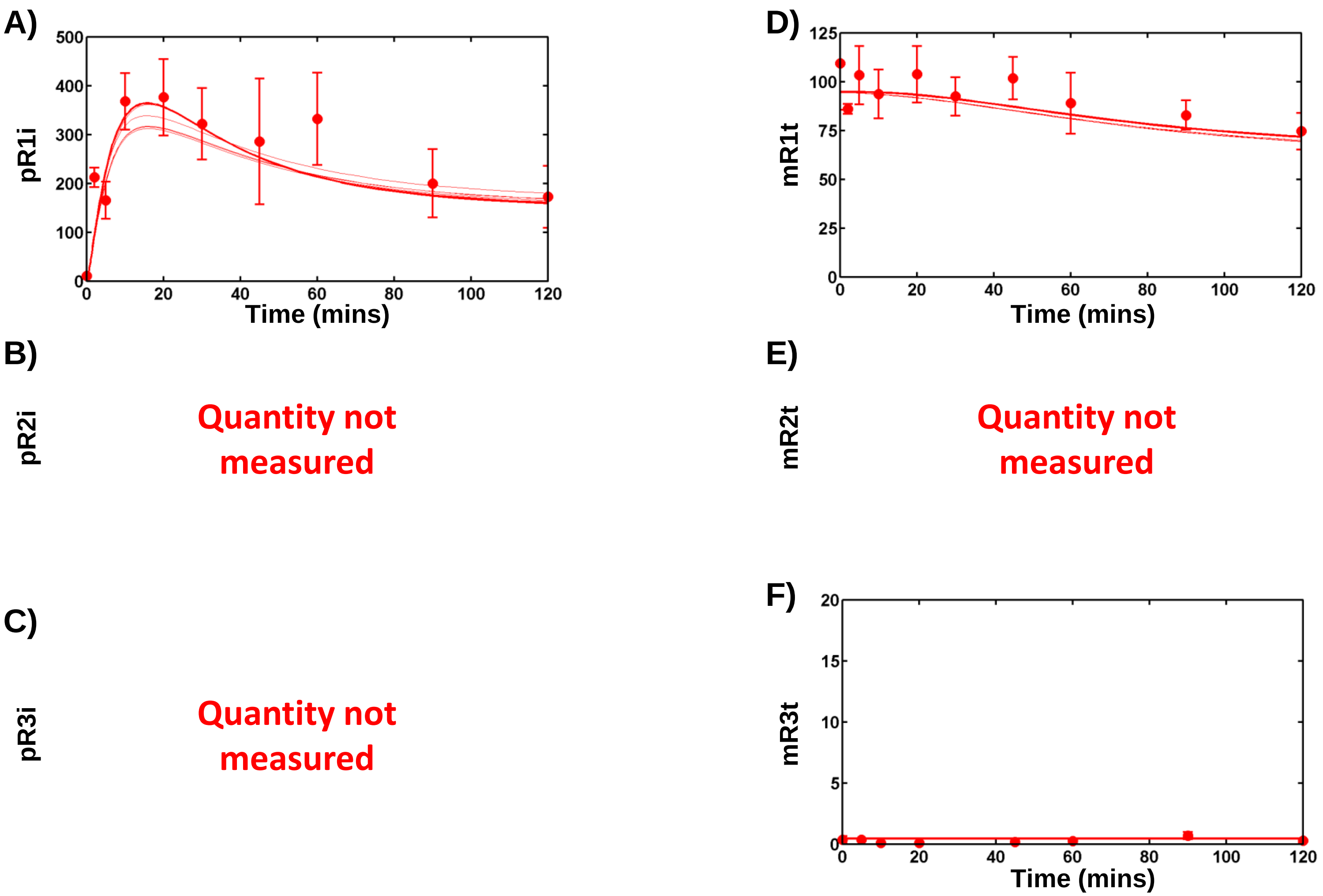

Figure S4. Internal phosphorylation and receptor mass measurements for the Parental (HER2-3-) cell line. A-C) Experimental data (markers) and model predictions (lines) for the levels of EGFR (A), HER2 (B) and HER3 (C) phosphorylation in the cell interior in response to 12 ng/ml EGF (red), 40 ng/ml HRG (blue) or both (black). **D-F)** Experimental data (markers) and model predictions (lines) for the levels of EGFR (D), HER2 (E) and HER3 (F) receptor mass in response to 12 ng/ml EGF (red), 40 ng/ml HRG (blue) or both (black). For experimental data mean and SD values calculated based on multiple replicates (N>=2) are presented. Model predictions using each of the 7 representative parameter sets are shown (see distinct lines of the same color), with dark lines being used for predictions from the best fit parameter set. The absence of a particular line or an entire panel indicates that the quantity was not measured in this cell line. Since the parental cell line expresses very low levels of HER2 and HER3, we did not measure time courses for pR2i (internal pHER2 levels), pR3i (internal HER3 levels) and mR2t (mass of HER2) in this cell line. The low HER3 expression level is evident in panel F.

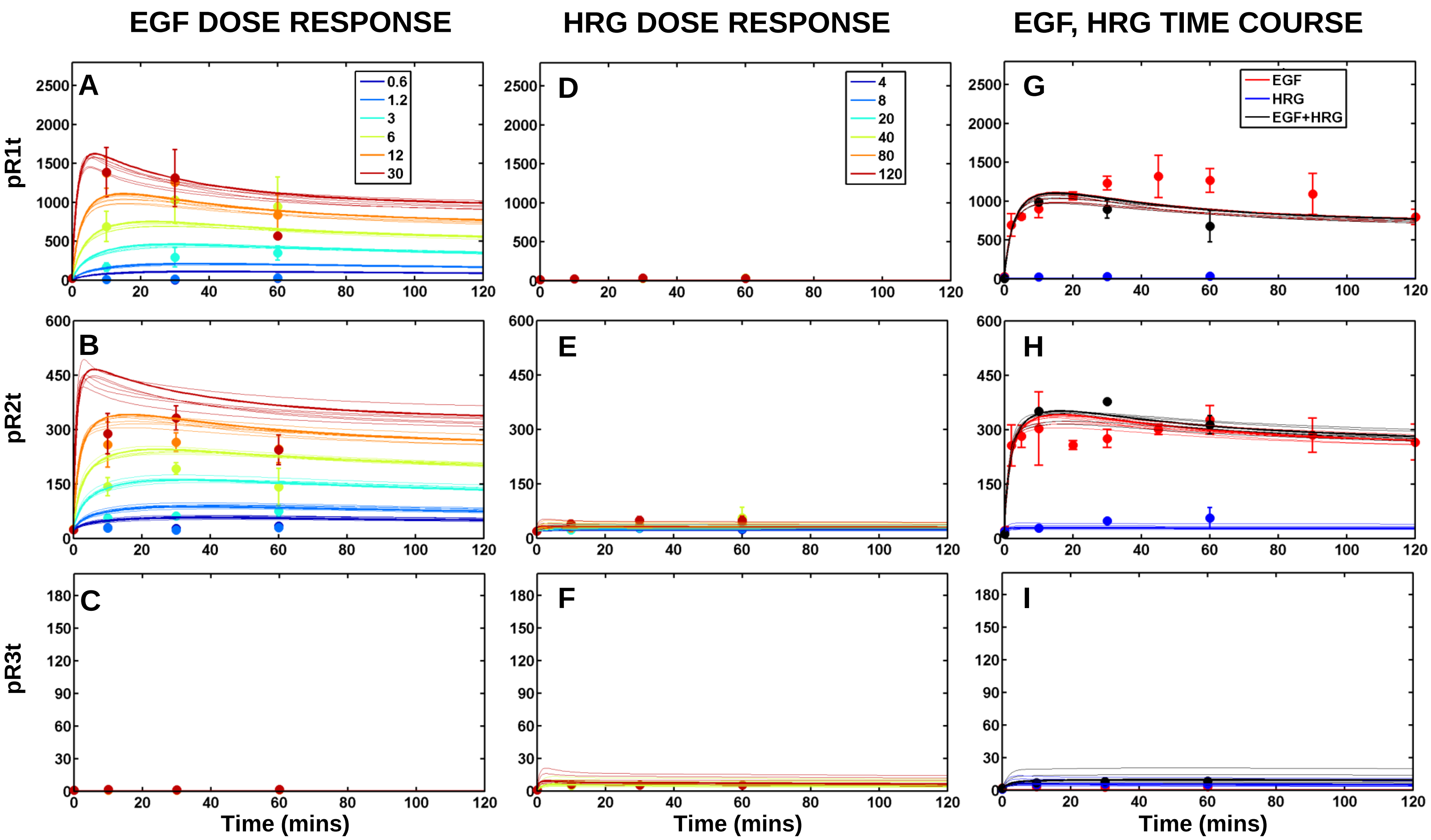

Figure S5. Total receptor phosphorylation in the 24H (HER2+3-) cell line. Experimental data (markers) and model predictions (lines) for total HER phosphorylation. **A-C)** HER1 (A), HER2 (B) and HER3 (C) phosphorylation in response to the indicated doses of EGF, **D-F)** HER1 (D), HER2 (E) and HER3 (F) phosphorylation in response to the indicated doses of HRG, **G-I)** HER1 (G), HER2 (H) and HER3 (I) phosphorylation in response to 12 ng/ml EGF (red), 40 ng/ml HRG (blue) or both (black). For experimental data mean and SD values calculated based on multiple replicates (N>=2) are presented. Model predictions using each of the 7 representative parameter sets are shown (see distinct lines of the same color), with dark lines being used for predictions from the best fit parameter set.

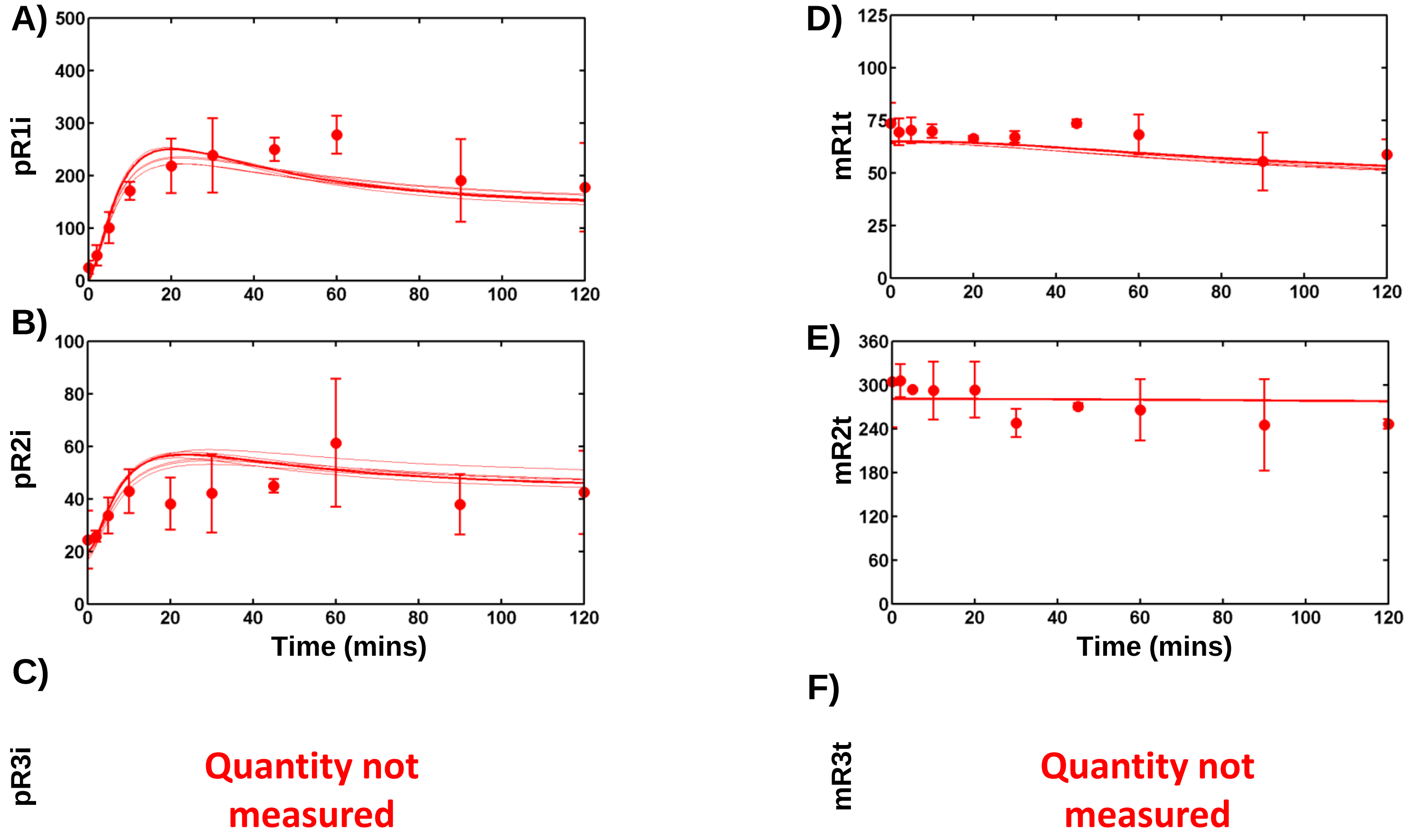

Figure S6 Internal phosphorylation and receptor mass measurements for the 24H (HER2+3-) cell line. A-C) Experimental data (markers) and model predictions (lines) for the levels of EGFR (A), HER2 (B) and HER3 (C) phosphorylation in the cell interior in response to 12 ng/ml EGF (red), 40 ng/ml HRG (blue) or both (black). **D-F)** Experimental data (markers) and model predictions (lines) for the levels of EGFR (D), HER2 (E) and HER3 (F) receptor mass in response to 12 ng/ml EGF (red), 40 ng/ml HRG (blue) or both (black). For experimental data mean and SD values calculated based on multiple replicates (N>=2) are presented. Model predictions using each of the 7 representative parameter sets are shown (see distinct lines of the same color), with dark lines being used for predictions from the best fit parameter set. The absence of a particular line or an entire panel indicates that the quantity was not measured in this cell line. Since the 24H cell line expresses very low levels of HER3, we did not measure time courses for pR3i (internal HER3 levels) and mR3t (mass of HER3) in this cell line.

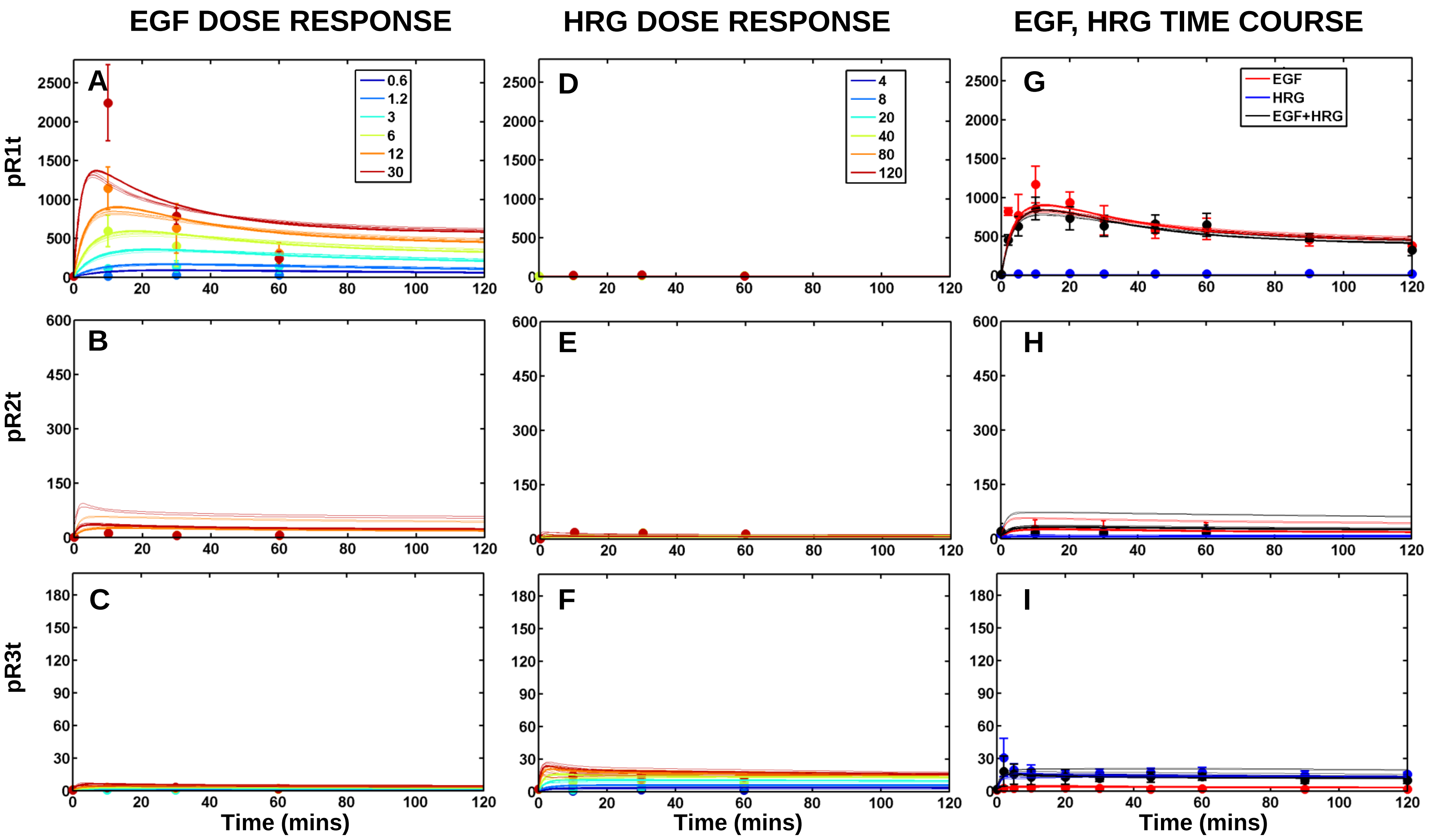

Figure S7. Total receptor phosphorylation in the B5 (HER2-3+) cell line. Experimental data (markers) and model predictions (lines) for total HER phosphorylation. **A-C)** HER1 (A), HER2 (B) and HER3 (C) phosphorylation in response to the indicated doses of EGF, **D-F)** HER1 (D), HER2 (E) and HER3 (F) phosphorylation in response to the indicated doses of HRG, **G-I)** HER1 (G), HER2 (H) and HER3 (I) phosphorylation in response to 12 ng/ml EGF (red), 40 ng/ml HRG (blue) or both (black). For experimental data mean and SD values calculated based on multiple replicates (N>=2) are presented. Model predictions using each of the 7 representative parameter sets are shown (see distinct lines of the same color), with dark lines being used for predictions from the best fit parameter set.

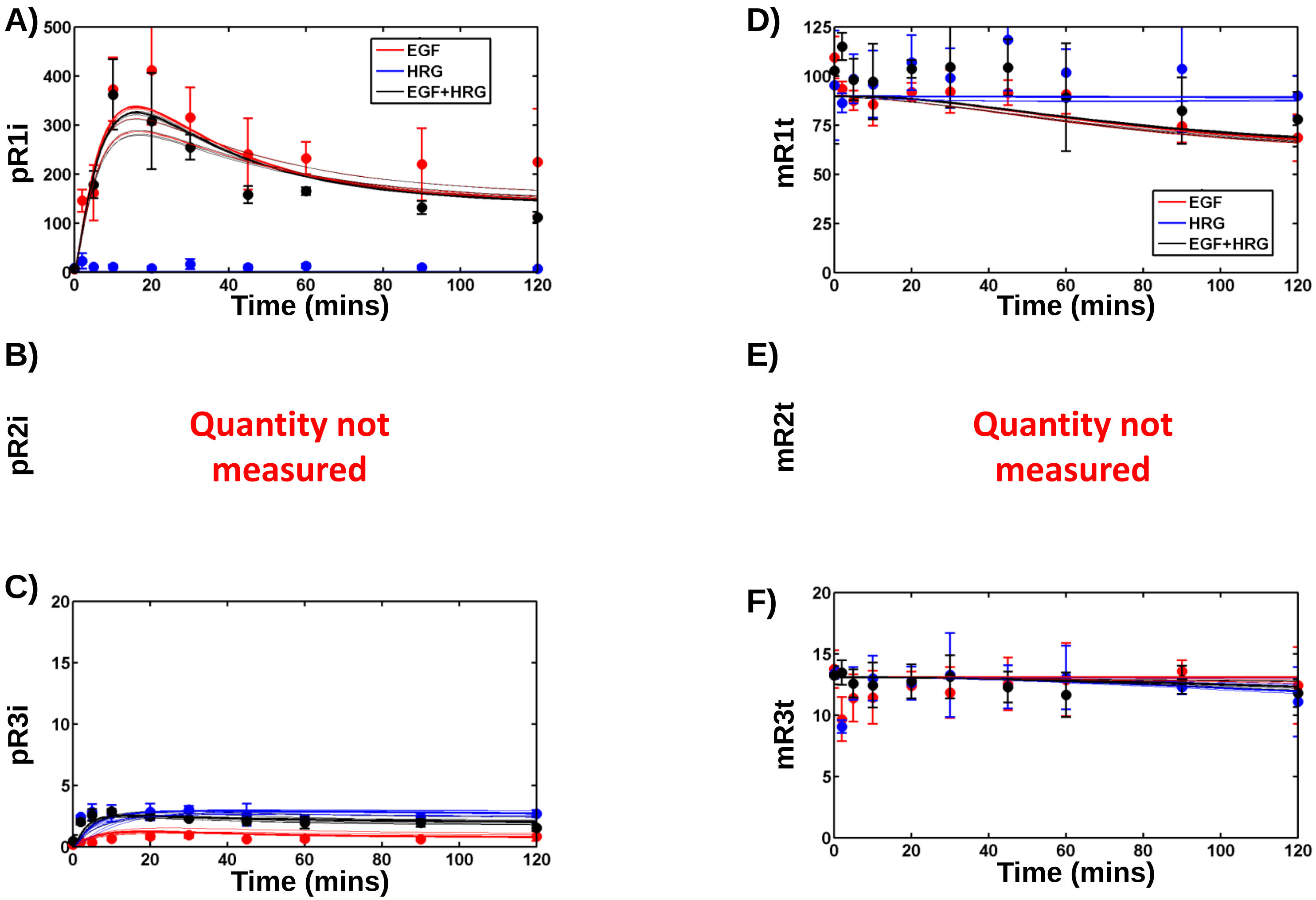

Figure S8. Internal phosphorylation and receptor mass measurements for the B5 (HER2-3+) cell line. A-C) Experimental data (markers) and model predictions (lines) for the levels of EGFR (A), HER2 (B) and HER3 (C) phosphorylation in the cell interior in response to 12 ng/ml EGF (red), 40 ng/ml HRG (blue) or both (black). **D-F)** Experimental data (markers) and model predictions (lines) for the levels of EGFR (D), HER2 (E) and HER3 (F) receptor mass in response to 12 ng/ml EGF (red), 40 ng/ml HRG (blue) or both (black). For experimental data mean and SD values calculated based on multiple replicates (N>=2) are presented. Model predictions using each of the 7 representative parameter sets are shown (see distinct lines of the same color), with dark lines being used for predictions from the best fit parameter set. The absence of a particular line or an entire panel indicates that the quantity was not measured in this cell line. Since the B5 cell line expresses very low levels of HER2, we did not measure time courses for pR2i (internal HER2 levels) and mR2t (mass of HER2) in this cell line.

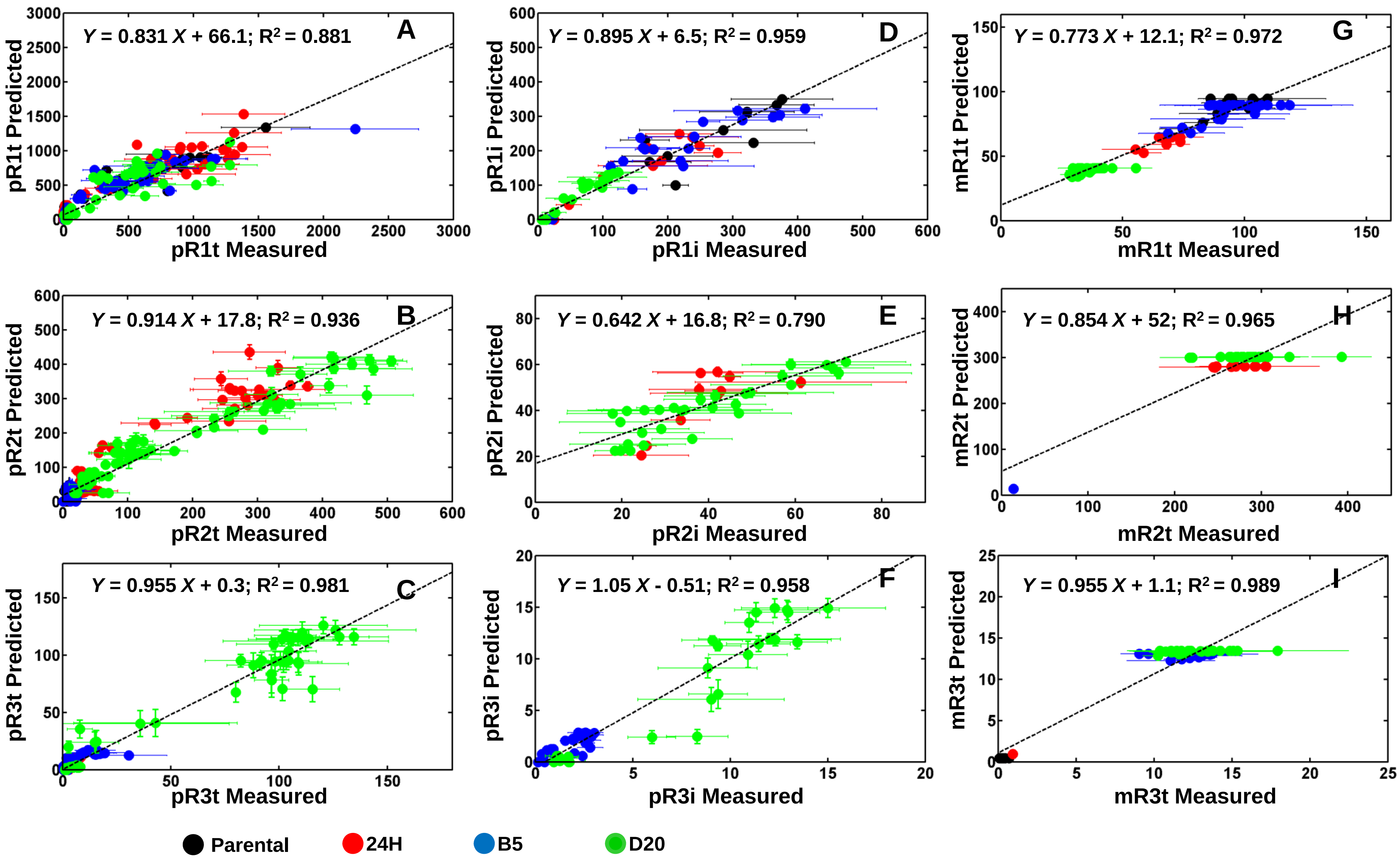

Figure S9. Comparison of model predictions to experimental datasets used for model training. A-C) Model predictions and experimental data for total EGFR (A), HER2 (B) and HER3 (C) phosphorylation, **D-F)** Predictions and data for levels of phosphorylated EGFR (D), HER2 (E) and HER3 (F) in the cell interior, **G-I)** Predictions and data for EGFR (G), HER2 (H) and HER3 (I) receptor mass. For experimental data, mean and SD from multiple replicates are shown; model predictions are mean and SD calculated using results from the 7 distinct parameter sets. Markers are color coded according to the cell line used for the measurements as follows: black – Parental; red – 24H; blue – B5 and green – D20. Black dotted line in each panel represents the linear regression line; Slope, intercept and R^2 from the regression are indicated in each panel.

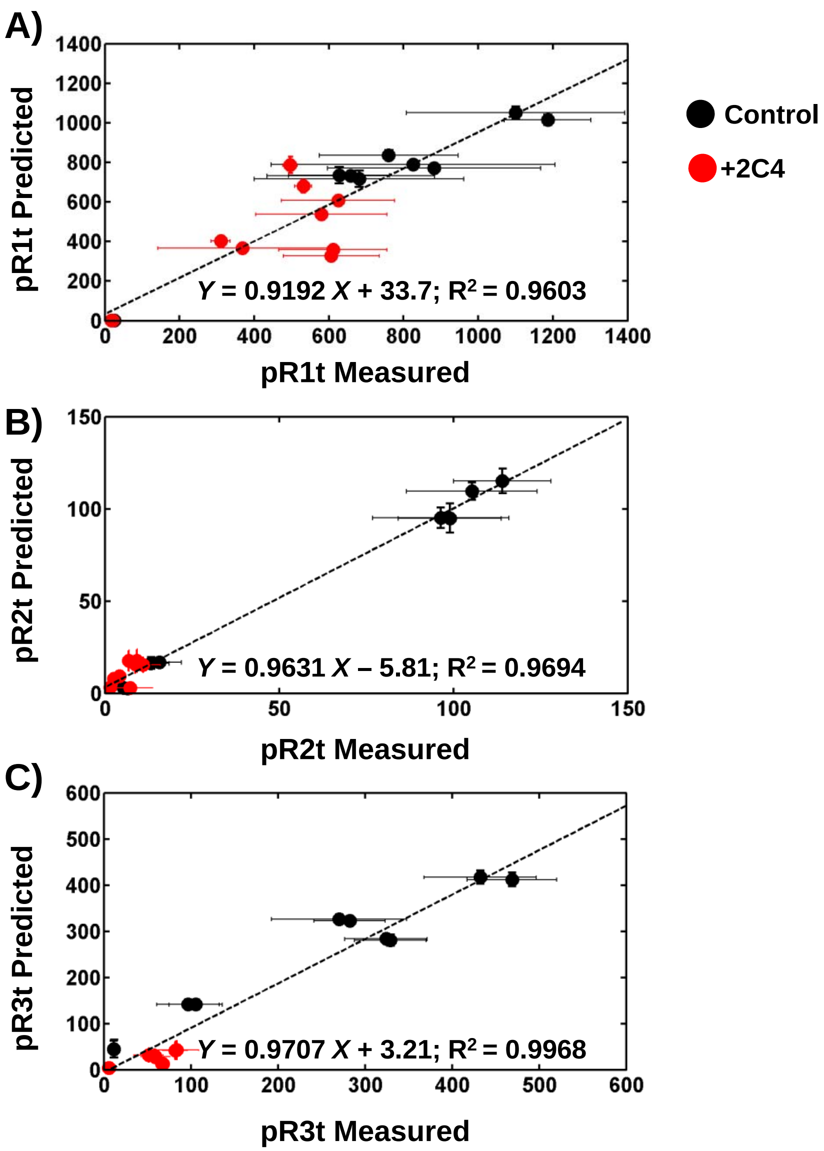

Figure S10. Comparison of model predictions to experimental datasets used for model validation. Comparison of model predictions to experimental data for total **A)** EGFR, **B)** HER2 and **C)** HER3 phosphorylation in the absence (black dots) and presence of the HER2 dimerization blocking antibody 2C4. Data was collected for various cell line and ligand combinations at 10 and 30 min following ligand stimulation (same datasets are plotted in Fig. 5 of the manuscript). For experimental data, mean and SD from multiple replicates are shown; model predictions are mean and SD calculated using results from the 7 distinct parameter sets. Black dotted line in each panel represents the linear regression line; Slope, intercept and R^2 from the regression are indicated in each panel.

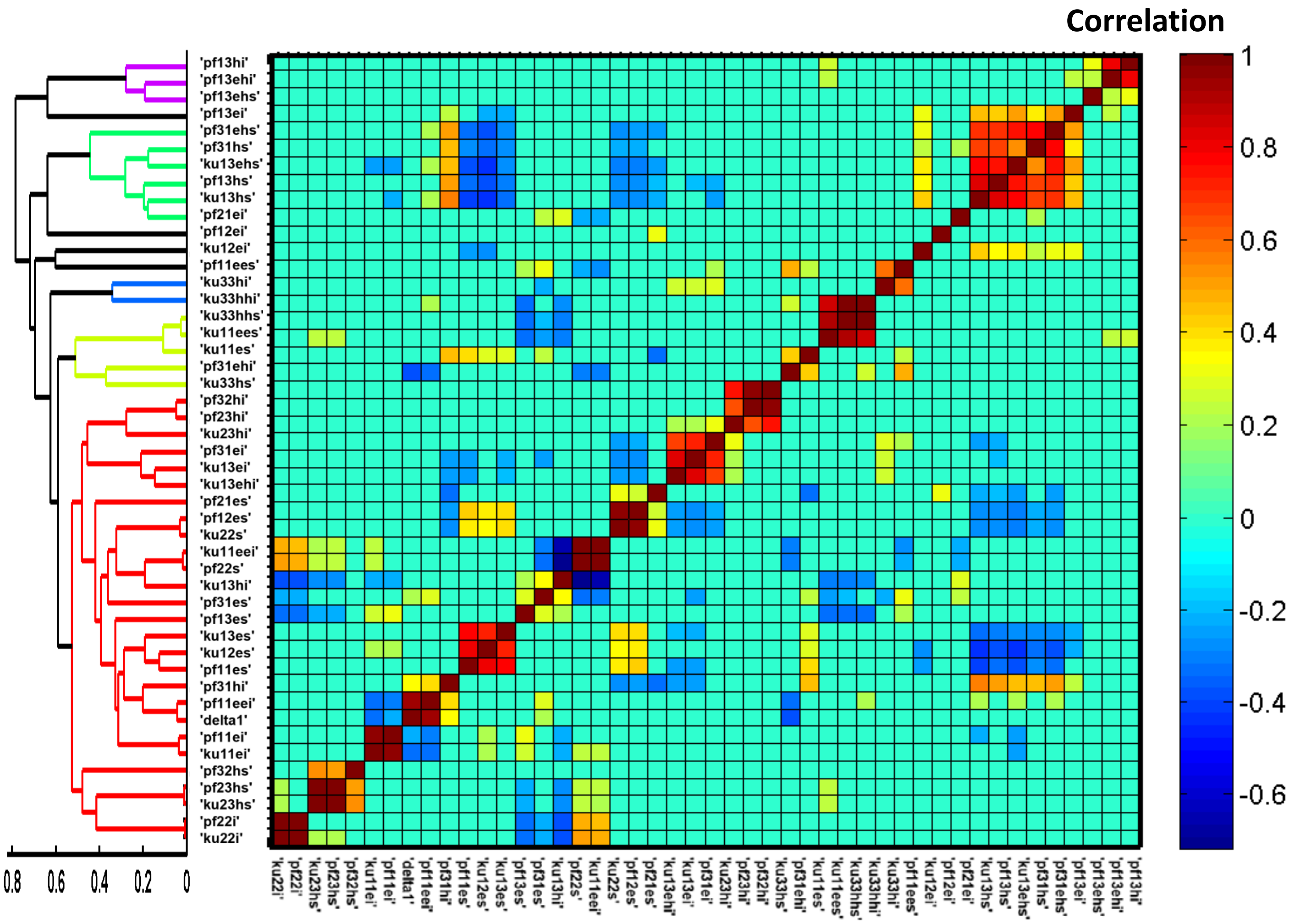

Figure S11. Correlations between model parameters. The pairwise correlation coefficients between the 47 unknown model parameters was calculated using the 188 convergent solutions. To facilitate visualization of parameter correlations, the parameter matrix (rows – parameters; columns – solution vectors) was subjected to hierarchical clustering, and the parameters were ordered based on the clustering results. The dendrogram from the hierarchical clustering is shown to the left of the figure. Correlation coefficients between –0.2 and 0.2 were set to equal 0 in order to simplify the visualization and highlight strong positive and negative correlations.

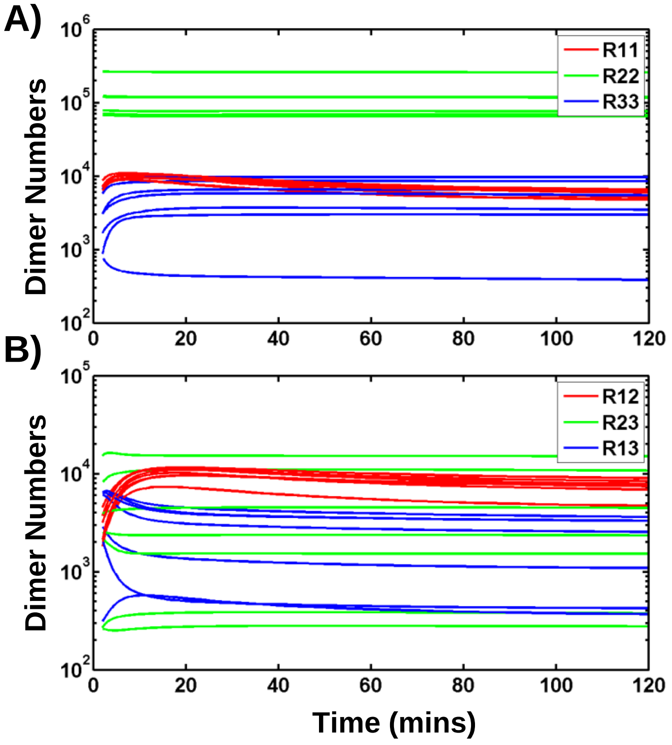

Figure S12. Model predictions for dimer abundance in the HER2+3+ cell line. Absolute numbers of **A)** receptor homodimers, and **B)** heterodimers as a function of time predicted using the 7 representative parameter sets. Colors used for the specific dimer types are indicated in the legend of each panel. Results are shown for the HER2+3+ cell line stimulated with 30 ng/ml EGF and 100 ng/ml HRG. As seen, predictions for the EGFR homodimer (red line in panel A) and EGFR-HER2 heterodimer (red line in panel B) fall within a relatively narrow range, while there is substantial variability in predictions for the other dimer abundances.

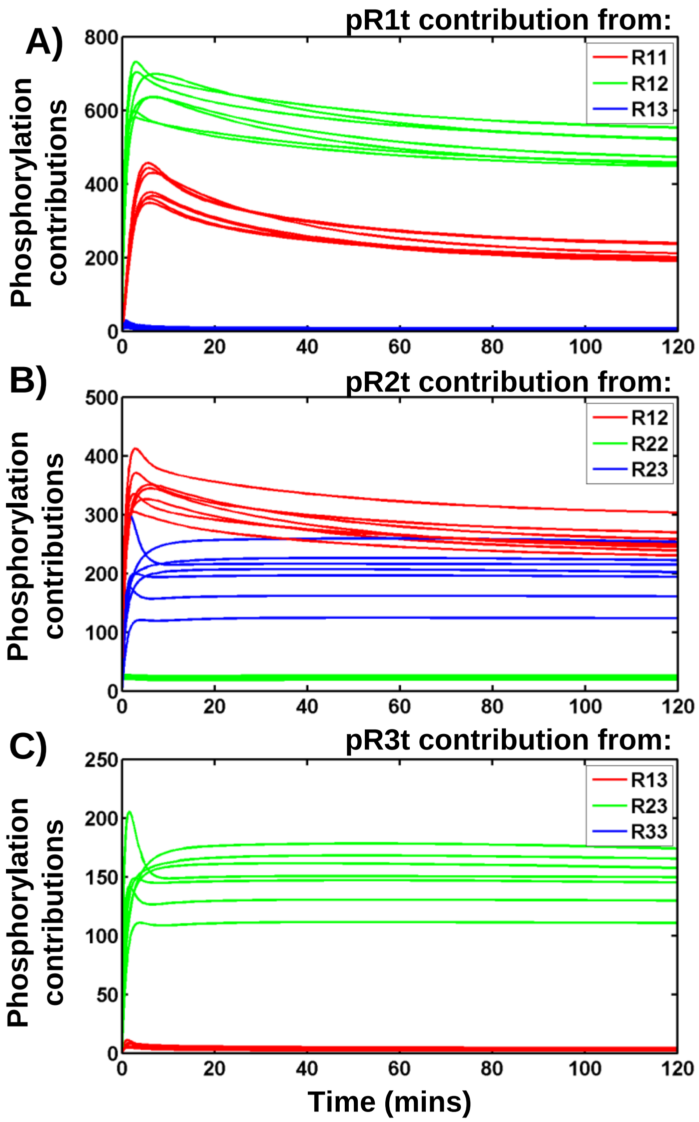

Figure S13. Model predictions for dimer contributions to receptor phosphorylation in the HER2+3+ cell line. The contributions of various relevant dimers to total **A)** EGFR, **B)** HER2 and **C)** HER3 phosphorylation is plotted as a function of time. Colors used for the specific dimer types are indicated in the legend of each panel. Results are shown for the HER2+3+ cell line stimulated with 30 ng/ml EGF and 100 ng/ml HRG. The phosphorylation level from any given dimer was calculated by multiplying the dimer abundance with the appropriate phosphorylation factor (*pf*). The overall phosphorylation level for any given ErbB receptor can be obtained by adding up the contributions from the various dimers shown in the figure. Note that in contrast to Fig. S12, this figure uses a linear Y-axis scale.

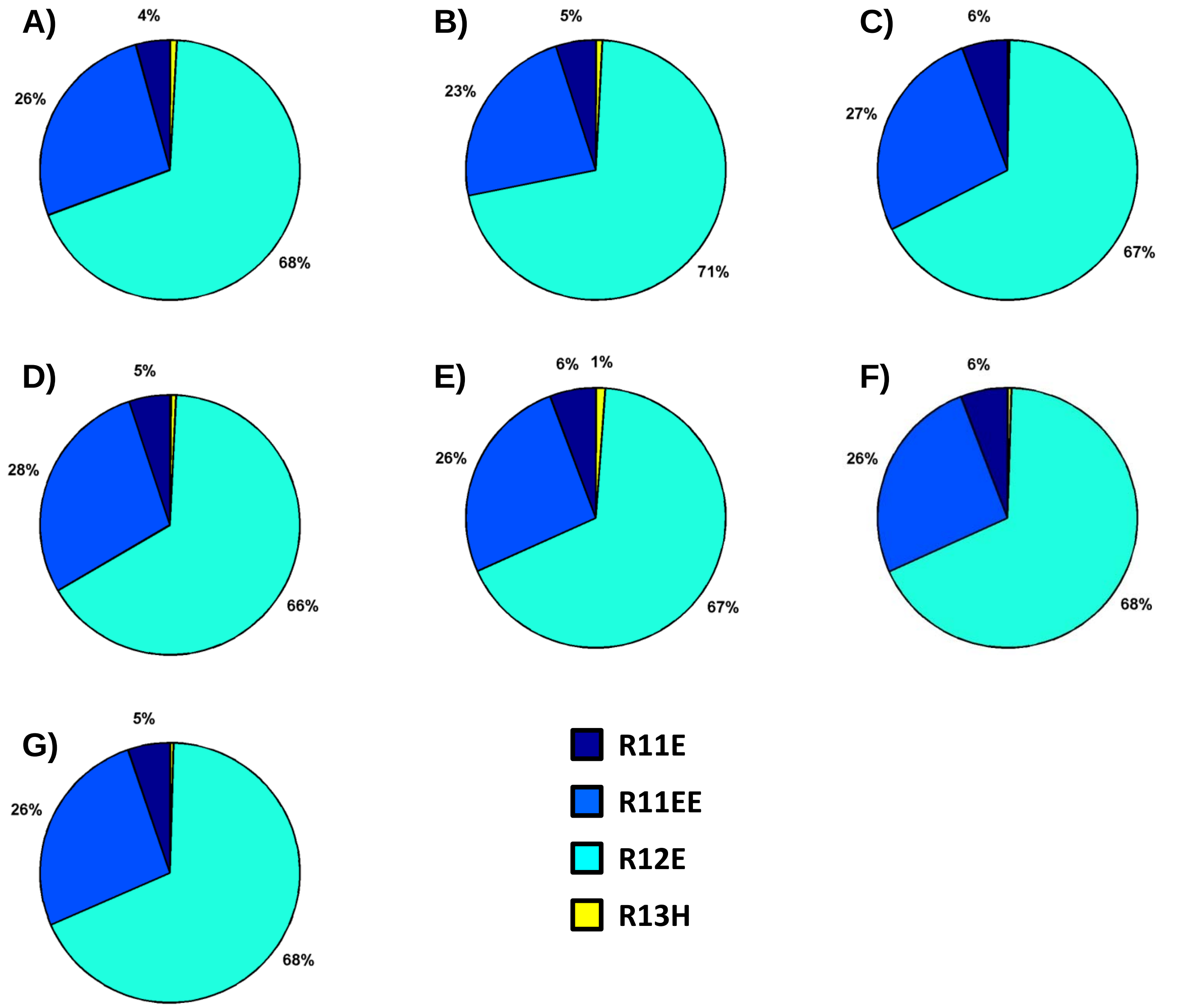

Figure S14. Dimer contributions to EGFR phosphorylation in the HER2+3+ cell line. A-G) Each figure panel contains the prediction from one of the 7 representative parameter sets. Colors used for the various dimer types are indicated in the legend. Results are shown at t=60 min following stimualtion of the HER2+3+ cell line with 30 ng/ml EGF and 100 ng/ml HRG.

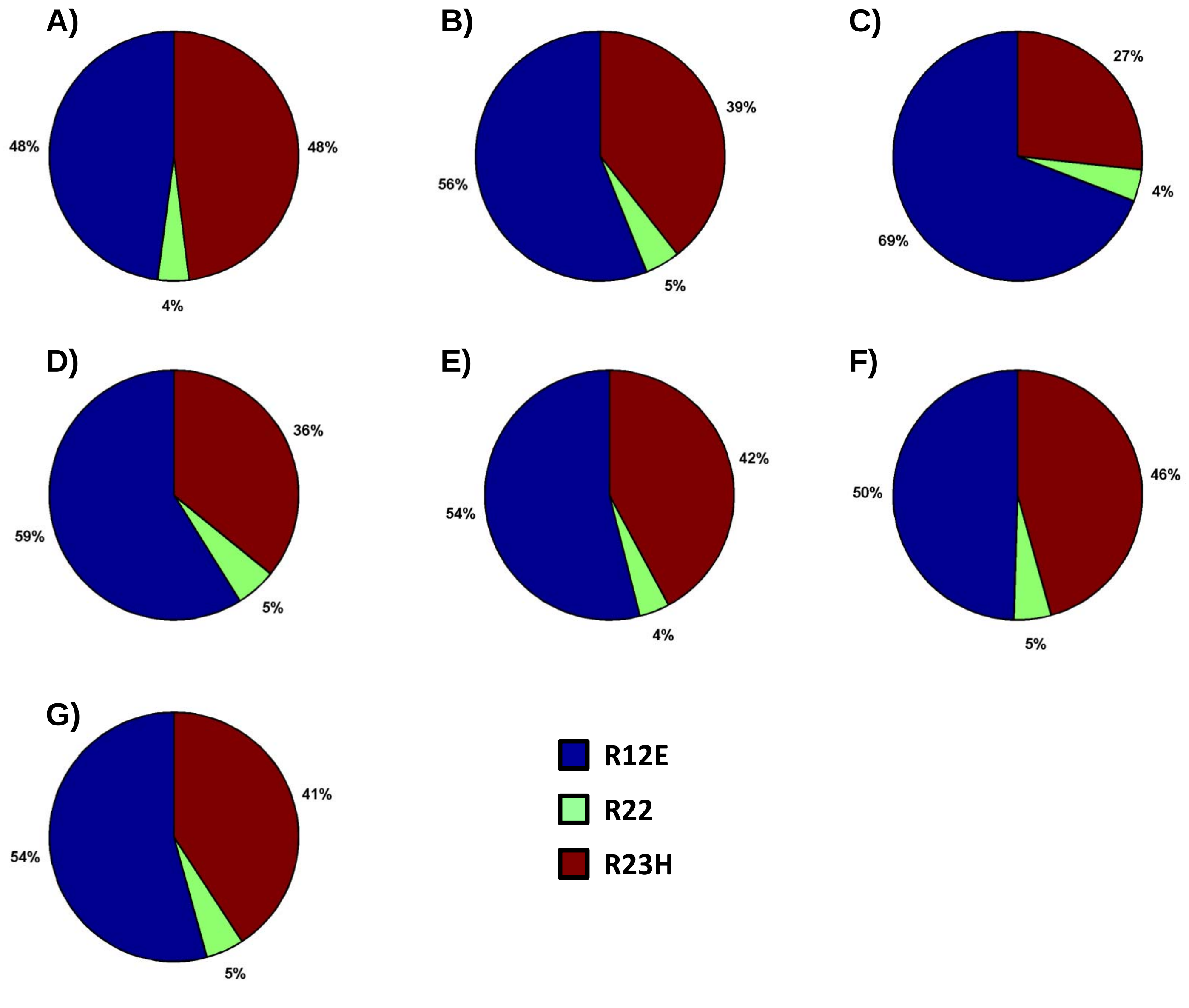

Figure S15: Dimer contributions to HER2 phosphorylation in the HER2+3+ cell line. A-G) Each figure panel contains the prediction from one of the 7 representative parameter sets. Colors used for the various dimer types are indicated in the legend. Results are shown at t=60 min following stimualtion of the HER2+3+ cell line with 30 ng/ml EGF and 100 ng/ml HRG.

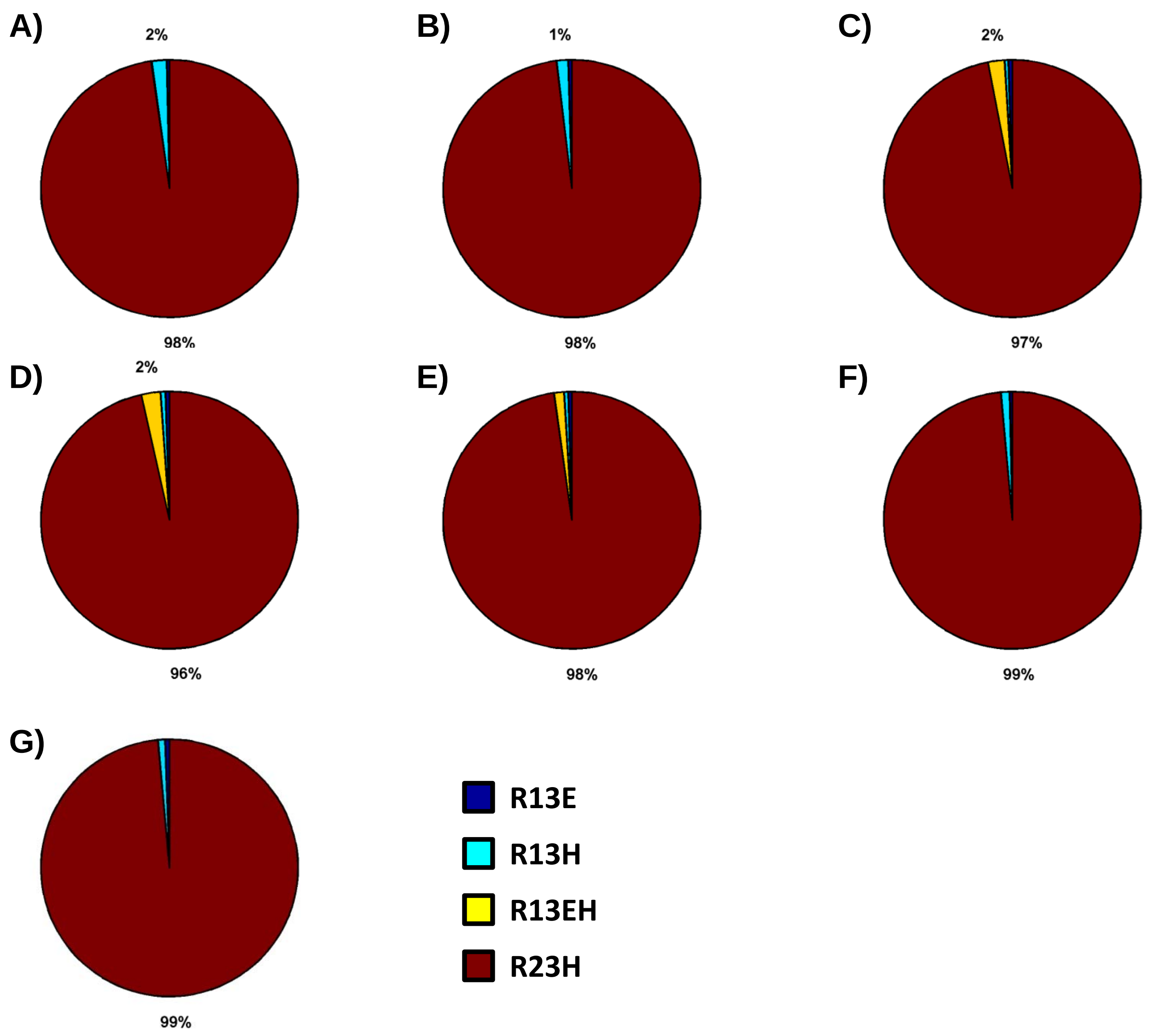

Figure S16. Dimer contributions to HER3 phosphorylation in the HER2+3+ cell line. A-G) Each figure panel contains the prediction from one of the 7 representative parameter sets. Colors used for the various dimer types are indicated in the legend. Results are shown at t=60 min following stimualtion of the HER2+3+ cell line with 30 ng/ml EGF and 100 ng/ml HRG.

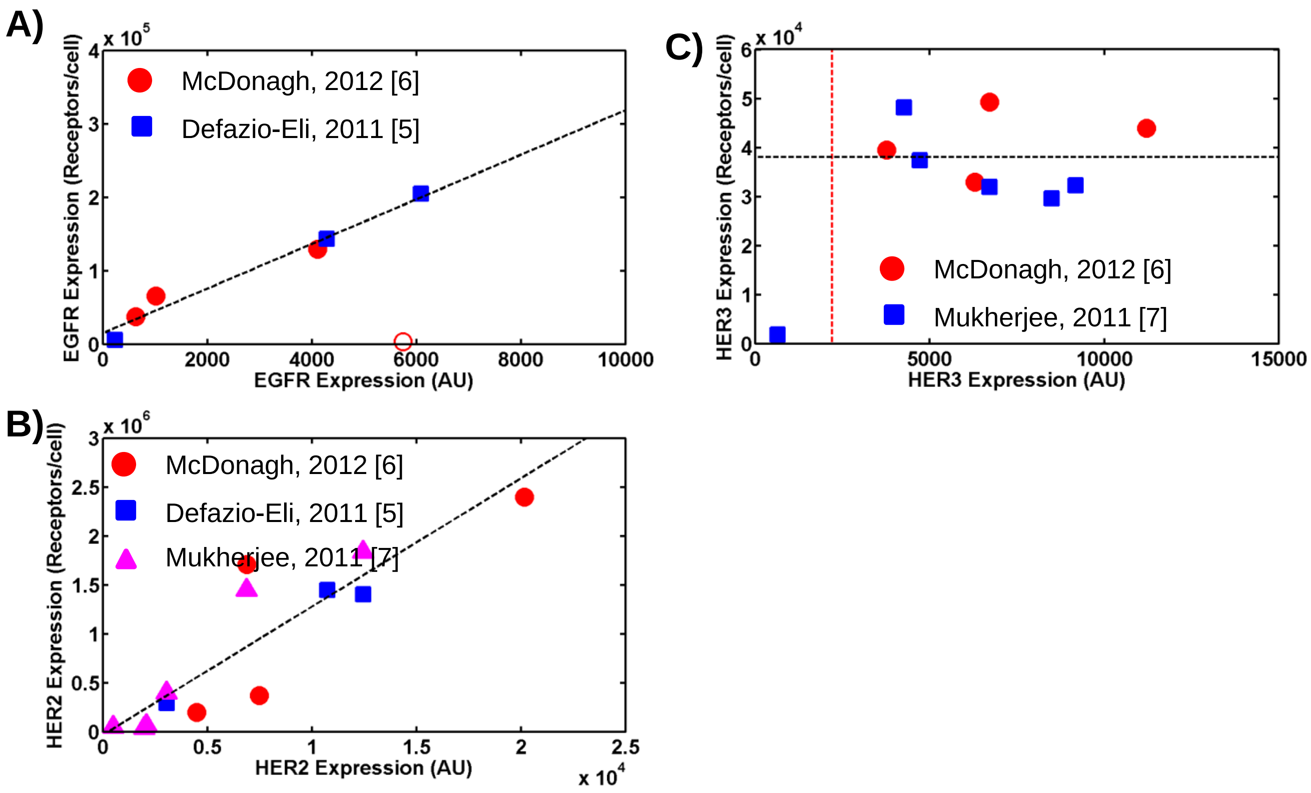

Figure S17. Relationship between absolute receptor numbers and western blot measurements of HER protein levels. Absolute receptor expression levels are plotted against western blot-based relative protein measurements for: **A)** EGFR, **B)** HER2, and **C)** HER3. Each marker represents a measurement made for a particular cell line. The markers are color coded to indicate the distinct data sources from the literature. Black dotted lines in panels A and B are linear regression lines. Red dotted line in panel C indicates a threshold below, which we assumed no HER3 expression and above which we assumed a constant HER3 expression level of 38,000 (mean expression level of HER3+ cells; black dotted line in panel C). See Supplemental Text for additional details

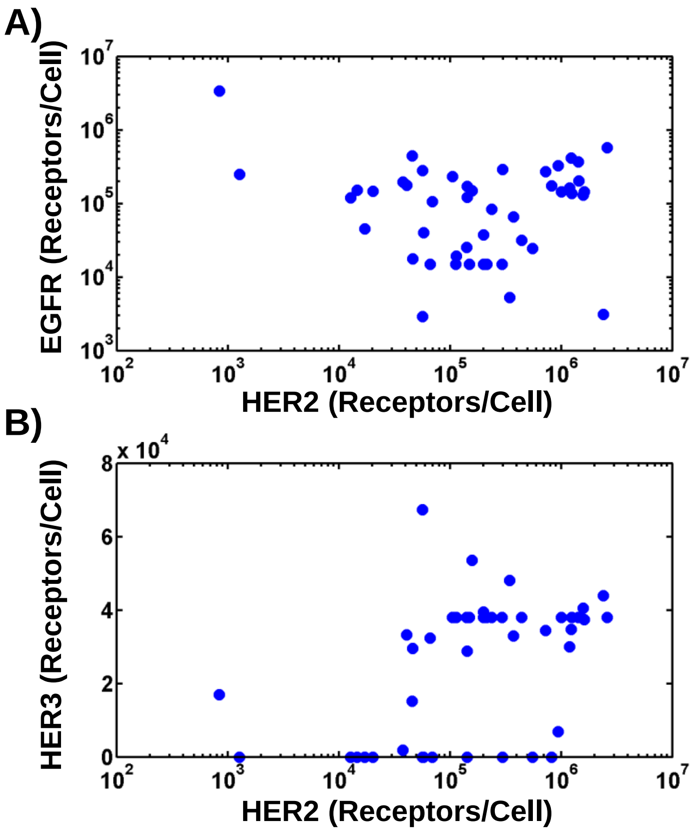

Figure S18. Receptor expression levels estimated for various cell lines. A) EGFR vs. HER2 and **B)** HER3 vs. HER2 receptor expression level plots for 52 distinct cell lines (see Table S5 for absolute numbers). Measurements of absolute receptor numbers were used as such where available. For other cells, western blot measurements from Neve et al. [8] were converted to absolute receptor numbers using the relationships described in Fig. S17. As seen, cells display a relatively wide range of EGFR and HER2 expression levels (panel A). HER3 expressing cells typically express the receptor at ~40,000 molecules/cell (panel B)

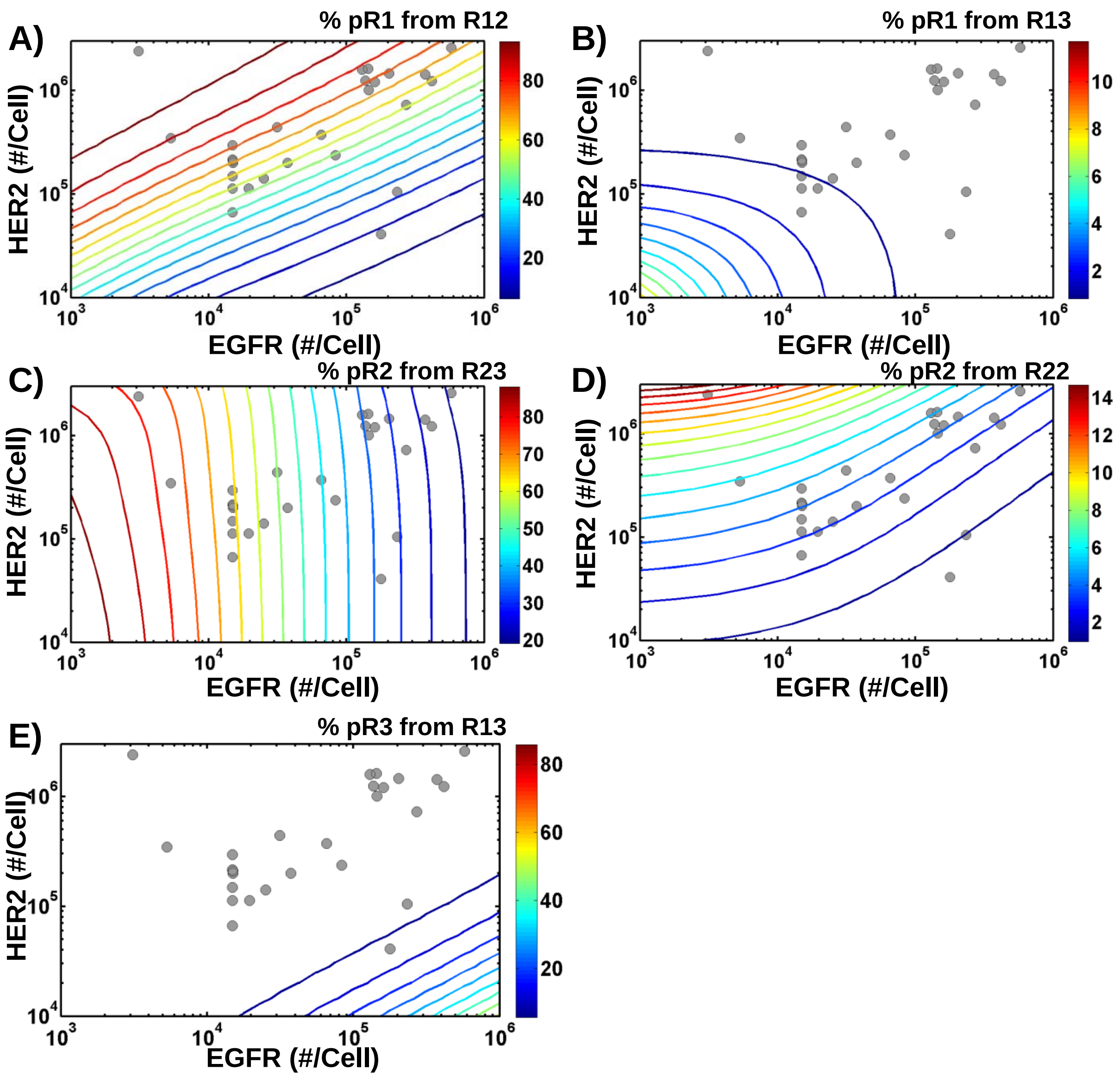

Figure S19: Effect of EGFR and HER2 expression levels on active dimer formation. A-B) Model predictions for % contribution of the R12 (A) and R13 (B) dimers to EGFR phosphorylation, **C-D)** Predictions for % contribution of the R23 (C) and R22 (D) dimers to HER2 phosphorylation, E) Predictions for % contribution of the R13 dimer to HER3 phosphorylation. Contributions of other dimers and the total HER phosphorylation level are in Fig. 7 of the manuscript. Predictions were generated for a fixed HER3 expression of 40,000 receptors/cell. Results are shown for t=60 min following the combined addition of 30 ng/ml EGF and 100 ng/ml HRG. The grey dots in each panel indicate the EGFR and HER2 expression levels of various HER3-expressing cell lines.

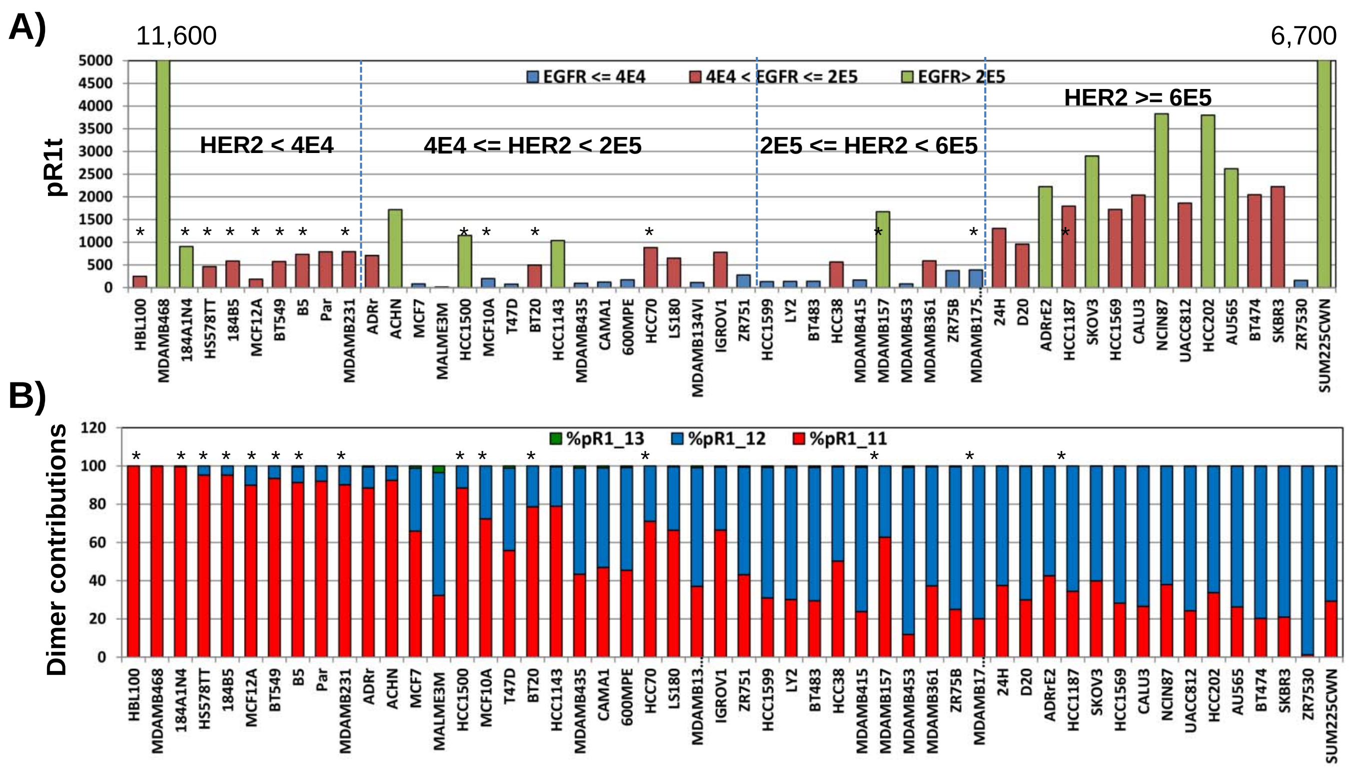

Figure S20. Predictions for EGFR phosphorylation in various cell lines. A) Model predictions for total EGFR phosphorylation. The bars are color coded based on the EGFR expression of the cell line as indicated in the legend at the top of the panel. **B)** Predictions for the % contribution of various relevant dimer types to EGFR phosphorylation. Colors used for each dimer type are indicated in the legend at the top of the panel. In both panels cell lines have been arranged along the x-axis in increasing order of HER2 expression. A “*” on top of a bar in the panels indicates that the cell line expresses very low levels of HER3. As seen, EGFR phosphorylation increases with EGFR and HER2 expression, with the % contribution of the R12 dimer increasing with HER2 expression.

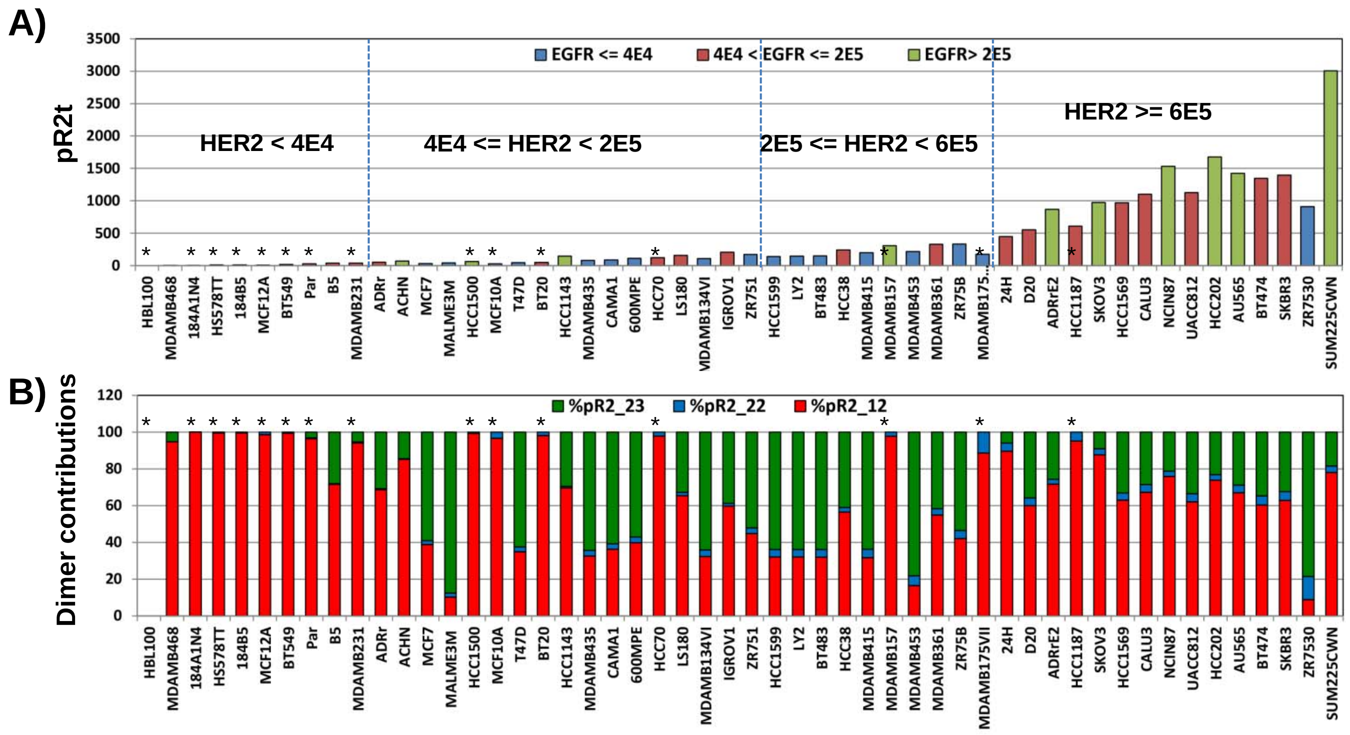

Figure S21: Predictions for HER2 phosphorylation in various cell lines. A) Model predictions for total HER2 phosphorylation. The bars are color coded based on the EGFR expression of the cell line as indicated in the legend at the top of the panel. **B)** Predictions for the % contribution of various relevant dimer types to HER2 phosphorylation. Colors used for each dimer type are indicated in the legend at the top of the panel. In both panels cell lines have been arranged along the x-axis in increasing order of HER2 expression. A "*" on top of a bar in the panels indicates that the cell line expresses very low levels of HER3. As expected, HER2 phosphorylation increases with HER2 expression. The relative contributions of R12 and R23 to HER2 phosphorylation is determined by the EGFR expression level of the cell line (note the relationship between the bar color in panel A, and the dimer contributions in panel B for cells expressing HER2 above 600,000).

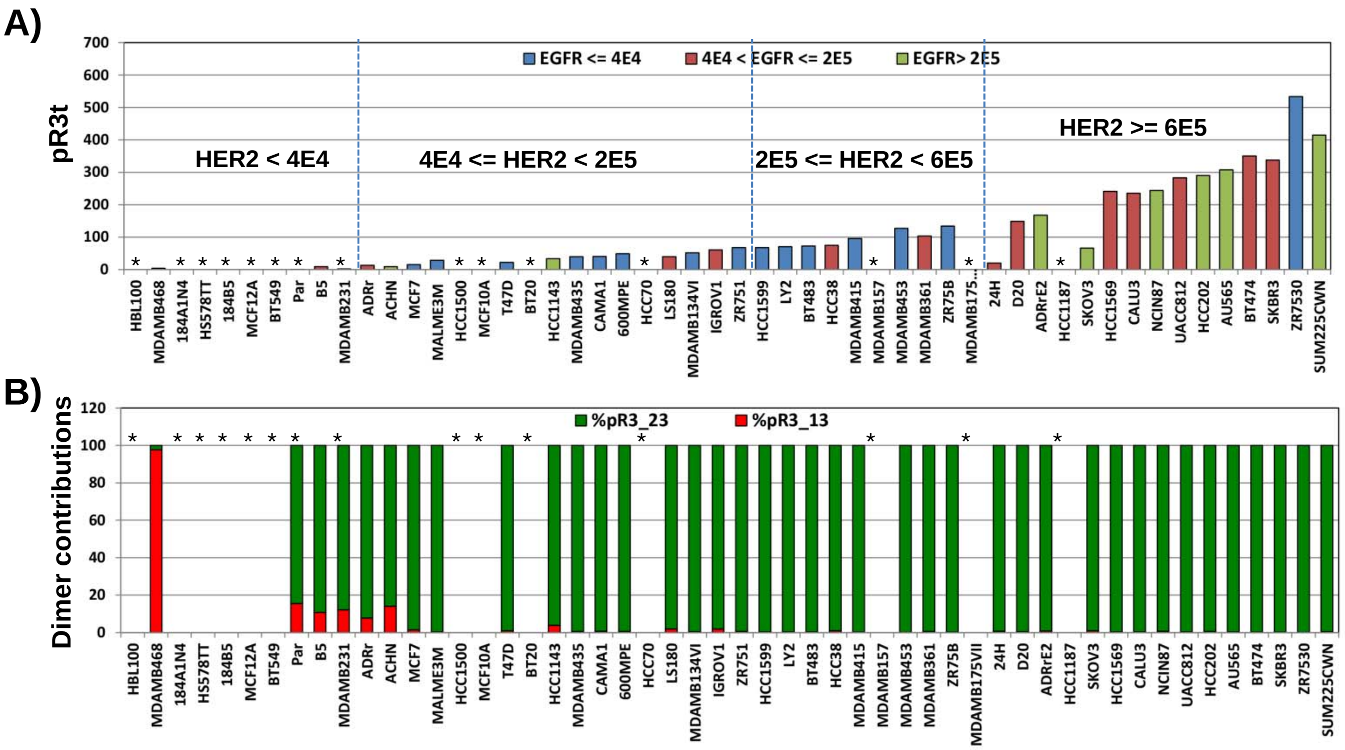

Figure S22. Predictions for HER3 phosphorylation in various cell lines. A) Model predictions for total HER3 phosphorylation. The bars are color coded based on the EGFR expression of the cell line as indicated in the legend at the top of the panel. **B)** Predictions for the % contribution of various relevant dimer types to HER3 phosphorylation. Colors used for each dimer type are indicated in the legend at the top of the panel. In both panels cell lines have been arranged along the x-axis in increasing order of HER2 expression. A “*” on top of a bar in the panels indicates that the cell line expresses very low levels of HER3. As seen, HER3 phosphorylation increases with HER2 expression. In all cases with significant HER3 phosphorylation the signal is almost exclusively from the R23 dimer (see panel B).

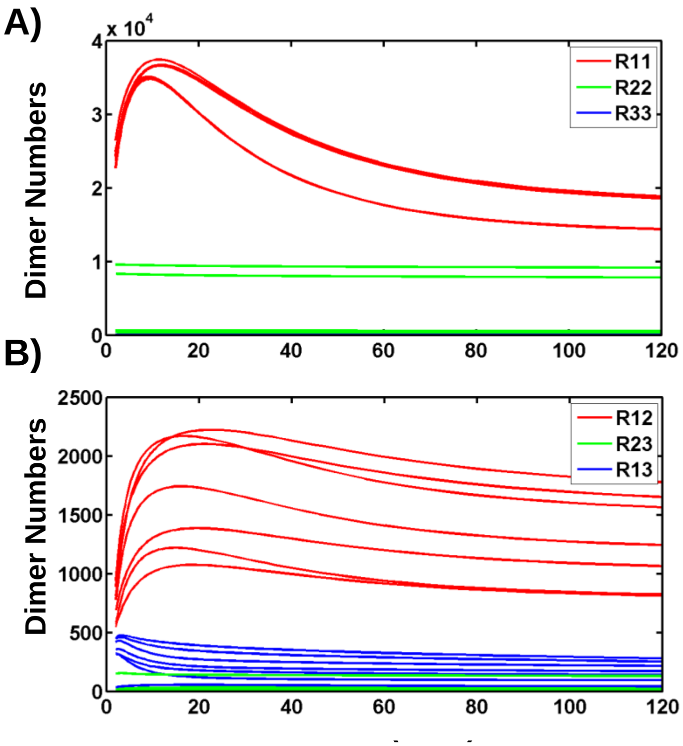

Figure S23: Model predictions for dimer abundance in the Parental cell line. Absolute numbers of **A)** receptor homodimers, and **B)** heterodimers as a function of time predicted using the 7 representative parameter sets. Colors used for the specific dimer types are indicated in the legend of each panel. Results are shown for the Parental cell line stimulated with 30 ng/ml EGF and 100 ng/ml HRG. Results indicate that even at saturating ligand doses, < 80,000 (2× 40,000 R11 dimers) of the total of 200,000 EGFR form dimers (i.e. < 40% of cellular EGFR is in the form of active receptors)

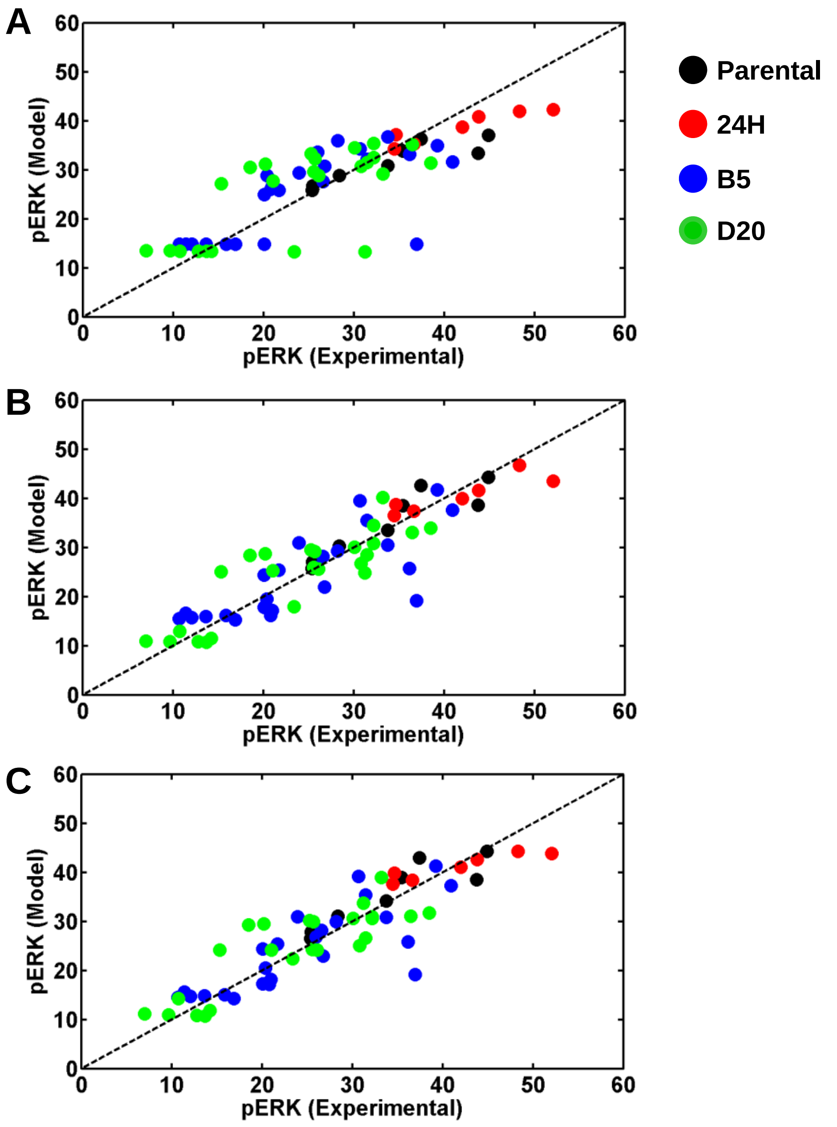

Figure S24. Multilinear regression modeling of ERK activation. Regression model predictions are compared to experimental data for A) ERK Model 1 (see Table S6) that is based on total receptor phosphorylation levels, B) ERK Model 3 that uses all of the dimer signals as the predictors and C) for ERK Model 4 that uses a reduced set of the dimer signals for the prediction. As seen, the dimer-based models outperform the model based on total phosphorylation levels (compare B and C vs. A). Further, the predictive quality of the two dimer-based models are comparable to each other.

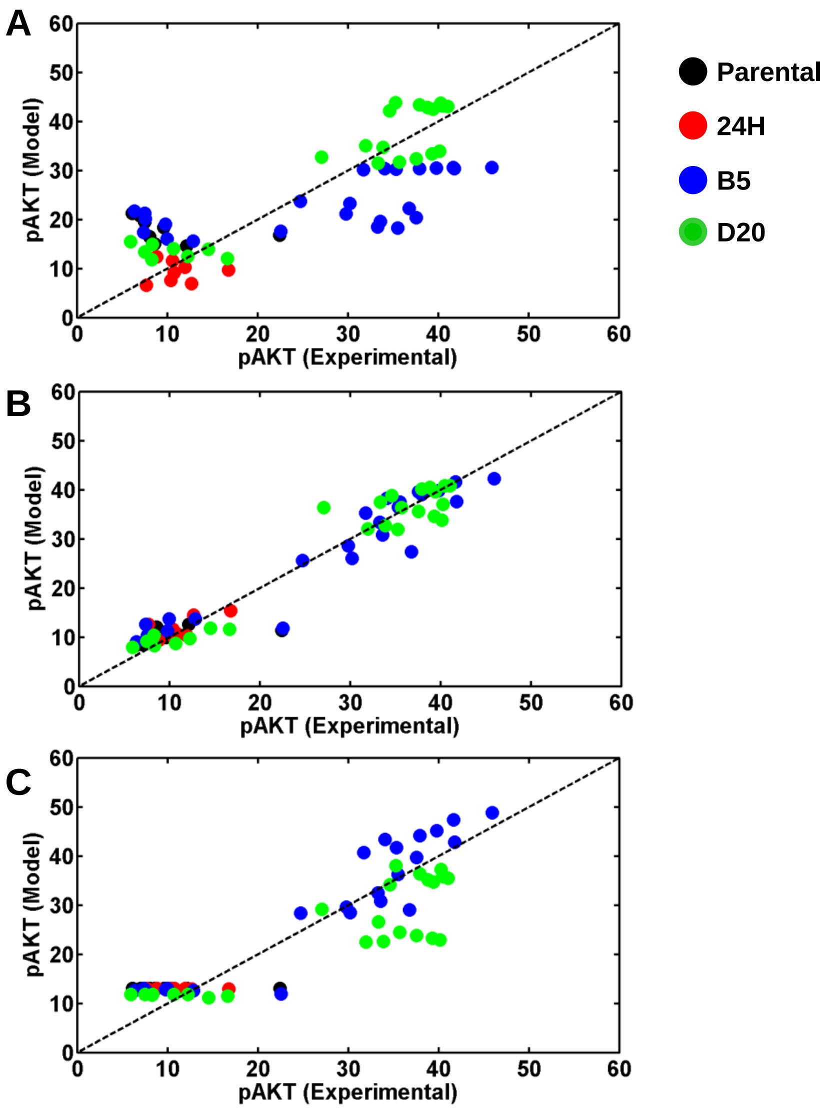

Figure S25. Multilinear regression modeling of AKT activation. Regression model predictions are compared to experimental data for A) AKT Model 1 (see Table S6) that is based on total receptor phosphorylation levels, B) AKT Model 3 that uses all of the dimer signals as the predictors and C) for AKT Model 6 that uses a reduced set of the dimer signals for the prediction. As seen, the dimer-based models outperform the model based on total phosphorylation levels (compare B and C vs. A). This is true even for considerably reduced Model 6, which while being worse than Model 3 (compare C vs. B) still outperforms Model 1 (C vs. A).
